# The combined effects of multiple stressors in an endangered, long‐lived species: Lessons learned and ways forward

**DOI:** 10.1002/eap.70144

**Published:** 2025-12-08

**Authors:** Enrico Pirotta, Peter L. Tyack, Jeffrey Adams, Michael J. Asaro, Phil J. Bouchet, Daniel E. Crocker, John W. Durban, Ailsa J. Hall, Catriona M. Harris, Amy R. Knowlton, Scott D. Kraus, Caroline Lehoux, Daniel W. Linden, Caroline P. Good, Erin Meyer‐Gutbrod, Alicia S. Miller, Carolyn A. Miller, Patrick J. O. Miller, Michael J. Moore, Christopher D. Orphanides, Eric M. Patterson, Heather M. Pettis, Theoni Photopoulou, Stéphane Plourde, Nicholas R. Record, Jessica V. Redfern, Jason J. Roberts, Robert S. Schick, Burton Shank, Laura Solinger, Brandon L. Southall, Marisa L. Trego, Len Thomas

**Affiliations:** ^1^ Centre for Research into Ecological and Environmental Modelling, University of St Andrews St Andrews Scotland UK; ^2^ Sea Mammal Research Unit, School of Biology Scottish Oceans Institute, University of St Andrews St Andrews Scotland UK; ^3^ NOAA Fisheries Office of Protected Resources Silver Spring Maryland USA; ^4^ Biomathematics & Statistics Scotland, James Hutton Institute Aberdeen Scotland UK; ^5^ Department of Biology Sonoma State University Rohnert Park California USA; ^6^ Southall Environmental Associates, Inc. Aptos California USA; ^7^ Anderson Cabot Center for Ocean Life, New England Aquarium Boston Massachusetts USA; ^8^ Fisheries and Oceans Canada, Institut Maurice‐Lamontagne Mont‐Joli Québec Canada; ^9^ NOAA Fisheries Northeast Fisheries Science Center Woods Hole Massachusetts USA; ^10^ School of Earth, Ocean and Environment University of South Carolina Columbia South Carolina USA; ^11^ Marine Chemistry & Geochemistry Department Woods Hole Oceanographic Institution Woods Hole Massachusetts USA; ^12^ Biology Department Woods Hole Oceanographic Institution Woods Hole Massachusetts USA; ^13^ NOAA Fisheries Northeast Fisheries Science Center Narragansett Rhode Island USA; ^14^ Tandy Center for Ocean Forecasting Bigelow Laboratory for Ocean Sciences East Boothbay Maine USA; ^15^ Marine Geospatial Ecology Laboratory Duke University Durham North Carolina USA; ^16^ NOAA Fisheries Greater Atlantic Regional Fisheries Office Gloucester Massachusetts USA

**Keywords:** Bayesian state‐space modeling, cumulative risk, entanglements, *Eubalaena glacialis*, North Atlantic right whale, population consequences of multiple stressors, prey availability, stressor interactions, vessel strikes

## Abstract

Exploring solutions to expanding industrial activities and climate change requires assessments of the combined effects of multiple stressors on wildlife populations. We present a spatially explicit state‐space model for the health, survival, reproduction, and somatic growth of individuals in a long‐lived, wide‐ranging species. The model is applied to critically endangered North Atlantic right whales (*Eubalaena glacialis*) to investigate the combined effects of three primary stressors affecting the species' viability: entanglements in fishing gear, vessel strikes, and prey availability. We estimate exposure to these stressors in space and time and assess how their effects may combine in the pathway from exposure to vital rates. Results suggest that changes in whale distribution after 2010 led to increased entanglement risk. Poorer prey conditions were associated with an increased effect of carrying fishing gear, but, overall, results on combined effects were not conclusive and depended on model formulation. We also incorporated the estimated effects of stressors into a population viability analysis to explore alternative scenarios of stressor reduction. This integrated analysis highlighted the importance of the declining trend in maximum body length and its effect on reproduction, in addition to the documented impact of entanglements on survival. Model development and application elucidated critical data needs and the influence of underlying mechanistic assumptions. Specifically, models for the combined effects of stressors hinge on the availability of extended longitudinal measurements of individual health and life history outcomes, extensive datasets on the spatiotemporal distribution of stressors, and information on individual space use affecting rates of exposure to stressors. Lessons from this data‐rich case study will support the generalization of the modeling approach to other long‐lived species where measuring the population‐level consequences of multiple stressors directly is unfeasible.

## INTRODUCTION

Expanding urbanization, industrial development, and extraction of resources from the environment are exerting increasing pressure on wildlife populations and their ecosystems (Steffen et al., 2011), exacerbated by global climate change. Managing the combined effects of multiple stressors resulting from these activities in variable environmental contexts is one of the greatest challenges conservation science will face in the coming decades (Rudd, 2014; Steffen et al., 2011). Tyack et al. (2022) argued that environmental regulatory frameworks and management approaches should move away from focusing on the incremental effect of specific proposed actions when added to the existing set of actions and toward assessing cumulative risk to populations of interest. Ultimately, the goal of management frameworks should be to identify combinations of stressors that can be feasibly reduced to ensure that conservation targets are met (Tyack et al., 2022).

Devising management solutions that can reduce risk to a population below an acceptable level requires quantification of the combined effects of multiple stressors on individuals and the population. Pirotta et al. (2022) discussed a range of analytical approaches that have been used to assess combined effects across disciplines, spanning from empirical to mechanistic, and showed that the choice depends on both the availability of data on the effects of combinations of stressors and management requirements. Data availability is likely to remain a challenge for populations of long‐lived species that range widely, especially in the marine environment where data collection is logistically complicated and expensive, and many species regularly cross international boundaries. For these species, National Academies (2017) proposed a conceptual approach to understand the population consequences of multiple stressors (PCoMS), which outlines the mechanisms that link the exposure to stressors to the longer term effects on individual vital rates and population dynamics. This guiding approach is centered on the concept of individual health, which comprises a set of indicators (e.g., energy reserves, endocrine status, and immune status) that characterize “the ability of an organism to adapt to and manage threats to survival and reproduction” (Tyack et al., 2022). In practice, the PCoMS approach has often been operationalized using agent‐based modeling (Grimm & Railsback, 2013), which mechanistically simulates individuals moving in space and interacting with spatially explicit stressor agents or surfaces. A more phenomenological implementation involves the use of state‐space models (Auger‐Méthé et al., 2021), where the underlying health of an individual is a latent state integrating the effects of stressors and determining its survival and reproduction, and which can be observed through diverse data streams.

These complex, data‐hungry models can be developed in species where long‐term datasets exist, the most impactful stressors to the animals are known, and the underpinning ecological mechanisms are better understood (Pirotta et al., 2018). The lessons learned from these applications inform critical data needs and highlight analytical challenges, which will ultimately support the generalization to other species, including those that are less well studied (National Academies, 2017). Among marine mammals, critically endangered North Atlantic right whales *Eubalaena glacialis* (hereafter NARW) are a useful case study for the development of state‐space modeling approaches, given the breadth of available long‐term data on individuals (Moore et al., 2021; Pirotta et al., 2023; Schick et al., 2013). This species is threatened by many stressors, including entanglements in fishing gear, vessel strikes, and climate‐driven changes in prey availability, among others, which likely interact in complex ways. Vessel strikes and entanglements have both lethal and sublethal effects (Knowlton et al., 2012, 2022; Sharp et al., 2019; van der Hoop et al., 2017). Prey availability has also been linked to reduced calving rates (Meyer‐Gutbrod et al., 2021), as well as a spatial redistribution that altered the exposure to other stressors and caused a spike in mortality (Meyer‐Gutbrod et al., 2023). Likely as a result of combined stressors, the mean asymptotic body length across individuals has been decreasing (Stewart et al., 2021), with consequences on female reproductive output (Pirotta et al., 2024). Building on the work by Schick et al. (2013), Pirotta et al. (2023, 2024) developed a state‐space model for the health, survival, calving and somatic growth of individual NARW. This model estimated the separate effects of stressors but ignored the spatiotemporal variation in stressor exposure and did not explicitly test for any combined effects. However, when occurring in combination, stressors may operate differently than in isolation and lead to unexpected outcomes (Orr et al., 2020; Pirotta et al., 2022). Ultimately, assessing the relative contributions of stressors, operating in isolation or in combination, to individual health and vital rates can inform population viability analyses (PVA) used to evaluate the effectiveness of management measures. A NARW PVA tool has recently been published (Runge et al., 2023), which can incorporate estimated combined stressor effects to predict the population's trajectory under alternative scenarios of ecological and anthropogenic stressors.

We extend the Pirotta et al. (2023, 2024) model to explicitly estimate NARW spatial distribution and thus the variation in exposure to the stressors of interest in space and time. This extension involves the collation of data on the distribution and intensity of stressors over large spatial extents and long time periods. We then use this extended model to assess how stressor effects may combine at different levels in the pathway between exposure and population consequences. We further evaluate how this estimation is affected by model formulation and the assumptions imposed by data limitations. Finally, we investigate how the results of this retrospective analysis could be incorporated into the existing PVA tool to guide the evaluation of reducing the combined effects of stressors to promote the population's viability. Altogether, this extensive modeling effort demonstrates the challenges of quantifying cumulative risk on populations of long‐lived organisms. We highlight critical data needs and the influence of mechanistic assumptions underpinning such complex modeling exercises and provide guidance on how the results can be used to support conservation strategies. In doing so, we identify key insights that can guide similar endeavors in other systems.

## MATERIALS AND METHODS

### Existing model summary

This study builds on a Bayesian state‐space model for the survival and calving probability of NARW, which estimates each individual's underlying health status at a three‐month time scale (Pirotta et al., 2023). The model is informed by the 1970–2019 dataset provided by the NARW Consortium (NARWC; www.narwc.org/narwc-databases.html), comprising sightings of individual whales, information on their sex and age class, a qualitative assessment of their health from photographs, records of females with calves, documented deaths, and anthropogenic traumas. The effects of intrinsic (lactation status and the transition from calf to juvenile) and extrinsic stressors (entanglements, vessel strikes, and prey abundance) on the underlying health status are modeled. Recently, the model was extended to include a component for individual length, informed by photogrammetric measurements from occupied aircraft and drones and estimated to affect female calving probability (Pirotta et al., 2024). Full details of the data and of model formulation are provided in Pirotta et al. (2023, 2024), and a concise summary is provided in Appendix S1: Section S1.

### Spatial structure

**FIGURE 1:**
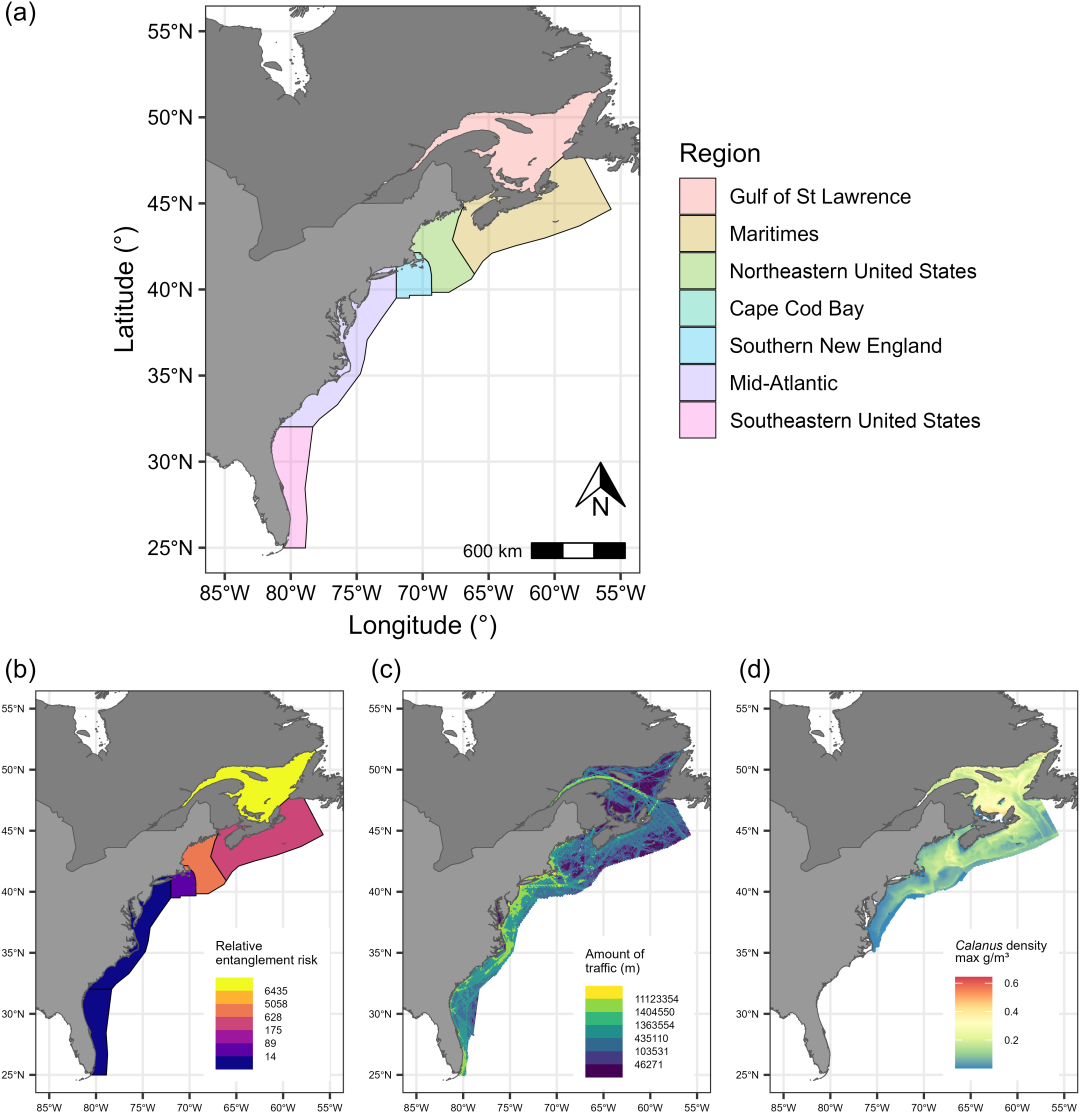
Map of the seven regional polygons used to model NARW spatial distribution and exposure to stressors (a), and examples of stressor data (b–d). In (b), relative entanglement risk derived from the WHALE DST for the Jun–Aug interval in the five US regions, extrapolated into Canadian regional polygons. In (c), total amount of vessel traffic in meters in June 2019, summarized over a 10 km × 10 km grid. In (d), maximum concentration (in grams per cubic meter) of copepods in the genus *Calanus* in June 2019 in the depth range 0–306 m, at a 0.083° spatial resolution, as predicted by the model in Plourde et al. (2024).

The models published in Pirotta et al. (2023, 2024) are non‐spatial, that is, the location of individuals and their resulting exposure to stressors were not modeled explicitly. Here, we extended the model to account for individual spatial distribution. Sighting data are too sparse to inform fine‐scale individual movements and space use. Therefore, we collapsed the population's range into seven regional polygons (following Schick et al. (2013); Figure 1a) and modeled an individual's proportional presence in those polygons in a three‐month time step. Individual sightings were collected by multiple survey programs; the associated survey effort has varied in space and time and has not been consistently reported for all datasets within the database. An existing density surface model for the species exists, but it only uses a subset of the data resulting from systematic effort (Roberts et al., 2024).

Therefore, we developed two versions of the spatial model: a data‐driven version that uses all reported sightings and ignores the problem of heterogeneous effort (v1); and an entirely model‐based version that builds on the densities predicted by Roberts et al. (2024) (v2). For the data‐driven version, we fitted a Dirichlet model for the proportion of time an individual within a given demographic class (calf, juvenile, adult male, or adult female) spent within each regional polygon in each season, estimating a separate set of parameters for the period prior to 2010 versus from 2010 onward to capture the shift in distribution observed after 2010 (Meyer‐Gutbrod et al., 2023) (Appendix S1: Section S2.1). For the model‐based version, we assumed that the relative whale abundance in each polygon over a three‐month interval reflects the distribution of the average individual, which was then rescaled using the proportional occurrence of different demographic classes per region and three‐month interval (Appendix S1: Section S2.2). Note that density predictions by Roberts et al. (2024) are available for the periods 2003–2009 and 2010–2019. We assumed that the distribution prior to 2003 was the same as in 2003–2009. Because Roberts et al. (2024) model is largely restricted to US waters, we used the ratio of the number of sightings to modeled abundance in the Northeastern US polygon to infer the abundance in the two Canadian regional polygons (Appendix S1: Section S2.2). The two alternative versions were used to investigate the influence of model formulation on the results.

### Spatially explicit stressor surfaces

Assessing spatially explicit exposure to stressors over time requires estimates of the spatiotemporal distribution of individual whales and of the different extrinsic stressors included in the model. Across stressors, deriving surfaces for the entire range of the population and study period required extensive assumptions and simplifications to fill current data gaps. These are discussed in Appendix S1: Sections S3–S5 and impose caution in the interpretation of emerging exposure patterns.

For entanglement risk, we used the baseline risk outputs from the Woods Hole Analysis of Line Entanglement Decision Support Tool (WHALE DST; Miller et al., 2025). Briefly, the WHALE DST estimates the relative risk posed by fixed‐gear commercial fisheries (trap/pot and gillnet) within Atlantic US waters (Miller et al., 2025), calculated as the product of the number of vertical fishing lines within a grid cell, the threat those lines pose to NARW given rope strength and gear configuration, and NARW abundance at that location from Roberts et al. (2024). WHALE DST risk estimates were averaged at the scale of our regional polygons and in each three‐month period, weighting by the relative whale density in each polygon (Figure 1b). The results were assumed to reflect recent entanglement risk, based on the determined range of years that best captured fixed‐gear fishing effort prior to the 2021 modification of the Atlantic Large Whale Take Reduction Plan (Miller et al., 2024, 2025). For previous years (1970–2014), we scaled baseline risk values using the trend in landings from fisheries that pose the greatest entanglement risk to NARW (Appendix S1: Section S3). As the WHALE DST only covers US waters, we estimated entanglement risk in Canadian regional polygons by scaling the total risk in northern US regions by the ratio between lobster and crab fisheries landings within each Canadian region and landings from those fisheries in northern US regions (Appendix S1: Section S3).

We derived vessel strike risk using vessel tracking data captured by Automatic Information Systems (AIS; available for vessels over 19.8 m in length, Appendix S1: Section S4) over the population's range in 2019, which were obtained from low‐orbiting satellite constellations (ORBCOMM, https://www.orbcomm.com/eu) and terrestrial stations (USCG Nationwide Automatic Identification System) for US waters, and from the MERIDIAN initiative at Dalhousie University (https://meridian.cs.dal.ca/) for Canadian waters. AIS data were summarized into total vessel transit distances (sensu Redfern et al., 2024, Figure 1c), weighted by the relative spatial distribution of NARW in each polygon (Roberts et al., 2024), and then averaged in each regional polygon and three‐month interval (details in Appendix S1: Section S4). The temporal trend in strike risk prior to 2019 was estimated within the model (see below).

Prey distribution and density were obtained from a model for three copepod species in the genus *Calanus* (*C. finmarchicus*, *C. hyperboreus* and *C. glacialis*), which are important prey of NARW (Appendix S1: Section S5). The model combined estimates of water‐column abundance, derived from zooplankton survey data in US and Canadian waters and modeled as a function of environmental variables, with predictions of copepod body size and vertical distribution (Plourde et al., 2024). It returns predicted 3D *Calanus* concentrations (in grams per cubic meter) between 1999 and 2019 (Plourde et al., 2024; Figure 1d). We assumed that in each spatial cell a whale would target the depth layer with maximum predicted concentration within the species' diving range (Baumgartner & Mate, 2005). We then computed the mean prey concentration in each regional polygon (weighted by whale relative density in the region) and three‐month interval. The resulting index of prey conditions was extrapolated for the period not covered by the *Calanus* model (1970–1998) by fitting a log‐linear regression model between the values of the index in 1999–2019 and the time series of annual anomalies of late‐stage *C. finmarchicus* abundance from the Continuous Plankton Recorder data in the Gulf of Maine (Pershing et al., 2005; Appendix S1: Section S5).

### Modifications to the NARW model to incorporate spatially explicit stressors

The probability of entanglement and vessel strike in each three‐month time step was modeled as the combination of an individual's distribution in that interval and the values of the corresponding risk surfaces in the regions used by the individual, rescaled to lie between 0 and 1. Specifically, entanglement probability for individual *i* at time *t* was

(1) $$p_{i,t}^{e}=\iota_{1}\sum_{l=1}^{R}z_{t,i,l}G_{l,t}\;{\tilde{e}}_{l,t},$$

where *R* = 7 is the number of regional polygons, *z*_*t,i,l*_ is the proportion of time step *t* an individual *i* spent in polygon *l*, *G*_*l,t*_ is the value of the entanglement risk surface for that polygon *l* and time step *t*, ${\tilde{e}}_{l,t}$ is the scalar derived from the landings data to extrapolate risk to years prior to 2015, and $\iota_{1}$ is an estimated parameter (with a constrained prior; Appendix S1: Section S10) that converts the relative risk value into a probability of getting entangled in that time step.

For vessel strike probability, we first modeled strike probability in each region and time step as

(2) $$\begin{array}{c} \text{logit}\left(p_{l,t}^{r}\right)=\text{logit}\left(\iota_{2}\;{\mathrm{AIS}}_{l,t}\right)+\tilde{v}\;\left(2019-y_{t}\right), \end{array}$$

where AIS_*l,t*_ is the value of the vessel strike risk surface for that regional polygon and time step, $\iota_{2}$ is an estimated coefficient (with a constrained prior; Appendix S1: Section S10) used to rescale the risk into a probability, $\tilde{v}$ is the estimated temporal trend in strike risk (meant to capture changes in vessel traffic over time), and *y*_*t*_ is the corresponding year. This formulation ensures that strike probability is directly proportional to the AIS‐derived risk in 2019, and changes linearly (on the logit scale) going back in time. These probabilities were then multiplied by the individual's spatial distribution to compute the overall strike probability for that individual in a time step:

(3) $$\begin{array}{c} p_{i,t}^{v}=\sum_{l=1}^{R}z_{t,i,l}\;p_{l,t}^{r}. \end{array}$$

We used entanglement and strike probabilities in submodels to determine an individual's entanglement state (*E*_*i,t*_) and vessel strike state (*V*_*i,t*_), that is, the occurrence of observed entanglement and vessel strike events, as well as one potential unobserved entanglement and one potential unobserved vessel strike at the end of an individual's time series (i.e., after its last sighting) to capture cryptic deaths (Pace et al., 2021) (Appendix S1: Sections S6 and S7). The effect of an event on individual health (given its severity, for entanglements, or the type of injury, for vessel strikes) was then estimated as described in Pirotta et al. (2023). Severity and type of injury were known for observed events, or estimated for unobserved events (Appendix S1: Sections S6 and S7). In light of the partial confounding between the immediate and prolonged effects of entanglements highlighted in Pirotta et al. (2023), we modified their formulation so that the prolonged effect of an entanglement event did not apply in the time step when the event occurred; therefore, the effect in the first time step included both the initial effect on health and the effect of carrying the gear for that interval (Appendix S1: Section S6).

The index of prey conditions in each region and time step (${\text{prey}}_{l,t}$) was first weighted by an individual's distribution:

(4) $$\begin{array}{c} {\mathrm{prey}}_{i,t}^{\prime }=\sum_{l=1}^{R}z_{t,i,l}\;{\mathrm{prey}}_{l,t}. \end{array}$$

We then computed the mean of the prey conditions experienced by an individual in a given year, standardized it using the mean and SD across all annual means, and estimated its effect on health in the June–August interval as in Pirotta et al. (2023) (Appendix S1: Section S5).

### Mechanisms for combined effects of stressors

There are several pathways through which stressors may combine to affect an individual's health and vital rates (Ankley et al., 2010; Pirotta et al., 2022). We formulated a set of a priori hypotheses for the potential combined effects of the stressors included in our model, which were then tested explicitly (Table 1). Stressors may combine at the level of individual health, for example, if the effects of traumatic events vary depending on prey conditions, or if the effect of an entanglement differs when the individual was previously entangled. While we explicitly tested for interactions between pairs of stressors in the process model for health, it should be noted that, due to the nonlinear relationship between health and survival, the combined effect of two stressors on survival was intrinsically different from the sum of the effects of those stressors operating in isolation (Pirotta et al., 2022).

**TABLE 1:** Hypothesized mechanisms through which stressors may combine to affect North Atlantic right whales (NARW) health, length, or exposure.

Effect level	Combined effect	Hypothesized mechanism
Health	Prey × entanglement (prolonged)	Poor prey conditions may worsen the effect of carrying fishing gear
	Prey × entanglement (immediate)	Poor prey conditions may worsen the initial effect of an entanglement (by severity)
	Prey × vessel strike	Poor prey conditions may worsen the effect of a vessel strike (by injury type)
	Multiple entanglements (cumulative)	The total no. prior entanglements may worsen the effect of a subsequent one
	Multiple entanglements (occurrence)	The occurrence of prior entanglements may worsen the effect of a subsequent one
	Multiple entanglements (occurrence in previous 2 years)	The occurrence of prior, recent entanglements may worsen the effect of a subsequent one
Length	Prolonged health (first 15, 10, 5, 2, or 1 year)	A prolonged period of poor health may impact an individual's growth
	Mother's health (start of lactation)	Poor health of the mother at the start of lactation may affect a calf's growth
	Mother's health (lactation year)	Poor health of the mother during the year of lactation may affect a calf's growth
	Prey × entanglement (mean in first 15, 10, 5, 2, or 1 year)	Low mean prey conditions and high mean entanglement status may impact somatic growth
	Mother's entanglement status	If the mother was entangled during the year of lactation, this may affect a calf's growth
Exposure	Change in distribution × entanglement risk	A change in spatial distribution may alter the exposure to entanglement risk
	Change in distribution × vessel strike risk	A change in spatial distribution may alter the exposure to vessel strike risk
	Prey × entanglement risk	Different prey conditions may alter the probability of getting entangled
	Prey × vessel strike risk	Different prey conditions may alter the probability of getting struck

**FIGURE 2:**
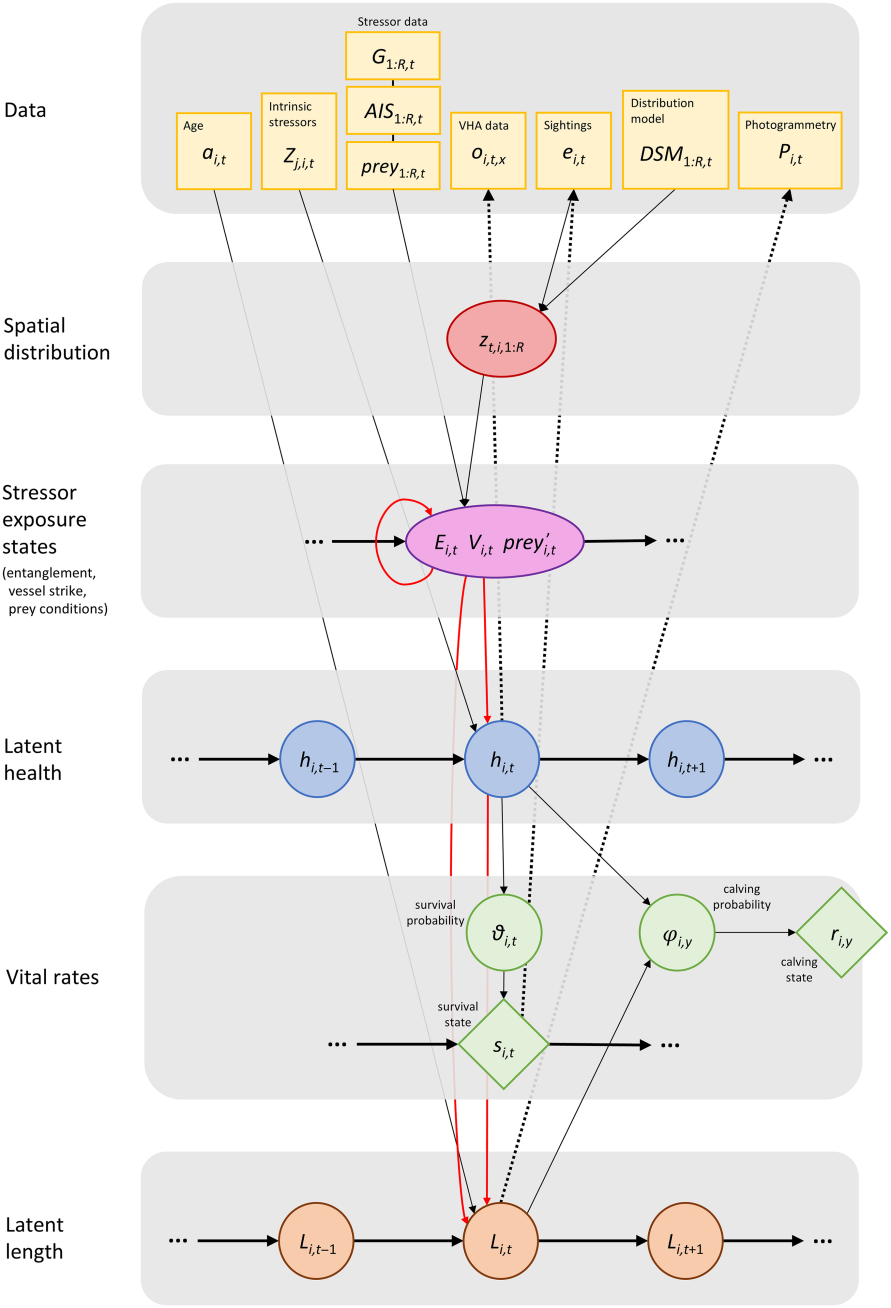
Schematic representation of the spatial version of the model. Model processes are represented for individual *i* at time *t*. The yellow boxes indicate the data streams, including age informing the somatic growth model (*a*_*i,t*_), intrinsic stressors (*Z*_*j,i,t*_) affecting health, the values of the stressor surfaces across the *R* regional polygons (*G*_1:*R*,*t*_ representing entanglement risk, ${\mathrm{AIS}}_{1:R,t}$ representing vessel strike risk, and prey_1:*R*,*t*_ representing prey conditions), NARW density derived from Roberts et al. (2024) density surface models (DSM_1:*R*,*t*_), and the observation models (VHA data *o*_*i*,*t*,*x*_, where *x* indicates each of the four VHA variables, sightings *e*_*i*,*t*_, and photogrammetric measures of length *P*_*i*,*t*_; dotted arrows). The proportion of a time step an individual spends in different regions (*z*_*t*,*i*,1:*R*_; red circle) is estimated using density surface model predictions (DSM_1:*R*,*t*_) and/or individual sightings *e*_*i,t*_, and, combined with the three stressor surfaces, determines an individual's stressor exposure states (purple circle, i.e., whether it is entangled, *E*_*i,t*_, or vessel struck, *V*_*i,t*_, and the mean prey conditions it experiences, ${\text{prey}}_{i,t}^{\prime }$; in the model, the annual mean prey conditions affect health in the Jun–Aug interval, but here, we represent prey conditions experienced in each time step, for simplicity). The blue circles represent the time series of latent health, *h*_*i,t*_. Health affects survival probability ϑ_*i,t*_ in time step *t* and calving probability φ_*i*,*y*_ in available years *y* (green circles), which determine the time series of the two vital rates (survival *s*_*i,t*_ and calving *r*_*i,y*_, green diamonds). The orange circles represent the time series of latent length (*L*_*i,t*_), which depends on age (*a*_*i,t*_) and affects calving probability in females. The solid red arrows indicate the combined effects of stressors we investigated (Table 1). For more details on model formulation and notation, please refer to Appendix S1: Sections S1 and S9.

Combined stressors not only affect health; if somatic growth rates are affected, individual asymptotic length (the maximum length to which a whale will grow) may also vary. Therefore, we investigated the effect of prolonged health status and the direct effects of stressors on an individual's asymptotic length. Prolonged health was defined as the mean health over a variable window covering the first years of an individual's life (Table 1). Finally, we investigated the change in exposure to entanglement or vessel strike risk that may result from changes in whale distribution or prey conditions. The updated model structure is summarized in Figure 2.

The combined effects of stressors were modeled across the three available model formulations (i.e., the two spatial versions described above, v1 and v2, and the non‐spatial version described in Pirotta et al. (2024), v3), except for the combined effects on stressor exposure resulting from the change in distribution, which were only considered for the two spatial formulations, and the effects of prolonged health on length, which were only tested in the non‐spatial version. The details of the implementation of these effects in the model are reported in Appendix S1: Section S8. The estimated effects are discussed in terms of the sign of the corresponding parameter(s) and the overlap of their posterior 95% credible intervals with 0 (except for the combined effects on exposure, which are discussed in terms of the associated probabilities of entanglement and vessel strike).

### Bayesian inference

The models were fitted in a Bayesian framework using Markov chain Monte Carlo (MCMC) algorithms implemented in software JAGS ver. 4.3.0, run through package runjags (Denwood, 2016) for R (www.r-project.org). Details of model setup, convergence, and fitting diagnostics are described in Pirotta et al. (2023, 2024). The prior distributions and constraints of model parameters are listed in Appendix S1: Section S10. Results are discussed in terms of the 95% credible interval of a subset of parameters of interest, while other posterior estimates are reported in Appendix S1: Section S10.

### Using model results in the NARW PVA tool

To demonstrate the potential application of our results to assess alternative stressor scenarios, we integrated them into an existing PVA tool for NARW (Runge et al., 2023). To facilitate this incorporation, we modified our model to formulate survival in terms of hazard rates, as per Runge et al. (2023) (Appendix S1: Section S14). The PVA code was modified to: (1) incorporate the distribution of the health of uninjured animals and the estimated hazard ratios of the stressors; (2) model calving probability as a function of health; (3) model somatic growth as change in length and its effect on calving probability; and (4) build in the autocorrelation in health and survival between subsequent years (Appendix S1: Section S14). We selected one version of our model (with data‐driven spatial structure, v1, and including the combined effects of prey conditions and the prolonged effect of entanglements) for illustration, and used the PVA to simulate the population's trajectory in the next 100 years under a set of scenarios. Because this exercise was meant for demonstration, we did not consider changes in vessel strike risk, and only investigated: combinations of entanglement risk reduced by 0%, 50%, and 100%; prey conditions remaining low, as in the decade 2010–2019, or returning to the historical variation observed in 1990–2009; NARW asymptotic length continuing to decline at the current rate (down to a minimum of 10 m), stabilizing at the current mean (~11.8 m), or reverting its trend up to the historical maximum (~14.0 m). As in the original PVA (Runge et al., 2023), we propagated the uncertainty in model estimates by rerunning the projections using 1000 samples from the input distributions. There are some important differences underpinning the estimation of NARW vital rates and stressor effects between our modeling approach and the PVA (Appendix S1: Section S14). Therefore, this exercise is intended for demonstration only, and results of the PVA should be interpreted with caution and not used to inform management decisions.

## RESULTS

NARW spatial distribution showed some substantial differences between the two spatial formulations of the model, v1 and v2 (Appendix S1: Figure S1), likely due to heterogeneous survey effort in different regions and periods. For example, the model based on NARWC sightings (v1) suggested a greater occurrence in the Southeastern United States for adult females and juveniles during Dec–Feb, but lower occurrence in the Mid‐Atlantic during the same interval. Model v1 also highlighted a larger presence in Cape Cod Bay during Mar–May from 2010 onward. In contrast, occurrence in the Northeastern United States was higher across demographic classes during Jun–Aug in the model based on the density surfaces (v2), which also captured an increased use of Southern New England and the Mid‐Atlantic from 2010 onward, particularly during Sep–Nov. Both versions of the model highlighted increased use of the Gulf of St Lawrence during Jun–Aug from 2010 onward. In general, model v2 resulted in the distribution of individual whales being more evenly spread across the range from 2010 onward, particularly during Jun–Aug and Sep–Nov, which reflects the larger uncertainty associated with the predicted densities in this period.

**FIGURE 3:**
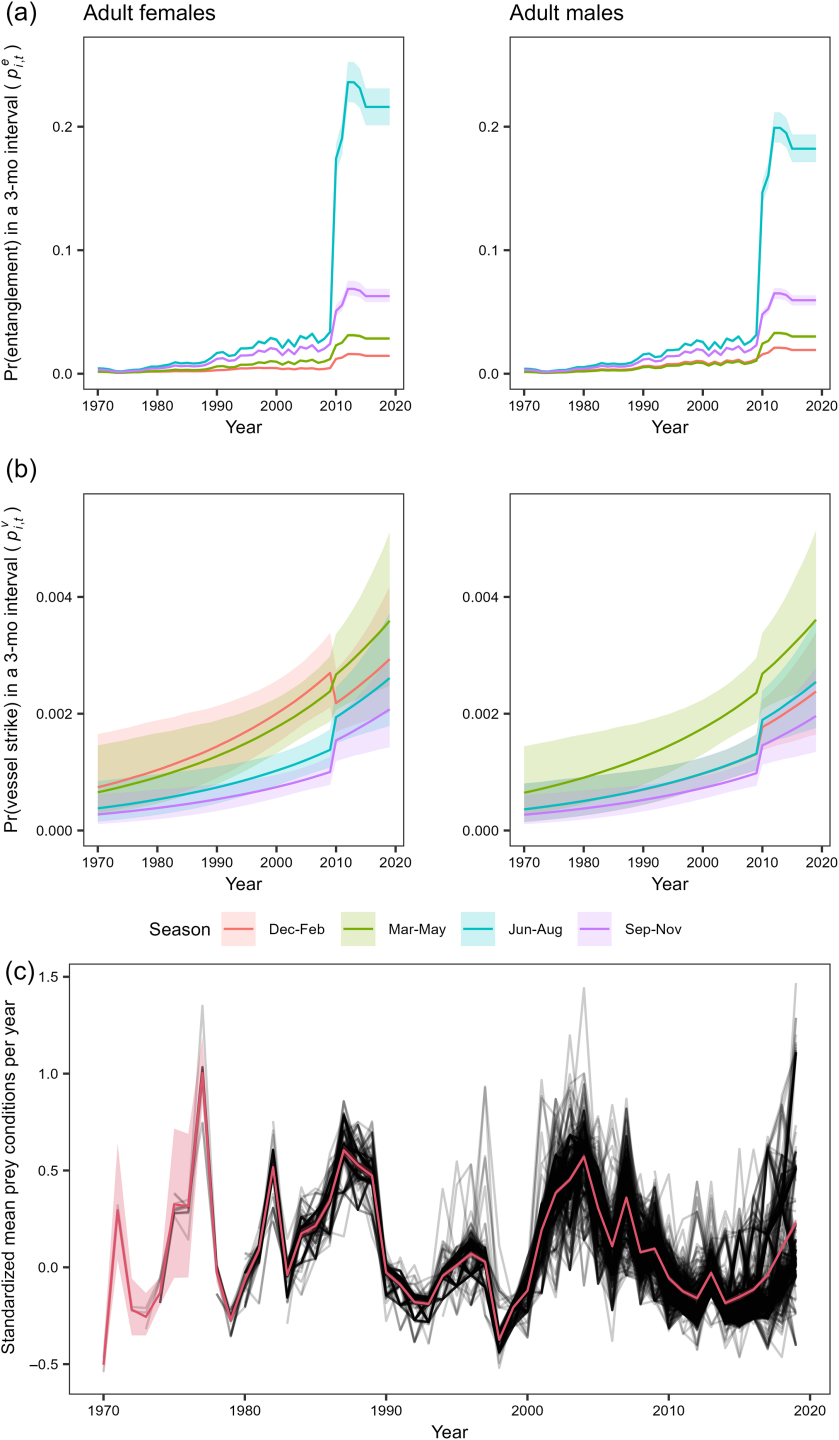
Estimated stressors over time, summarized across regions. Several assumptions underpin the extrapolation of stressors in space and time, which warrant caution in the interpretation of these patterns. In (a) and (b), probability of entanglement ($p_{i,t}^{e}$) and vessel strike ($p_{i,t}^{v}$) in each three‐month interval (indicated by the corresponding start and end months) for adult females and adult males, calculated using the average spatial distribution from model v1. In (c), standardized mean annual prey conditions (unitless), calculated from the mean conditions in each interval ${\mathrm{prey}}_{i,t}^{\prime }$ (from model v1). Variation in stressor exposure across intervals is driven by seasonal variation in both whale distribution and stressor intensity (see Appendix S1: Sections S2–S5). The colored lines and ribbons indicate the posterior medians and 95% credible intervals (note that in (c) the red credible interval gets too narrow to be visible after ~1980). Each black line in the bottom row corresponds to the median for each individual.

**FIGURE 4:**
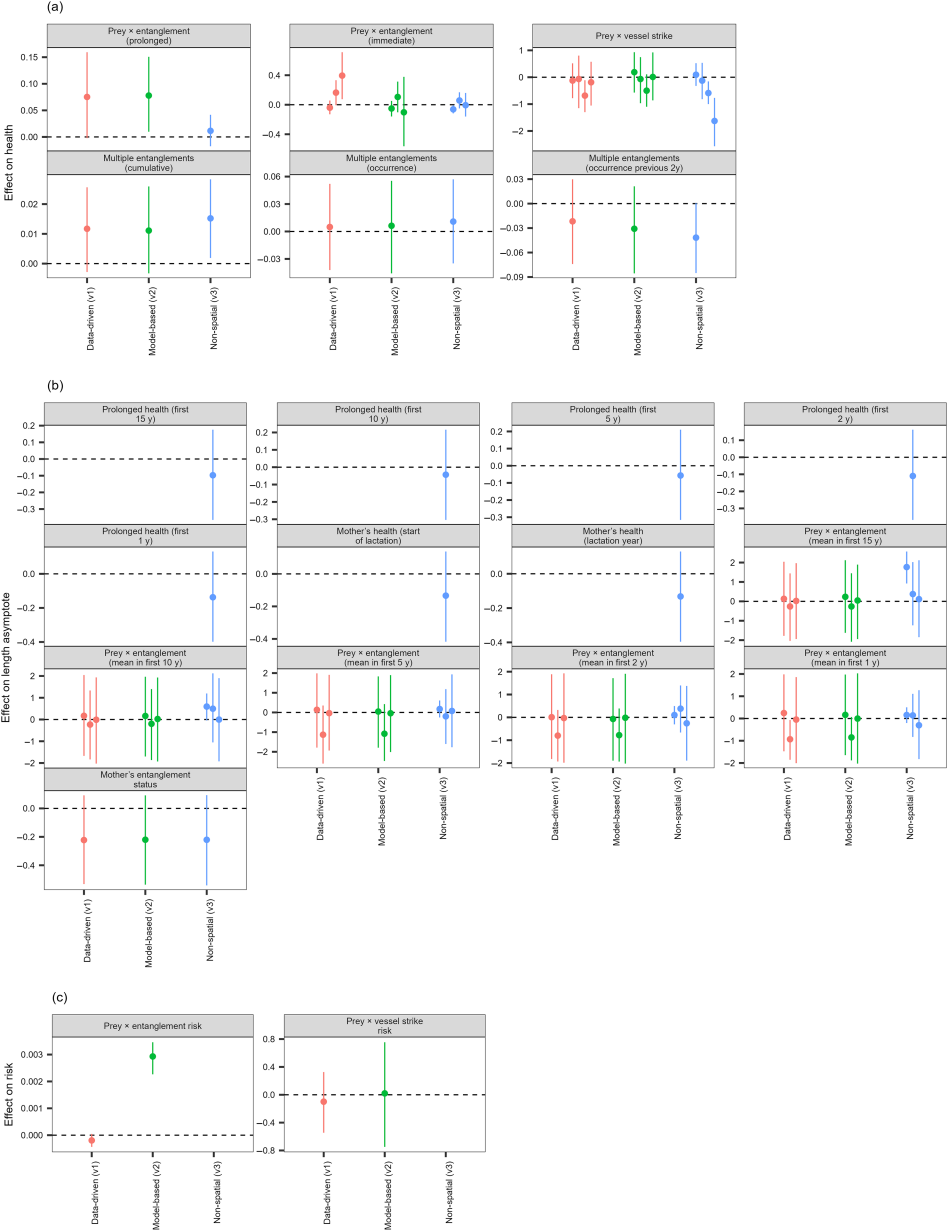
Combined effects of stressors on health (a), length (b), and exposure risk (c), as described in Table 1, across the three model formulations (x axis and colors). The points represent the posterior medians of the associated parameter(s), while the segments are the 95% credible intervals. A positive effect indicates an improvement in health status (a), a larger asymptotic length (b), or a greater exposure risk (c). Note that the immediate effect of an entanglement differs by severity (three levels, from left to right: minor, moderate, severe), while the effect of a vessel strike is by injury type (four levels, from left to right: superficial, shallow, deep, blunt). The three parameters in some of the models for the length asymptote (b) correspond to the effects of prey, entanglement and their interaction, respectively.

Variation in estimated spatial distribution drove differences in stressor exposure between the two models (Figure 3 and Appendix S1: Figures S8–S17). However, both models highlighted an increase in entanglement probability (Figure 3), with particularly high levels in the Gulf of St Lawrence (Appendix S1: Figures S8 and S13), from 2010 onward, following a shift in NARW distribution (Appendix S1: Figure S1). The trend in landings from fisheries with high entanglement rates also led to increased entanglement probability in other northern regions prior to 2010, and much lower probabilities in southern regions (Appendix S1: Figures S8 and S13). Vessel strike probability was estimated to have increased over time in both models v1 and v2 (Figure 3). However, strike probability by region and season varied substantially between models v1 and v2, driven by the differences in spatial distribution (Appendix S1: Figures S10 and S15), with the partial exception of consistently high levels in the Southeastern United States during Dec–Feb for adult females, in Northeastern United States during Mar–May for both males and females, and, to a lesser extent, the Maritimes during Sep–Nov. Strike probability increased in the Gulf of St Lawrence during Jun–Aug in both models, but levels remained lower than in other regions (Appendix S1: Figures S10 and S15). The mean prey conditions experienced by individuals across years were consistent across the two formulations (Appendix S1: Figures S12 and S17), but the Dirichlet spatial model in model v1 led to greater interindividual variation (Figure 3). We found no evidence that average prey conditions in a year resulted in increased strike risk, while the effect on entanglement risk was small and inconsistent across models v1 and v2 (Figure 4 and Appendix S1: Table S5).

The combined effects of stressors on health (summarized in Figure 4 and Appendix S1: Table S5) showed some inconsistencies across the three model formulations. The immediate reduction in health resulting from severe and moderate entanglements was worsened during poor prey conditions in model v1, but the combined effect of poor prey with severe entanglements was not evident in models v2 and v3 (Figure 4). In contrast, the prolonged effect of carrying the gear on health was consistently larger with poorer prey conditions across the three formulations, although the overlap of the parameter's posterior distribution with 0 varied (Figure 4; Appendix S1: Figure S19, where the combined effect is exemplified for model v1). Some of the estimated combined effects were in the opposite direction to expectations: for example, good prey conditions appeared to be associated with a greater effect of vessel strikes causing deep wounds, and the cumulative number of prior entanglements was estimated to have a small positive effect on a subsequent event. Finally, entanglements that occurred within 2 years of a previous entanglement resulted in poorer health, although the posterior 95% CI showed some overlap with 0 in both spatial formulations (Figure 4 and Appendix S1: Table S5).

We found no evidence that the prolonged health status of an individual during somatic growth years, or of the mother at the start or during lactation, was associated with decreased asymptotic length (Figure 4 and Appendix S1: Table S5). There was also no evidence of combined effects of prey and entanglement status on this asymptote, but, in the spatial versions (v1 and v2), we found some indication that the mean amount of time spent in an entangled state in the first 1, 2, and 5 years of life resulted in stunted somatic growth (see models including the interaction between prey and entanglement in Figure 4b). This effect was not present in the non‐spatial version v3, where, instead, we found a correlation between asymptotic length and the mean prey index over the first 15 years and, to a lesser extent, 10 years of an individual's life (Figure 4b). Finally, we found some indication that the entanglement status of the mother was correlated with reduced asymptotic length of the calf, although the 95% CI showed some overlap with 0 across formulations (Figure 4).

**FIGURE 5:**
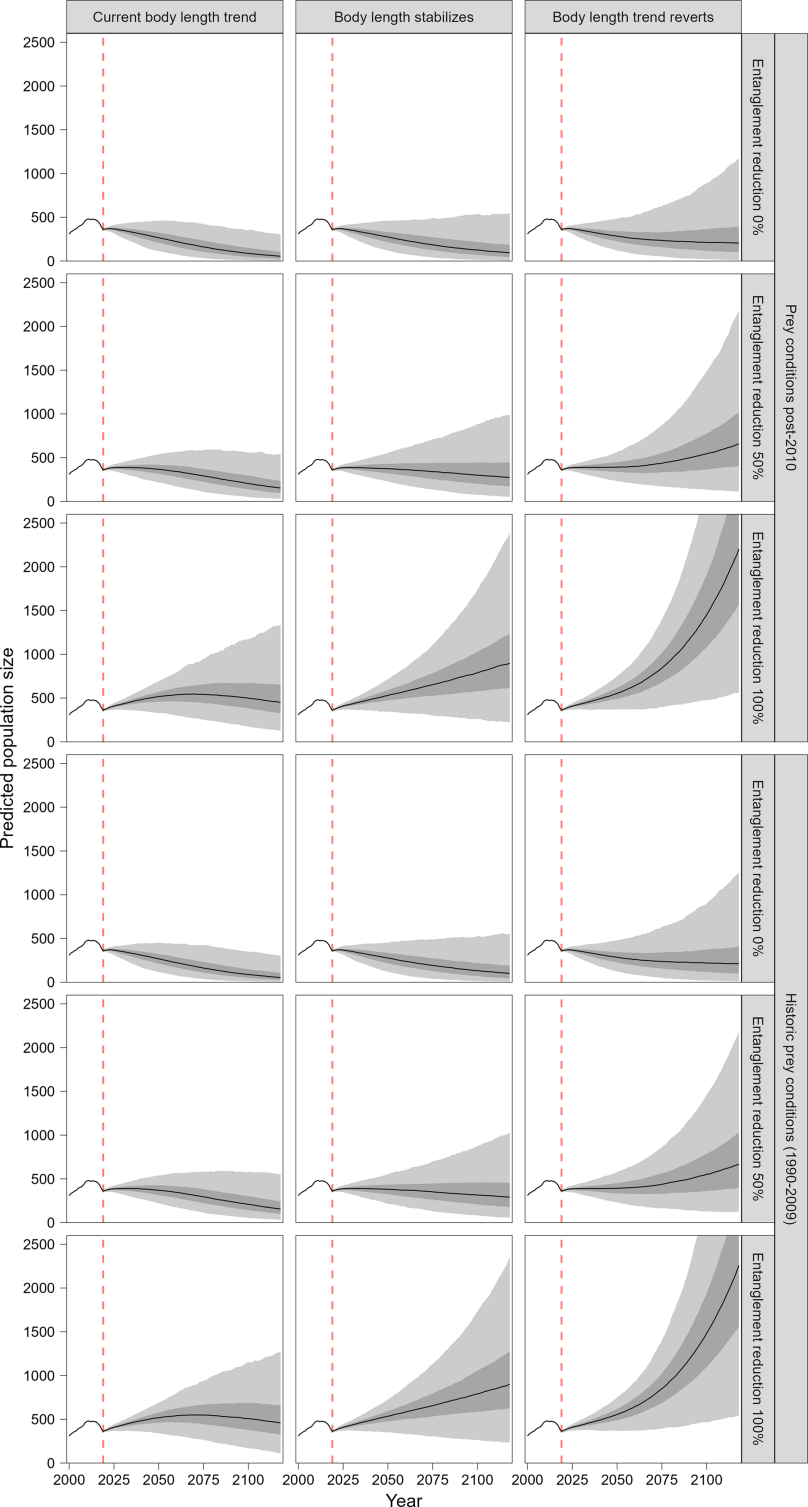
Historical and projected total NARW population size 2000–2119, under various scenarios of severe entanglement risk reduction (0%, 50%, or 100%), prey conditions (following historical patterns from 1990 to 2009, or reduced as in the post‐2010 period), and asymptotic body length (trend continuing along the current downward trajectory, stabilizing, or reverting toward the historical maximum length). The period before 2019 (vertical dashed line) shows the historical estimates of NARW population size; the period after 2019 shows the projections from the population viability analysis tool, modified to incorporate the results of our Bayesian state‐space model. The bold line shows the median value, the light gray shaded area encompasses the 2.5% and 97.5% quantiles (95% projection interval), and the dark gray shaded area encompasses the 25% and 75% quantiles (50% projection interval). Due to the large number of assumptions, these results are only demonstrative and should not be used to inform management.

The incorporation of our results in the PVA tool indicated that substantial reductions in entanglement risk are required to ensure the viability of the species (Figure 5; Appendix S1: Figure S22). Simulations also showed that, if the current declining trend in asymptotic length were to continue, the resulting calving probability may be insufficient to support a positive population growth rate even if entanglements were completely removed. In contrast, a stabilization or an inversion of that trend meshed with reductions in entanglements would substantially improve the chances of the population recovering and surviving. Prey conditions explored in these simulations did not appear to have any visible influence on the population's trajectory.

## DISCUSSION

**FIGURE 6:**
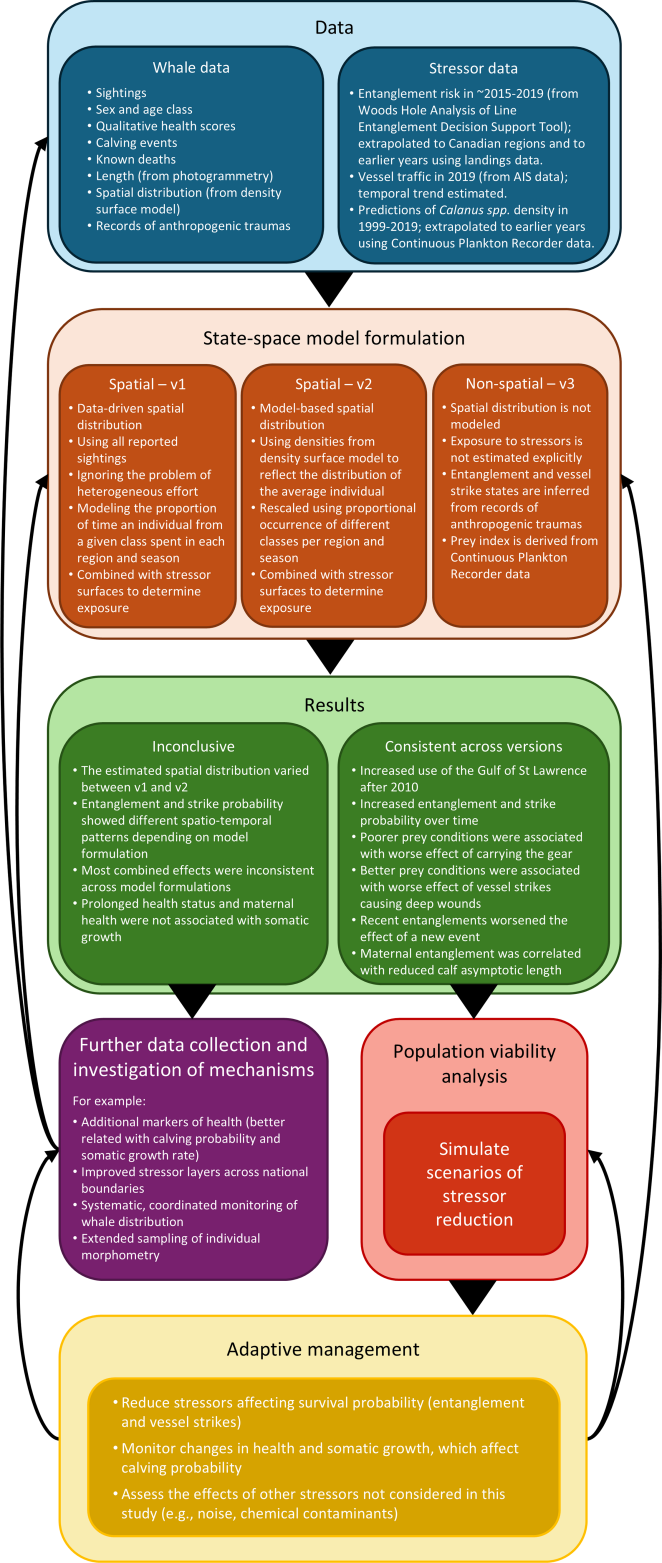
Conceptual diagram summarizing the workflow of this paper.

In this study, we present a spatially explicit state‐space model for the health, survival, reproduction and somatic growth of individuals in a long‐lived, wide‐ranging species, which can be used to assess the combined effects of multiple stressors resulting from human activities and environmental change. To illustrate its development and utility, we applied the model to critically endangered NARW to investigate how three of the main stressors that threaten population viability (entanglements, vessel strikes, and prey availability) may combine. These combined effects can manifest at different levels in the pathway between exposure and population dynamics (Pirotta et al., 2022; Tyack et al., 2022); here, we showed how the PCoMS framework can guide their explicit evaluation at the appropriate level. Ultimately, estimating combined effects within an integrated framework could help guide the evaluation of alternative scenarios where specific combinations of stressors are reduced to bring the cumulative risk to the population below some acceptable target; here, we demonstrated how this could be achieved in principle using our results within an existing predictive tool. Overall, this work represents a proof of concept for the development of an approach (summarized in Figure 6) that integrates diverse modeling efforts to assess the consequences of multiple stressors in a species where population‐level effects cannot be quickly detected but management needs are pressing (Taylor et al., 2007). Its application to a case study of high conservation concern provided important insights into the associated challenges and data gaps, highlighting the many uncertainties and assumptions involved in the integration of data and models.

### The combined effects of stressors on NARW

Both spatial model formulations describe the substantial changes in NARW distribution from 2010 onward (Appendix S1: Figure S1; Meyer‐Gutbrod et al., 2023). The increased use of the Gulf of St Lawrence was associated with an increased exposure to entanglement risk in this region (Appendix S1: Figures S8 and S13), although this may partly depend on our risk extrapolation into Canadian regions. Increased entanglement risk has been previously proposed as one of the drivers for an ongoing Unusual Mortality Event (Davies & Brillant, 2019). This change in exposure represents an example of a combined effect between prey availability (thought to underpin the change in distribution; Record et al., 2019, Meyer‐Gutbrod et al., 2023) and entanglements. The assumptions around how we modeled NARW spatial distribution, the trends in stressors over space and time, and the extrapolation of entanglement risk into Canada prompt caution in further interpretation of the spatiotemporal patterns of exposure.

Another indication of the combined effects of prey and entanglements is at the level of individual health, where poorer prey conditions were associated with an increase in the prolonged effect of entanglements (Appendix S1: Figure S19), albeit with varying support across formulations (Figure 4). This result aligns with our a priori hypothesis, since carrying fishing gear causes additional drag (van der Hoop et al., 2017) and the associated energetic cost may become particularly problematic when prey conditions are poorer. In poor prey years, gear may also limit the ability of animals to feed efficiently on low‐density prey patches, or move in search of better ones (Hütt et al., 2023). Separately, we modeled the effect of an entanglement injury at the time the gear was acquired, and this effect was not consistently associated with prey conditions (Figure 4), which suggests that the impact of an injury is not clearly remediated by better feeding opportunities. However, there was some indication that having recently been entangled might worsen the immediate effect of a subsequent entanglement (Figure 4).

We did not identify clear drivers of the documented decrease in NARW body length (Figure 4). Depending on model formulation, we found some indication that the entanglement status of a calf during the first years of life or the entanglement status of the mother when nursing had an effect on calf growth, in line with Stewart et al. (2021). In contrast, prey conditions only appeared to matter when summarized over a long temporal window in the non‐spatial model (Figure 4). The lack of association between average health status during growth years and decreasing asymptotic length may suggest that our health metric has limited ability to represent sublethal variations in individual health that underpin long‐term somatic growth patterns. Similarly, Pirotta et al. (2024) highlighted how the health metric captures only part of the variation in calving probability. Not investing energy in somatic growth may also be a strategy for an individual to maintain good health when conditions are poor.

Vessel strike risk was estimated to have increased over time across the entire range, as suggested by Vanderlaan et al. (2009) (Figure 3). However, the combined effects of vessel strikes with prey conditions were less clear (Figure 4), indicating that, similar to entanglement injuries, the impact of injuries from vessel strikes on survival probability may not be ameliorated by better prey conditions. We also note the unexpected result that better prey conditions were associated with a worse effect of a vessel strike causing a deep wound (Figure 4). While this could be a spurious result, its consistency across model formulations prompts some future investigations of the potential mechanisms. For example, this could reflect the limited scope for a further health decrease in poor prey years, or the prioritization of bioenergetically costly foraging effort at the expense of injury healing in good prey years.

Our model for the occurrence of traumatic events improves upon the previous, non‐spatial version in Pirotta et al. (2023) by modeling the time of occurrence and linking events with the underlying risk. The approach we propose here also aims to address the known issue of cryptic deaths after an individual's last sighting (Pace et al., 2021). However, the nonlinear nature of the function linking health and survival probability may lead to unobserved events being assigned to animals already in poorer health. Moreover, the model does not account for other cryptic events earlier in an individual's life, particularly those causing sublethal, undetected, blunt injuries. An individual's time of death after its last sighting might also be misrepresented given the simplistic way in which we model encounter probability, which is known to vary in space and time. In general, appropriately modeling the occurrence of a traumatic event given an underlying risk surface, the acquisition of an injury of a given severity, the recovery or death that ensues, and the observations of such processes requires a level of model complexity that is not supported by the available data.

### Lessons learned and future research

Overall, results on combined effects were difficult to interpret conclusively and depended on the way the model was formulated. Given the complementary log–log function used to link health and survival probability (Pirotta et al., 2023), this may be due in part to the additive effect of multiple stressors on the link scale (i.e., health) being non‐additive on the response scale (i.e., survival) (Pirotta et al., 2022). However, it is also likely that the ambiguity of some of the results reflects the complexity of the question under analysis and the lack of sufficient data granularity to answer it. Our state‐space model, while mechanistically informed, remains an empirical model where relationships are estimated from data (Auger‐Méthé et al., 2021). Therefore, its development hinged on long‐term longitudinal measurements of individual health and life history outcomes. Our health metric effectively captures variations in survival (Pirotta et al., 2023). However, additional markers that can be collected remotely are needed to monitor the more subtle changes in individual health that are likely to drive whether an animal reproduces successfully or is able to grow (National Academies, 2017; Tyack et al., 2022), and to reflect the different mechanisms through which stressors operate and combine. For example, we investigated the use of (i) the concentration of thyroid hormone metabolites in fecal samples and (ii) relative body width derived from drone imagery as additional metrics of nutritional state, but the comparatively small sample size of these novel datasets meant that they did not contribute to the estimation of individual health. The inclusion of new data streams will thus have to be validated and balanced against the strength of the existing, long time series of visual health data.

Modeling individual exposure in space and time required extensive spatiotemporal stressor data, which were challenging to collate for a species that ranges over thousands of kilometers and lives for several decades. An extensive collaboration among many researchers from diverse disciplines enabled the compilation of the best information available on these stressors, that is, range‐wide levels and distribution of vessel traffic (Redfern et al., 2024; Spadon et al., 2024), an integrative tool for estimating entanglement risk (Miller et al., 2024, 2025), and a transboundary model to predict the availability and abundance of NARW main prey species (Plourde et al., 2024). However, this exercise has also imposed some strong assumptions on how stressors have changed over time (e.g., using fisheries landings data to inform the trend in entanglement risk, or extrapolating the time series of prey availability prior to 1999) or outside covered areas (e.g., extending the entanglement risk surface into Canadian waters). Moreover, it forced the coarsening of spatial and temporal resolutions to scales where important heterogeneity in stressor exposure may be missed (e.g., the mismatch between NARW fine‐scale foraging decisions and our index of prey abundance (Hudak et al., 2023), the shifting spatial distributions of fisheries and whale habitat within regions, or the inability to capture heterogeneous or localized entanglement risk, vessel strike risk, and prey availability in the Gulf of St Lawrence and Northeastern US regions). There is also a mismatch between the large polygons we used and the spatial scale at which management decisions are normally made. A separate simulation study would be needed to optimize the trade‐off between extending the spatiotemporal scope of the model with increasing uncertainty against the accurate estimation of combined stressor effects, since we have shown that the influence of these assumptions can be large. Similarly, additional mining of existing datasets could improve these inputs, representing a priority for future research. Moreover, we had to completely exclude other, potentially important stressors (e.g., anthropogenic noise and chemical contaminants; Moore et al., 2021), for which sufficient data were not available or not at the correct extent and resolution. These will likely be common obstacles to modeling the effects of multiple stressors for many species, and we encourage investment toward a coordinated compilation of stressor maps at a regional or ecosystem level (e.g., led by regulatory or advisory agencies), which could then be made available for specific assessments. In turn, monitoring how stressor levels change as management measures are implemented will also be important.

The way in which individual whale distribution was modeled, and the data used for the modeling, also had a strong influence on the results. Spatial distribution can be informed using individual sightings, but more data on effort levels spanning multiple national jurisdictions are needed to appropriately capture occurrence and density patterns. For NARW, the fact that sightings contributed to the Consortium database did not always include effort data was limiting, and we have shown the different spatial distribution patterns emerging from the alternative use of an existing density surface model (Appendix S1: Figure S1). In turn, this version required strong assumptions to extend the model to Canadian regions and underestimated NARW occurrence in some important periods and areas (Appendix S1: Section S2.2). Because ecological systems are dynamic, and the environment that populations of concern inhabit can change rapidly (Parmesan & Yohe, 2003), being able to track geographic shifts and their consequences for spatiotemporal exposure rates to stressors is critical (Bontrager et al., 2024; Wickwire et al., 2011). In general, environmental variation is an important driver of the dynamics of wildlife populations and will represent a common backdrop to other anthropogenic stressors requiring management (Kefford et al., 2023; National Academies, 2017), but our understanding of ecosystem‐level processes and the ecological interactions therein is often incomplete (Tallis et al., 2010). Systematic data on NARW dynamic distribution, integrating information on variable detectability in different regions, and a continued exploration of the underpinning environmental drivers will be critical to inform conservation efforts (Roberts et al., 2024).

Here, we have explicitly explored different model formulations to highlight that several mechanistic assumptions are required to complement data limitations, and these can alter the results. Even in this data‐rich species, major assumptions were necessary to fill gaps in the data. Investigating the influence of model structure and assumptions in such a case study is particularly relevant before applying similar approaches to other, less studied species. Comparable limitations will emerge across contexts, but the development and evaluation of models based on available information can help prioritize future research directions and guide adaptive management interventions. Further complexity could be built into our model; for example, including an explicit observation model to account for potential differences in carcass recovery by cause (Linden et al., 2024) or additional pathways through which stressors may affect vital rates (e.g., female recruitment; Reed et al., 2024). However, we argue that the individual‐level focus of our model should be retained, to appropriately capture individual heterogeneity in exposure, health, and life history performance (Moore et al., 2021).

### Management applications and conclusions

Ultimately, these modeling efforts can support the design and evaluation of management scenarios that ensure the viability of a species (Tyack et al., 2022). As an example, for NARW, this would involve the integration of our results within ongoing conservation efforts. We have shown how the outputs of our model could be incorporated into a predictive framework, like an existing NARW PVA tool (Runge et al., 2023). In our example, simulated population trajectories evaluating the impacts of entanglement and prey suggested that, while reducing entanglement risk is critical to ensuring the population's viability (Runge et al., 2023), the current trend in asymptotic body length and its effect on calving probability (Pirotta et al., 2024) may limit NARW's ability to recover successfully in the long term (Figure 5). Therefore, it will be useful to explicitly monitor changes in body length as part of future management plans. Though we did not identify an effect of prey in the PVA, we compared recent, low prey conditions with historical variation including periods of both low and high prey. Moreover, prey is probably involved in the process driving the decline in asymptotic length, although we did not identify this relationship in our analyses. These results also suggest that, given the difficulties in compiling robust stressor data, body length could be a useful trait to measure as an integrator of the long‐term prey conditions and stressors experienced by an individual (Clements & Ozgul, 2018).

This simulation exercise did not consider scenarios of reduced vessel strikes, which constitute an important threat to NARW (Sharp et al., 2019). Moreover, several analytical inconsistencies between our model and the PVA tool mean that the absolute population numbers simulated from this integration may currently be inappropriate for use in management. In particular, the random walk nature of the health process is not suitable for forward prediction, as there is no mechanism to constrain the temporal trend in health within reasonable boundaries; nonetheless, accounting for prolonged temporal autocorrelation in health and vital rates is warranted in such a long‐lived species (Appendix S1: Section S14). Therefore, going forward, a concerted effort to merge our individual‐based perspective with other population‐level analyses (e.g., Reed et al., 2024; Runge et al., 2023) is advised, as this will benefit from the combined strengths of different approaches and inform management amidst several available methods.

In conclusion, in this study, we have moved away from the question of whether stressor interactions are additive, synergistic, or antagonistic (Pirotta et al., 2022). Instead, we have focused on building a spatially explicit model that can be used to examine the combined effects of stressors on individual vital rates, which will ultimately support the quantification of the cumulative risk for this population of high conservation interest (Tyack et al., 2022). In this process, we have learned lessons about the data and assumptions required to assess combined effects in populations of long‐lived species, where measuring population‐level consequences directly is too imprecise or takes too long to meet pressing management and conservation agendas (Taylor et al., 2007; White, 2019). These lessons should be valuable across a range of terrestrial and aquatic systems with similar characteristics, where effective conservation efforts necessitate efficiency and caution in selecting combinations of stressors to address. Further data and methodological development are needed to bring the science of multiple stressors on par with the scale of the challenges imposed by the current climate and biodiversity crises, requiring extensive investment and large, interdisciplinary collaborations.

## AUTHOR CONTRIBUTIONS

*Conceptualization*: Enrico Pirotta, Peter L. Tyack, Catriona M. Harris, Robert S. Schick, and Len Thomas. *Data curation*: Enrico Pirotta, Jeffrey Adams, Michael J. Asaro, Phil J. Bouchet, John W. Durban, Amy R. Knowlton, Scott D. Kraus, Caroline Lehoux, Erin Meyer‐Gutbrod, Alicia S. Miller, Carolyn A. Miller, Michael J. Moore, Heather M. Pettis, Stéphane Plourde, Nicholas R. Record, Jason J. Roberts, Robert S. Schick, Burton Shank, Laura Solinger, and Marisa L. Trego. *Formal analysis*: Enrico Pirotta and Len Thomas. *Funding acquisition*: Enrico Pirotta, Peter L. Tyack, Catriona M. Harris, Amy R. Knowlton, Scott D. Kraus, Michael J. Moore, Heather M. Pettis, Robert S. Schick, and Len Thomas. *Investigation*: John W. Durban, Amy R. Knowlton, Scott D. Kraus, Carolyn A. Miller, Michael J. Moore, and Heather M. Pettis. *Methodology*: Enrico Pirotta, Peter L. Tyack, Jeffrey Adams, Phil J. Bouchet, Daniel E. Crocker, Ailsa J. Hall, Catriona M. Harris, Amy R. Knowlton, Caroline Lehoux, Daniel W. Linden, Erin Meyer‐Gutbrod, Carolyn A. Miller, Patrick J. O. Miller, Michael J. Moore, Theoni Photopoulou, Stéphane Plourde, Nicholas R. Record, Jessica V. Redfern, Jason J. Roberts, Robert S. Schick, Burton Shank, Laura Solinger, Marisa L. Trego, and Len Thomas. *Project administration*: Peter L. Tyack, Catriona M. Harris, and Len Thomas. *Software*: Enrico Pirotta and Daniel W. Linden. *Supervision*: Peter L. Tyack, Catriona M. Harris, and Len Thomas. *Validation*: Enrico Pirotta. *Visualization*: Enrico Pirotta and Phil J. Bouchet. *Writing—original draft*: Enrico Pirotta. *Writing—review and editing*: Enrico Pirotta, Peter L. Tyack, Jeffrey Adams, Michael J. Asaro, Phil J. Bouchet, Daniel E. Crocker, John W. Durban, Ailsa J. Hall, Catriona M. Harris, Amy R. Knowlton, Scott D. Kraus, Caroline Lehoux, Daniel W. Linden, Caroline P. Good, Erin Meyer‐Gutbrod, Alicia S. Miller, Carolyn A. Miller, Patrick J. O. Miller, Michael J. Moore, Christopher D. Orphanides, Eric M. Patterson, Heather M. Pettis, Theoni Photopoulou, Stéphane Plourde, Nicholas R. Record, Jessica V. Redfern, Jason J. Roberts, Robert S. Schick, Burton Shank, Laura Solinger, Marisa L. Trego, and Len Thomas.

## FUNDING INFORMATION

This work was supported by the Office of Naval Research (N000142012697, N000142112096) and the Strategic Environmental Research and Development Program (RC20‐1097, RC20‐7188, RC21‐3091).

## CONFLICT OF INTEREST STATEMENT

The authors declare no conflicts of interest.

## ETHICS STATEMENT

This work was approved by the Ethics Committee of the School of Biology at the University of St Andrews (SEC20015). The study uses historical and published data that were collected under a series of US National Marine Fisheries Service, and Canadian Department of Fisheries and Oceans, Scientific Research Permits.

## Supporting information


Appendix S1.

## Data Availability

Data and code (Pirotta et al., 2025) are available in the Open Science Framework at https://osf.io/9tzvf.
