# Supplementary material for: The combined effects of multiple stressors in an endangered, long‐lived species: Lessons learned and ways forward
Source: Ecol Appl. 2025 Dec 8;35(8):e70144. doi: 10.1002/eap.70144 (PMC12683700; doi:10.1002/eap.70144)
Supplement: Supplementary file 1 — Appendix S1. [file EAP-35-e70144-s001.pdf]

Appendix S1

The combined effects of multiple stressors in an endangered, long-lived species: Lessons learned and ways forward

Enrico Pirotta, Peter L. Tyack, Jeffrey Adams, Michael J. Asaro, Phil J. Bouchet, Daniel Crocker, John W. Durban, Ailsa J. Hall, Catriona M. Harris, Amy R. Knowlton, Scott D. Kraus, Caroline Lehoux, Daniel W. Linden, Caroline P. Good, Erin Meyer-Gutbrod, Alicia S. Miller, Carolyn A. Miller, Patrick J. O. Miller, Michael J. Moore, Christopher D. Orphanides, Eric M. Patterson, Heather M. Pettis, Theoni Photopoulou, Stéphane Plourde, Nicholas R. Record, Jessica V. Redfern, Jason J. Roberts, Robert S. Schick, Burton Shank, Laura Solinger, Brandon L. Southall, Marisa L. Trego, Len Thomas

Ecological Applications

Table of Contents

Section S1: Summary of modelling approach developed in Pirotta et al. (2023, 2024) 4

Section S1.1: Process model 4

Section S1.2: Observation model 6

Section S2: Spatial distribution models 8

Section S2.1: Data-driven formulation (v1, North Atlantic Right Whale Consortium sightings) 8

Section S2.2: Model-based formulation (v2, Roberts et al. (2024) density surface model) .. 8

Section S3: Generating the surfaces for entanglement risk 12

Section S4: Processing of AIS data to infer vessel strike risk 15

Section S5: Integration of the predictions of *Calanus spp.* concentration in the right whale model 18

Section S6: Model for entanglement events 22

Section S7: Model for vessel strike events 25

Section S8: Modelling the combined effects of multiple stressors 26

Section S8.1: Combined effects on health 26

Section S8.2: Combined effects on length 26

Section S8.3: Combined effects on exposure 27

Section S9: Model notation 28

Section S10: Prior and posterior distributions of model parameters across the three model formulations 32

Section S10.1: Spatial model (data-driven formulation, v1) 32

Section S10.2: Spatial model (model-based formulation, v2) 35

Section S10.3: Non-spatial model (Pirotta et al. 2024, v3) 38

Section S10.4: Combined effects (across model formulations) as defined in section S8 40

Section S11: Predicted exposure to stressors 47

Section S11.1: Data-driven spatial distribution model (v1, from NARW Consortium sightings) 47

Section S11.1.1: Entanglement probability 47

Section S11.1.2: Vessel strike probability 49

Section S11.1.3: Mean annual prey conditions 51

Section S11.2: Model-based spatial distribution model (v2, from Roberts et al. 2024) 52

Section S11.2.1: Entanglement probability 52

Section S11.2.2: Vessel strike probability 54

Section S11.2.3: Mean annual prey conditions 56

Section S12: Estimating the trend in entanglement risk prior to 2015 57
Section S13: Example of a combined effect of two stressors 59
Section S14: Assessing future stressor scenarios using model results in a population viability analysis 60
Section S14.1: Change to survival-health link function 60
Section S14.2: Calculating annual survival 60
Section S14.3: Accounting for temporal autocorrelation in survival probability 61
Section S14.4: Exposure to stressors 63
Section S14.5: Model for fecundity 64
Section S14.6: PVA scenarios 64
Section S14.7: Results and discussion 65
Section S15: References 67

Section S1: Summary of modelling approach developed in Pirotta et al. (2023, 2024)

The model presented in this study builds on the Bayesian state-space model developed in Pirotta et al. (2023, 2024). The process model includes the effects of covariates on individual health (juvenile status, lactation status, and the stressors under analysis), a model for individual length, and the relationships between health, length and the two vital rates (survival and calving), while the observation model links underlying health to each of the four variables of the visual health assessment (VHA), length to photogrammetric measurements, and survival to individual sightings.

Section S1.1: Process model

The latent health of an individual (i = 1, 2,..., N) in a three-month time step (t = 2, 3,..., T_i), $h_{i,t}$, is modelled as a function of health in the previous time step and 2 time-varying intrinsic ($Z_{j,i,t}$, with j = 1, 2) and 9 time-varying extrinsic ($W_{k,i,t}$, with k = 1, 2,..., 9) covariates:

$$h_{i,t} \sim Normal\left(h_{i,t-1} + \sum_{j=1}^{2} \alpha_j \, Z_{j,i,t} + \sum_{k=1}^{9} \beta_k \, W_{k,i,t}, \sigma\right) \qquad S1$$

where α_j and β_k indicate the effects of each intrinsic and extrinsic covariate, respectively, and σ is the process standard deviation (fixed at σ = 0.1; Pirotta et al. 2023). Three-month time steps correspond to the intervals Dec-Feb, Mar-May, Jun-Aug, and Sep-Nov.

Intrinsic covariates include the effect of the transition from calf to juvenile status (i.e., $Z_{1,i,t} = 1$ in the Dec-Feb interval at the end of the birth year, and 0 otherwise) and the proportion of a three-month interval spent in lactation status (i.e., $Z_{2,i,t}$ varies between 0 and 1 depending on how many months in an interval a female was nursing a dependent calf). Extrinsic covariates include the effect of mean annual prey conditions, which applies in the Jun-Aug interval ($W_{1,i,t}$; see section S5 for details). Entanglements can cause immediate effects on health that differ with the severity of the event (a factor with three levels, indicated by dummy variables $W_{2,i,t}$, $W_{3,i,t}$ or $W_{4,i,t}$, which could take value 1 in the interval when an event occurred depending on whether it was scored as minor, moderate or severe, respectively, and were 0 otherwise). An entanglement can also result in an individual carrying gear for prolonged periods ($W_{5,i,t}$). As discussed in section S6 below, we modified the published version of the model to completely separate the immediate and prolonged effects (i.e., $W_{5,i,t} = 0$ in the first time step of an event). Vessel strikes can cause an immediate effect on health that differs depending on the injury type (a factor with four levels, indicated by $W_{6,i,t}$, $W_{7,i,t}$, $W_{8,i,t}$, $W_{9,i,t}$, which could take value 1 or 0 in the interval when a strike occurred, depending on whether the injury was scored as superficial, shallow, deep, or blunt, respectively). It should be noted that the order of the covariates in the process model for health was modified from Pirotta et al. (2023) for ease of implementation.

Latent health corresponds to the probability of surviving to the end of the current time step, $\vartheta_{i,t}$, on the complementary log-log (cloglog) link scale:

$$cloglog(\vartheta_{i,t}) = h_{i,t} \qquad S2$$

$$s_{i,t} \sim Bernoulli(\vartheta_{i,t}\, s_{i,t-1}) \qquad S3$$

where survival $s_{i,t} = 1$ when the individual is alive, and 0 when it is dead. Survival was set to 1 for all time steps between the first and last sighting of an individual and to 0 when an individual was found dead. Survival status is unknown and estimated for intervals between the last sighting and an observed death, or for two years after the last sighting (note that here we extend the time after the last sighting by one additional year, compared to Pirotta et al. 2023).

Calving is considered to occur in the Dec-Jan interval of years when females are available to calve (i.e., alive, sexually mature, not in a pregnancy year, and not resting in a year after calving; Rolland et al. 2016). If individual i is female, the probability of giving birth to a calf in available year y, $\varphi_{i,y}$, is related to health at some 3-month time step $t(y)$ prior to y ($h_{i,t(y)}$). Different lags between health and calving probability were tested in Pirotta et al. (2023), but they did not affect the estimated relationship. Therefore, health in the Sep-Nov interval just prior to the breeding season was used. The relationship between health and calving was modelled using a sigmoid function:

$$\varphi_{i,y} = g_i \left(\frac{m_{i,y}}{1 + e^{-\delta\,(h_{i,t(y)} - \mu)}} \right) \qquad S4$$

where $m_{i,y}$ is the asymptote of the sigmoid relationship representing the maximum calving probability for individual i in year y (see below), δ is the steepness of the sigmoid relationship, and μ is the value of health at which calving probability is 50% of the maximum. The binary variable g_i indicates whether a female is reproductive (i.e., has already calved or may do so in the future), with mean probability v. Calving probability is related to observations of calving events:

$$r_{i,y} \sim Bernoulli(\varphi_{i,y}\, s_{i,y}) \qquad S5$$

where $r_{i,y} = 1$ when the individual was seen with a calf on year y, 0 when it was sighted on that year but never with a calf, and is unknown otherwise (and imputed in the Bayesian model).

The length of individual i at each 3-month time step t ($L_{i,t}$) is modelled following Stewart et al. (2021) using a Gompertz growth function:

$$L_{i,t} = A_i\, e^{-C\, e^{-K\, a_{i,t}}} \qquad S6$$

where C regulates the position of the curve along the x-axis, K is the growth rate, and $a_{i,t}$ is individual age. As in Stewart et al. (2021), the individual-specific length asymptote A_i is modelled as a function of an individual's birth year (B_i):

$$A_i = A + \upsilon_1\, B_i + \varepsilon_i \qquad S7$$

where A is the intercept, υ_1 is the effect of birth year and ε_i is a normally distributed random effect with mean 0 and standard deviation σ_A. B_i was rescaled as $max(B_i - 1977, 0)$, so $B_i = 0$ when an individual was born prior to or in 1977 (the first year where a birth event was recorded). Therefore, A represents mean asymptotic length for animals born until 1977.

The effect of current length on the asymptotic calving probability in available years is modelled as

$$logit(m_{i,y}) = M_i + \zeta\, L^3_{i,t(y)} \qquad S8$$

where $M_i \sim Normal(\lambda, \chi)$ is a normally distributed, individual-specific intercept, and ζ is the effect of length cubed (using the same time step $t(y)$ prior to y as for the relationship with health).

Section S1.2: Observation model

The probability of sighting an individual alive in a time step is equal to sighting probability ρ multiplied by its survival state (i.e., whether the individual is currently alive):

$$p_{i,t} = \rho\, s_{i,t}. \qquad S9$$

A sighting then emerges from a random Bernoulli draw:

$$e_{i,t} \sim Bernoulli(p_{i,t}). \qquad S10$$

Note that, under this formulation, $e_{i,t} = 0$ when an individual was sighted as dead.

The four ordinal variables comprising the VHA (indicated below by subscript x) provided irregular observations of an individual's health. For each variable, a lower category was assumed to correspond to poorer health. Skin condition and cyamid presence have two categories, while body condition and rake marks have three categories (Pettis et al. 2004). Cyamid presence was only assessed for non-calf individuals, i.e., individuals older than 1 year (Pirotta et al. 2023). The probability of observing the first category of each variable ($\eta_{i,t,x,1}$), corresponding to the poorest health score, is expressed as a function of health using a logistic formulation, i.e.:

$$logit(\eta_{i,t,x,1}) = \gamma_{x,1} + \omega\, h_{i,t} \qquad S11$$

where $\gamma_{x,1}$ is an intercept parameter and ω controls the effect of health on the probability of observing this category. For variables with two categories, the probability of observing the second category is simply:

$$\eta_{i,t,x,2} = 1 - \eta_{i,t,x,1}. \qquad S12$$

For variables with three categories, the probabilities of observing the second and third category are expressed using an ordinal logit formulation, i.e.:

$$logit(Q_{i,t,x}) = \gamma_{x,2} + \omega\, h_{i,t} \qquad S13$$

$$\eta_{i,t,x,2} = Q_{i,t,x} - \eta_{i,t,x,1} \qquad S14$$

$$\eta_{i,t,x,3} = 1 - Q_{i,t,x} \qquad S15$$

where $\gamma_{x,2}$ is constrained to be greater than $\gamma_{x,1}$. For increasing health, the probability of observing higher categories is expected to increase, i.e., ω is expected to be negative (also note that ω was the same across health variables). Given the number of categories for a variable (c),

up to 3 ordinal observations (one per month) $o_{i,t,x,1:O_{i,t,x}}$ result from a multinomial draw with probabilities $\eta_{i,t,x,1:c}$, where $O_{i,t,x}$ is the number of observations for that variable in that time step:

$$o_{i,t,x,1:O_{i,t,x}} \sim Multinomial(\eta_{i,t,x,1:c}, O_{i,t,x}). \qquad S16$$

Note that the "$x{:}y$" notation is used to signify that the associated quantity is a vector, with the subscript running from x to y, e.g., $\eta_{i,t,x,1:c} = (\eta_{i,t,x,1}, \dots, \eta_{i,t,x,c})$.

Individual length and growth were informed using photogrammetric measurements derived from aerial images (Stewart et al. 2021, Pirotta et al. 2024). An observation model is included to describe measurement uncertainty, of the form:

$$P_{i,t} \sim Normal(L_{i,t}, \xi_d) \qquad S17$$

where $P_{i,t}$ is measured length for individual i in time step t, and ξ_d is the observation error, specific to each methodology d used for measuring camera altitude.

The model was extended here to include a spatial structure, spatially explicit stressor surfaces, and the combined effects of stressors as described in the main text and in sections S2-S8 below.

Section S2: Spatial distribution models

Section S2.1: Data-driven formulation (v1, North Atlantic Right Whale Consortium sightings)

In this formulation of the model, the proportion of time an individual North Atlantic right whale (NARW) i spent in each of the R = 7 regional polygons in each three-month interval t ($z_{t,i,1:R}$) was derived from the distribution of the sightings of that individual in that period (not corrected for effort), when the individual was observed at least once, with $\sum_{l=1}^{R} z_{t,i,l} = 1$. When the individual was not observed in a time step t, the proportion of time spent across regions was imputed in the model. This proportion was modelled to emerge from a Dirichlet distribution with a set of period- ($d(t)$; prior to 2010 and from 2010 onwards), age class- ($c'(i,t)$; adult, juvenile, calf), sex- ($f(i)$; male or female) and season-specific ($q(t)$; Dec-Feb, Mar-May, Jun-Aug, Sep-Nov) parameters, $\pi_{d(t),c'(i,t),f(i),q(t),1:R}$, i.e.:

$$z_{t,i,1:R} \sim Dirichlet\left(\pi_{d(t),c'(i,t),f(i),q(t),1:R}\right). \qquad S18$$

After preliminary exploration, the π parameters were set to have the same values for male and female juveniles and male and female calves. To support estimation for infrequently observed combinations of covariates, the parameters of the Dirichlet model for each period d, class c', sex f and season q were themselves assumed to emerge from a Dirichlet distribution representing the average distribution of the population in each three-month interval, i.e.:

$$\pi_{d,c',f,q,1:R} \sim Dirichlet\left(a'_{q,1:R}\right). \qquad S19$$

The posterior estimates of the π parameters are plotted in Fig. S1.

Section S2.2: Model-based formulation (v2, Roberts et al. (2024) density surface model)

In this version of the model, an individual i's proportion of time spent in each of the R = 7 regional polygons in each three-month interval t ($z_{t,i,1:R}$) was modelled as the product between the expected distribution of the average individual, $\tilde{l}_{t,i,1:R}$ [emerging from the relative abundance of NARW across regions in that season ($q(t)$; Dec-Feb, Mar-May, Jun-Aug, Sep-Nov) and period ($d(t)$; prior to 2010 and from 2010 onwards); see below], and the expected proportion of animals of the individual's age and sex class ($c''(i,t)$; calves, juveniles, adult females and adult males) in each region, given the period and the season, $\tilde{p}_{d(t),q(t),c''(i,t),1:R}$. The results were rescaled so that proportions across regions summed to 1, i.e.:

$$z_{t,i,1:R} = \frac{\tilde{l}_{t,i,1:R}\ \tilde{p}_{d(t),q(t),c''(i,t),1:R}}{\sum_{l=1}^{R} \tilde{l}_{t,i,l}\ \tilde{p}_{d(t),q(t),c''(i,t),l}}\ . \qquad S20$$

The expected distribution given the relative abundance of NARW across regions, $\tilde{l}_{t,i,1:R}$, was modelled to emerge from a Dirichlet distribution with season- and period-specific parameters, which were derived from the density surface model outputs in Roberts et al. (2024). Specifically, monthly density surfaces are available for each of the two periods 2003-2009 and

2010-2019, capturing the shift in distribution observed from 2010 onwards (Meyer-Gutbrod et al. 2023); we assumed that the distribution prior to 2003 was the same as in the first period. These monthly surfaces result from the outputs of a set of Generalised Additive Models (GAMs) for different portions of the NARW range that are then collated (Roberts et al. 2024), except for Cape Cod Bay (CCB), where monthly, design-based estimates of abundance and standard error derived from (Ganley et al. 2019) are distributed uniformly across the cells in the region. Two-hundred posterior simulations were generated from the GAMs using a Metropolis-Hastings sampler to propagate the uncertainty in model predictions; where a fitted GAM was not available (CCB), we drew from a lognormal distribution using the month's density and standard error. For each posterior simulation, we took the mean of the three monthly rasters in a three-month interval, given the period, and aggregated the densities into abundance of individuals in each of the five regional polygons covering U.S. waters (i.e., the extent of the model by Roberts et al. 2024).

As a temporary solution to the lack of comparable density information in Canadian waters, we assumed that the mean ratio of the number of sightings to abundance in the Northeastern U.S. region in each season and period was the same as the ratio of number of sightings to abundance in Canadian waters, and thus used the number of sightings and this scalar to infer the abundance in each of the two Canadian regions in each season and period. This critical knowledge gap should be targeted by future efforts to model whale distribution in these regions together with their distribution in U.S. waters. Moreover, future work should consider splitting the polygon for the Gulf of St Lawrence into two smaller polygons to reflect the profound differences in whale distribution and habitat use between the northern and southern parts of the Gulf (St-Pierre et al. 2024).

Finally, we calculated the proportional abundance in each regional polygon (given the season and period) and fitted a Dirichlet distribution to the 200 realisations. The parameters of these distributions ($a''_{d(t),q(t),1:R}$) were used in our model to determine the average individual's expected distribution across regions in each time step:

$$\tilde{l}_{t,i,1:R} \sim Dirichlet\left(a''_{d(t),q(t),1:R}\right). \qquad S21$$

The demographic class of individuals at each sighting e, $c''(i,e)$, emerged from the period- ($d(e)$; prior to 2010 and from 2010 onwards) and season-specific ($q(e)$; Dec-Feb, Mar-May, Jun-Aug, Sep-Nov) proportions of animals in each demographic class in the region where the sighting occurred, $l(e)$, via a categorical distribution:

$$c''^{(i,e)} \sim Categorical\left(\tilde{p}_{d(e),q(e),1:4,l(e)}\right). \qquad S22$$

To visualise the expected spatio-temporal distribution of different demographic classes, we plotted the a'' parameters of the Dirichlet distribution derived from the density surface model outputs, multiplied by the posterior estimates of the proportion of animals of a given demographic class in each region (i.e., the $\tilde{p}$ parameters), and rescaled the results so that they summed to 1 (Fig. S1). To capture the posterior uncertainty around the $\tilde{p}$ parameters for this visualization, we resampled 1,000 values using the posterior mean and standard deviation.

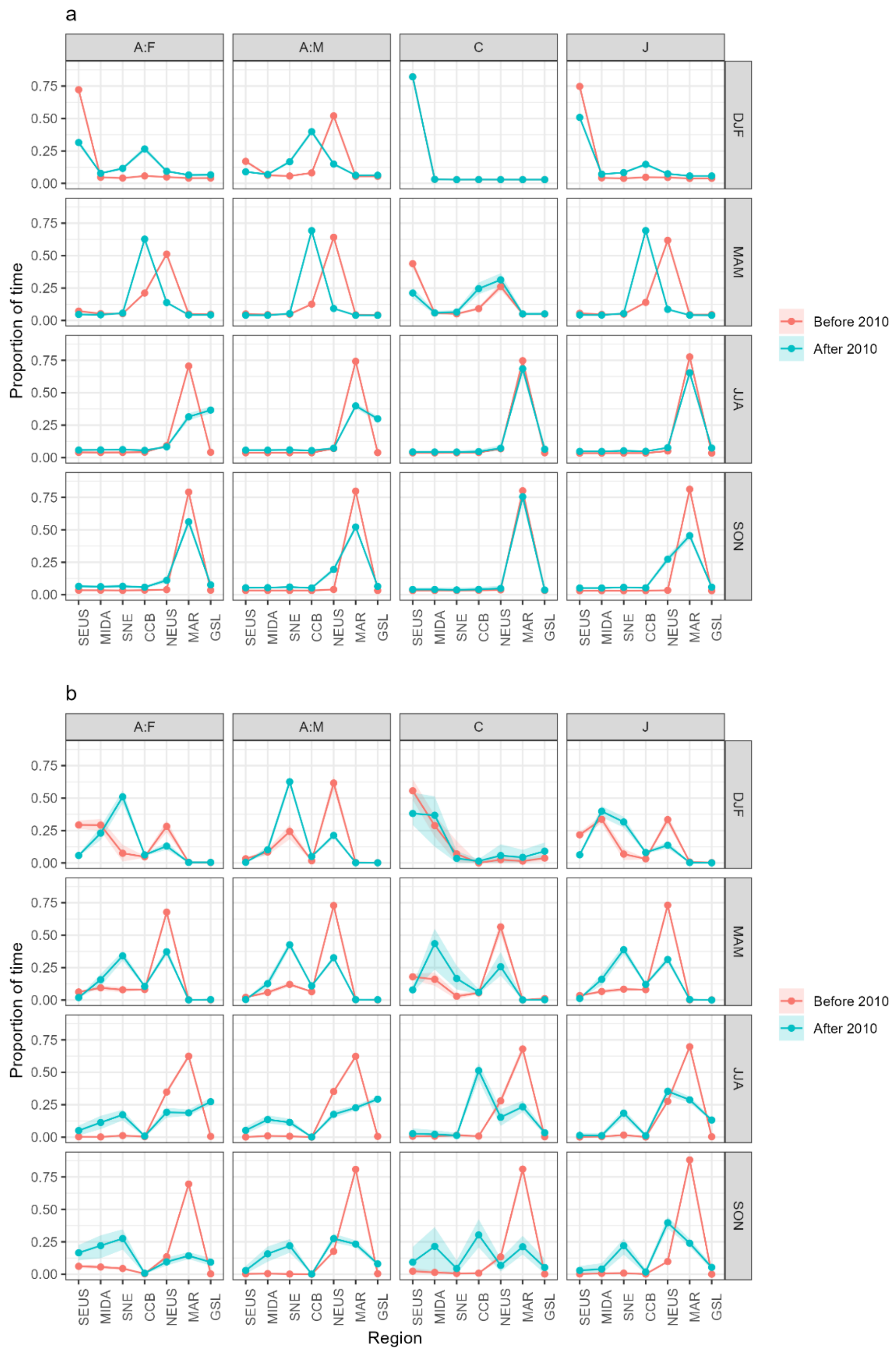

Figure S1. Expected spatial distribution of each demographic class (adult females, A:F; adult males, A:M; calves, C; juveniles, J) in each three-month interval (indicated by the initials of the corresponding months; DJF, MAM, JJA and SON) across the seven regional polygons (Southeastern U.S., SEUS; Mid-Atlantic, MIDA; Southern New England, SNE; Cape Cod Bay, CCB; Northeastern U.S., NEUS; Maritimes, MAR; Gulf of St Lawrence, GSL), before and after the shift in distribution around 2010. In a, this is represented by the posterior estimates of the

parameters π of the Dirichlet distribution for the proportion of time an individual spent in different regions in each time step (model v1). Dots and lines represent the posterior median, while the ribbon is the 95% credible interval. In b, the expected spatial distribution of individuals belonging to each demographic class is represented by the product between the α'' parameters of the Dirichlet distribution derived from the density surface model outputs, and the posterior estimates of the proportion of animals of that demographic class in each region ($\tilde{p}$ parameters), rescaled so that they summed to 1 (model v2). To capture the posterior uncertainty around the $\tilde{p}$ parameters, we resampled 1,000 values using the posterior mean and standard deviation.

Section S3: Generating the surfaces for entanglement risk

The baseline risk outputs of the Woods Hole Analysis of Line Entanglement Decision Support Tool (WHALE DST; Miller et al. 2024, 2025) were summed at the scale of our regional polygons for each month of the year. Monthly values were divided by the total whale densities in each region to remove the scaling of risk between regions (because individual spatial distribution across regions is estimated separately in our model), while retaining the relative scaling within a region (because aggregated values should capture NARW heterogenous use of space therein). Finally, resulting values were summed across the months in each three-month period. Hereafter, we use the term 'risk' to refer to this aggregated metric, but it should be noted that this differs from the meaning of the term in the WHALE DST.

As the WHALE DST only covers U.S. waters, and in the absence of comparable risk estimates for Canadian waters, we extrapolated entanglement risk into the two Canadian regional polygons by scaling the total risk in northern U.S. regions by the ratio between lobster and crab fisheries landings within each Canadian region and landings from those fisheries in northern U.S. regions. This ratio was estimated based on data sourced from the Northwest Atlantic Fisheries Organization (NAFO, https://www.nafo.int/) and was taken to be 1.11 for the Gulf of St Lawrence and 0.03 for the Maritimes region. We used landings data from 2015–2016, because exploration of the NAFO data suggested that landings were consistently reported across the U.S. and Canadian regions of interest in those two years. This extrapolation should be treated as a temporary solution while the relevant data are compiled for Canada; catch per unit effort in Canadian lobster and crab fisheries tend to be higher than in the U.S., for concurrently lower effort, so this extrapolation is likely overestimating fishing effort and gear density in Canadian regions. Moreover, it does not account for the heterogeneity in fishing effort (and resulting entanglement risk) within these large Canadian polygons (e.g., in the southern vs. northern Gulf of St Lawrence). The relative entanglement risk across regional polygons is reported in Fig. S2.

WHALE DST outputs were assumed to broadly reflect entanglement risk in the period 2015-2019 (Miller et al. 2024, 2025), although the range of years differed for each fishery group, as detailed in the WHALE DST report. For previous years (1970-2014), we scaled WHALE DST values using the trend in landings from fisheries that pose the greatest entanglement risk to NARW, i.e., lobster and crab fisheries in northern U.S. regions (Cape Cod Bay, Southern New England, and Northeastern U.S.) and gillnet fisheries in southern regions (mid-Atlantic and Southeastern U.S.). Landings data were obtained from the Atlantic Coastal Cooperative Statistics Program (ACCSP, https://www.accsp.org/; note that gillnet landings data only extend to 1992, prior to which we assumed that landings did not change; in section S12 we explore an alternative solution, estimating a regional trend in entanglement risk prior to 2015 from the data). The resulting trends in relative risk by region are reported in Fig. S3. The extrapolation of entanglement risk in time should be treated with extreme caution, because it ignores important shifts in the distribution of fisheries and whales within each region, which likely affected the spatial patterns of risk. Moreover, fisheries landings and effort have not necessarily tracked well over the long temporal window that was considered here, limiting the usefulness of landings data in capturing the density of fishing gear in the water.

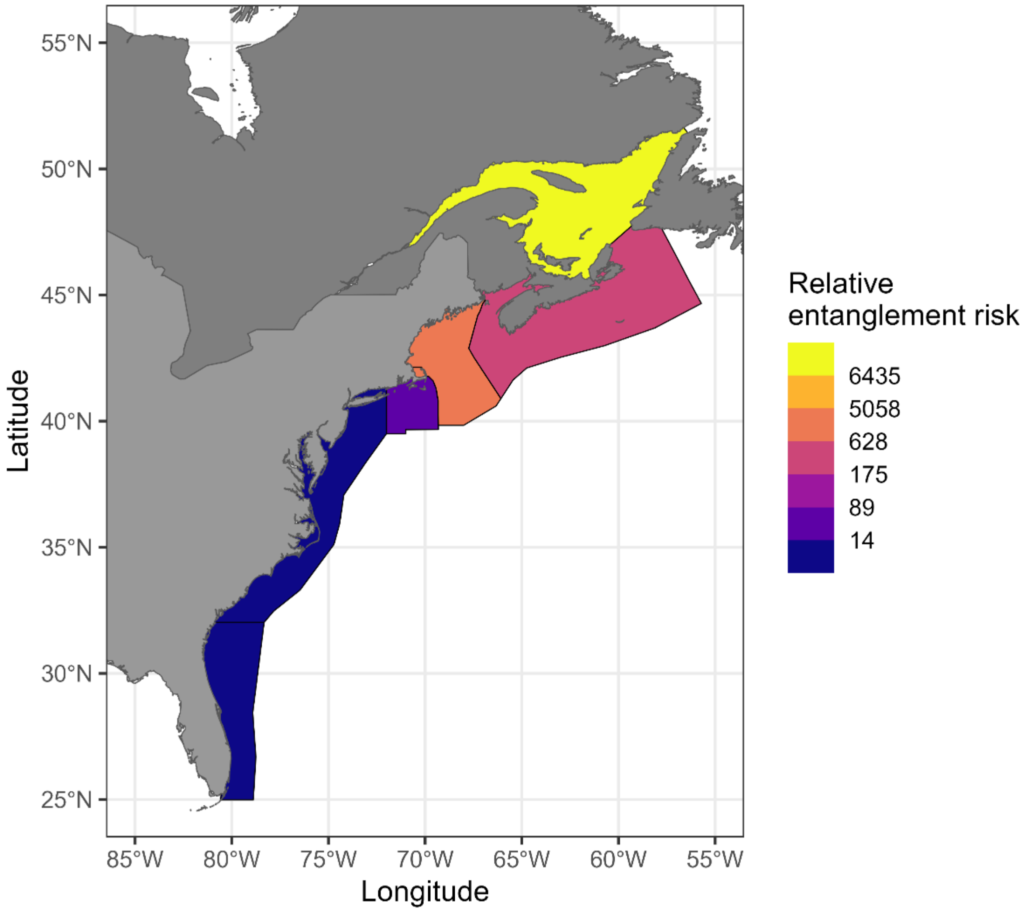

Figure S2. Example of relative entanglement risk across regional polygons derived from the WHALE DST (Miller et al. 2024, 2025) for the Jun-Aug interval (broadly reflecting entanglement risk in the period 2015-2019), in the five U.S. regions, and expanded into Canadian regional polygons. Entanglement risk in Canadian regional polygons was obtained from scaling the total risk in northern U.S. regions (i.e., Cape Cod Bay, Southern New England, and Northeastern U.S.) by the ratio between lobster and crab fisheries landings within each of the two Canadian regions and landings from those fisheries within northern U.S. regions.

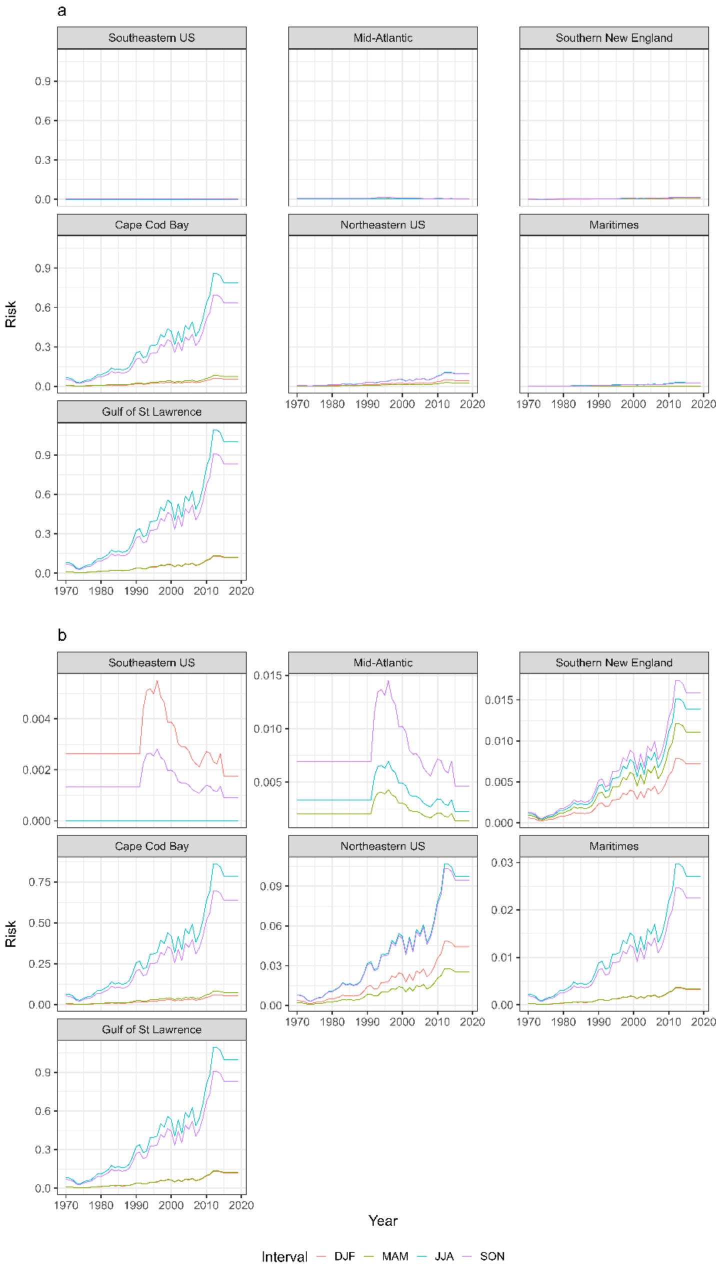

Figure S3. Temporal trend in relative entanglement risk per region per three-month interval (in a, using the same y-axis, and in b, allowing the y-axis to vary), as derived from the outputs of the WHALE DST scaled over time using the trends in landings data from the Atlantic Coastal Cooperative Statistics Program (lobster and crab fisheries in northern regions, and gillnet fisheries in southern regions). Risk in Canadian regions are scaled from the risk in northern U.S. regions using the landings of lobster and crab fisheries. Three-month intervals are indicated using the initial letters of the corresponding months. It should be noted that the estimated risk in Cape Cod Bay does not fully reflect the ban on fixed gear use in the Bay between February and April after 2015; this is likely due to (i) the three-month intervals not aligning with the closure period (i.e., the DJF interval includes Dec and Jan, and the MAM interval includes May); and (ii) the regional polygon for Cape Cod Bay not aligning perfectly with the Bay and including some spatial grid cells where the ban is not in place.

Section S4: Processing of AIS data to infer vessel strike risk

We used Automatic Identification System (AIS) data from 1 January 2019 through 31 December 2019 to characterise vessel traffic in NARW's range and derive a surface for vessel strike risk. Data were received from low-orbiting satellite constellations (ORBCOMM, https://www.orbcomm.com/eu) and terrestrial stations (USCG Nationwide Automatic Identification System) for U.S. waters, and obtained through the MERIDIAN initiative at Dalhousie University (https://meridian.cs.dal.ca/) for Canadian waters. Carrying an operational AIS transceiver is compulsory in the U.S. for: vessels that are 65 ft (~19.8 m) in length or longer; towing vessels of 26 ft (~7.9 m) in length or longer and more than 600 hp; vessels certified to carry more than 150 passengers; vessels that contain dangerous or flammable cargo; and vessels that can restrict or affect navigation of other vessels (https://www.navcen.uscg.gov/ais-requirements). Therefore, using AIS data could lead to underestimate vessel strike risk, because these data do not include smaller vessels.

AIS provides information on the position, type and speed of vessels; data points were processed as described in Redfern et al. (2024) to connect temporally consecutive locations and create transit segments for each vessel, while removing obvious location errors. Segments were then clipped to a 10 km x 10 km grid, and total vessel traffic in terms of distance travelled per cell per month was calculated by summing all clipped segments within a cell in each month of 2019 (Redfern et al. 2024). An example of the resulting surface is provided in Fig. S4. It should be noted that our risk estimates are based on co-occurrence of vessels and whales (Williams and O'Hara 2010, Redfern et al. 2013) and do not include vessel speed in the calculation of risk. This procedure was used because speed is generally included to account for lethality of a strike, rather than to influence the probability of its occurrence (Vanderlaan and Taggart 2007); in our model, we estimate the effect of a strike on health (and thus survival probability) separately.

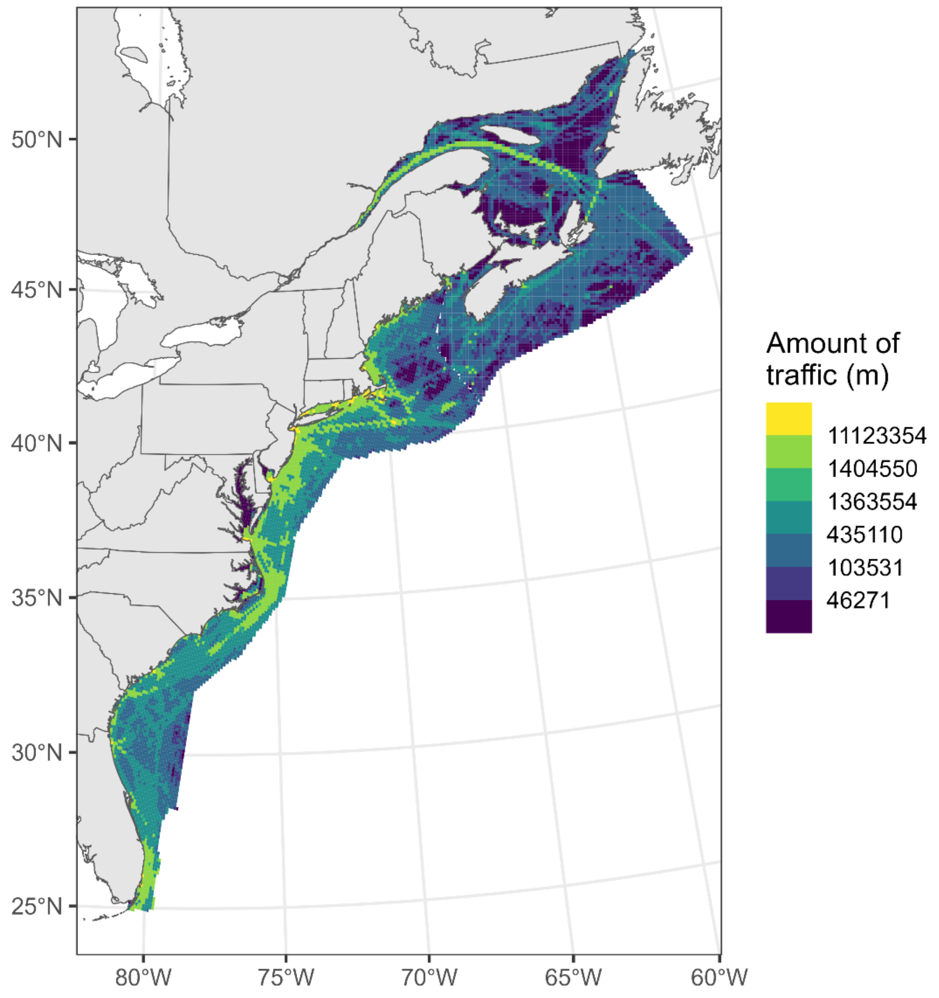

Figure S4. Total amount of vessel traffic in meters in June 2019, summarised over a 10 km x 10 km grid.

Monthly predicted NARW densities from Roberts et al. (2024) were also associated to each cell; however, because of the mismatch in spatial scale between the vessel and whale surfaces, we followed Redfern et al. (2024) and used the area-weighted average method of Woodman et al. (2019) to summarize the monthly predicted whale densities in the vessel traffic grid cells. We used the relative NARW densities across all cells falling in each regional polygon to calculate a weighted mean total distance travelled per region per month, which we assumed to reflect the risk of vessel strike for an individual entering that region in a given month; this ensured that the summarised risk per region accounted for the heterogeneous use of space of NARW (e.g., vessel traffic occurring in high whale density areas should be weighted more than the same levels in a portion of a regional polygon that whales use less regularly). Monthly mean risk values per region were aggregated at the three-month temporal scale of our model by taking the mean risk over the three months in each season (Dec-Feb, Mar-May, Jun-Aug, Sep-Nov).

Given the shift in distribution assumed to have occurred from 2010 onwards, and the resulting two sets of predicted monthly densities available from Roberts et al. (2024), these calculations were repeated to compute the weighted mean risk per region per three-month interval before 2010 and from 2010 onwards. Finally, we divided risk values by the maximum value observed across regions, seasons, and periods. The seasonal variation in relative risk per region is reported in Fig. S5; note that 1) relative risk values account for the heterogeneous distribution of NARW within a regional polygon, but not for the relative densities of NARW across polygons in a given three-month interval; 2) spatio-temporal patterns of risk in the first period (i.e., before 2010) do not account for the overall trend in vessel strike risk estimated in the model; 3) relative risk values are converted to actual vessel strike probabilities in the model.

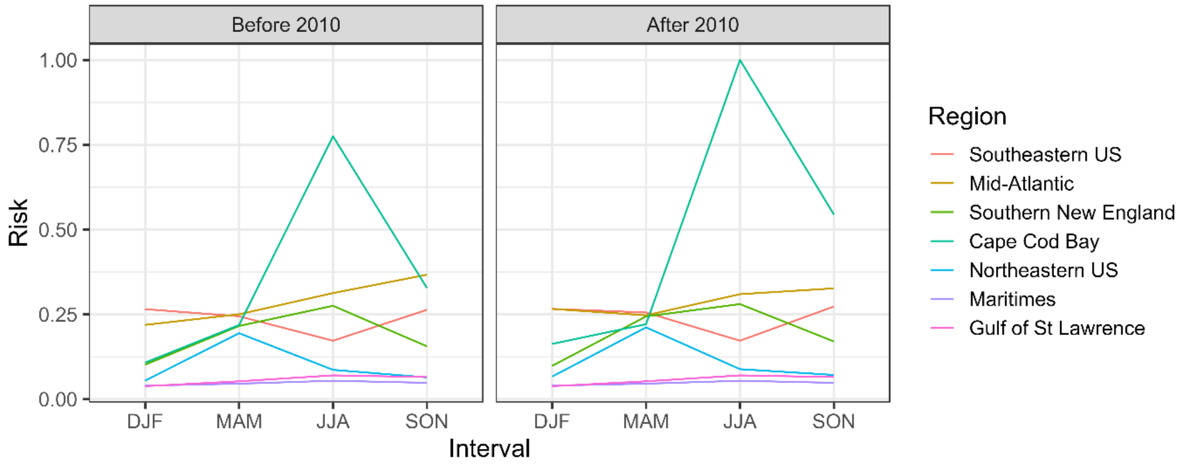

Figure S5. Variation in relative vessel strike risk per region per three-month interval, before 2010 and from 2010 onwards. Intervals are indicated using the initial letters of the corresponding months. Note that this represents the relative risk in each region over the year, and as such it does not account for NARW occurrence across regions over time or the temporal trend in traffic estimated in the model.

Section S5: Integration of the predictions of *Calanus spp.* concentration in the right whale model

Prey distribution and density were obtained from Plourde et al. (2024) model for three copepod species in the genus *Calanus* (*C. finmarchicus, C. hyperboreus*, and *C. glacialis*; late developmental stages). *C. finmarchicus* is a major component of the NARW diet, as indicated by the analyses of zooplankton communities near feeding NARW and the close correspondence between their vertical distribution and NARW diving behavior (e.g., Baumgartner et al. 2003). *C. hyperboreus* and *C. glacialis* may also be important prey, particularly given the larger body size of *C. hyperboreus* (Lehoux et al. 2020). *Calanus spp.* store lipids during their late stages and therefore provide an important energy source for NARW (Davies et al. 2012). NARW baleen plates efficiently capture *Calanus spp.* compared to smaller prey, such as early stage of *Calanus* and other small copepods, or more mobile prey, such as adult krill (Lehoux et al. 2020). Other copepod and zooplankton taxa were observed around feeding NARW, but their lower aggregation, energy content, or smaller size suggest that they are less important components of the NARW diet across the entire season and area (Mayo and Marx 1990, Pendleton et al. 2009, Sorochan et al. 2021).

The model by Plourde et al. (2024) provides monthly predictions of the concentration (g/m^3) of the three *Calanus* species in 10 m vertical layers, at a 0.083° spatial resolution, for the period 1999-2019. We summed the concentrations across the three species in each depth layer. Because we are adding concentrations in grams, the summation accounts for differences in size between species; temporal changes in biomass and stages composition are also considered in the model by Plourde et al. (2024), while the caloric content per unit of weight is thought to be similar (Davies et al. 2012). It should be noted that this model does not capture other prey species targeted by NARW in Cape Cod Bay (Hudak et al. 2023) and possibly elsewhere (Carlowicz Lee et al. 2024).

We assumed that a whale would target the layer with maximum predicted concentration within the species' diving range (0-306 m; Baumgartner and Mate 2003, 2005, Baumgartner et al. 2017) in each cell (e.g., Fig. S6). This assumption could be explored and revisited using a fine-scale, spatio-temporally explicit investigation of NARW foraging decisions, because depth may affect the availability of prey to individual whales and the energetic costs associated with diving to reach a foraging patch.

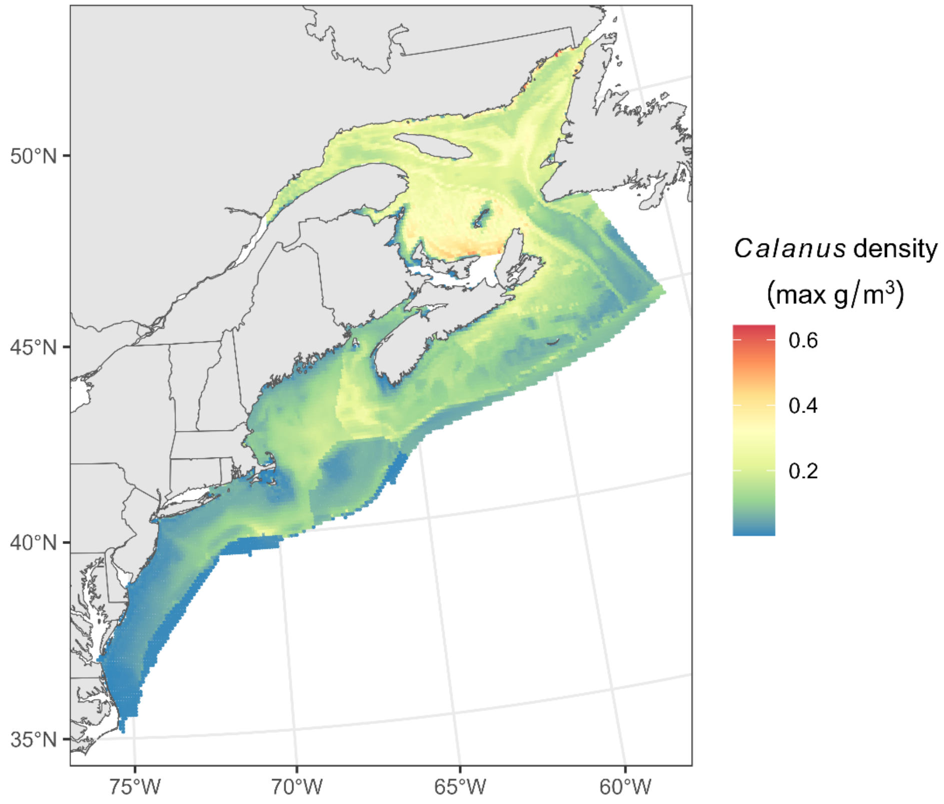

Figure S6. Maximum concentration (g/m³) of copepods in the genus Calanus (C. finmarchicus, C. hyperboreus and C. glacialis) in June 2019 in the depth range 0-306 m, at a 0.083° spatial resolution, as predicted by the model in Plourde et al. (2024).

Similarly to the entanglement and vessel strike risk surfaces, relative predicted NARW densities from Roberts et al. (2024) across each regional polygon were used to calculate a weighted mean of the maximum *Calanus* concentrations per cell, ensuring that the summarised prey conditions per region accounted for the heterogeneous use of space of NARW. Monthly mean values per region were then aggregated at the three-month temporal scale by taking the mean of the values of the three months in each season (Dec-Feb, Mar-May, Jun-Aug, Sep-Nov).

The resulting index of prey conditions was extrapolated for the period not covered by the *Calanus* model (1970-1998) by regressing the values of the index in 1999-2019 against the time series of annual anomalies of late-stage *Calanus finmarchicus* abundance from the Continuous Plankton Recorder (CPR) data in the Gulf of Maine (Pershing et al. 2005). Preliminary residual exploration suggested that the response variable should be log-transformed to meet the model's assumptions of normality and homoscedasticity. We also included an interaction of the relationship between the index derived from the *Calanus* model and the index derived from the CPR data with the three-month interval, as well as a season- and region-specific intercept (the most parsimonious subset of main effects and interactions was identified using Akaike Information Criterion). The resulting model had an adjusted R^2 of

0.94, suggesting a good fit to the data overall, but with variation among seasons and regions. The model was then used to predict the index of prey conditions in each region and season from the CPR data in the period 1970-1998; the complete time series is plotted in Fig. S7 (note that the index is not available for the southeastern U.S. region, because *Calanus* concentration is assumed to be 0 in this region). Because there are no CPR data from 1975 and 1976, the index was also missing for those two years and was imputed in the Bayesian model; specifically, we used a uniform prior distribution with lower and upper boundary corresponding to the minimum and maximum values observed in each region and three-month interval.

The extrapolation of prey conditions prior to 1999 using the CPR data presents several limitations. The *Calanus* data from Canadian waters informing the model by Plourde et al. (2024) include two species (*C. hyperboreus* and *C. glacialis*) that have different life history and phenology compared to *C. finmarchicus*, resulting in different seasonal patterns of occurrence in surface waters (Plourde et al. 2003, Blais et al. 2024). The CPR data are largely dominated by *C. finmarchicus* abundance and do not capture diapausing copepods. In contrast, *C. hyperboreus* is the main contributor to the total *Calanus* biomass in the Gulf of St Lawrence (Blais et al. 2024). *C. hyperboreus* and *C. glacialis* are also generally less available to CPR sampling during the year. These important limitations likely bias extrapolated biomass projections in the past (both in terms of overall abundance and seasonal dynamics). Therefore, future work should explore the consequences of these simplifying assumptions on the patterns of estimated *Calanus* availability in space and time and on the estimated combined stressor effects, with the goal of identifying an optimal trade-off between extending the spatio-temporal scope of the model against the increasing uncertainty in the data inputs.

Annual mean prey conditions, derived from combining the derived prey index with the spatial distribution of individual NARW across the four three-month intervals, were included as a covariate on individual health, but the effect was only estimated in the Jun-Aug interval. As discussed in Pirotta et al. (2023), the VHA data largely do not capture the seasonal variation in the health of an individual within each year; therefore, applying the effect of prey to multiple time steps of the year led to problems with its estimation (see Pirotta et al. 2023). We therefore only estimated the effect of mean annual prey conditions once per year and selected the Jun-Aug interval because it corresponds to the central part of NARW feeding season; however, using a different interval did not alter the results.

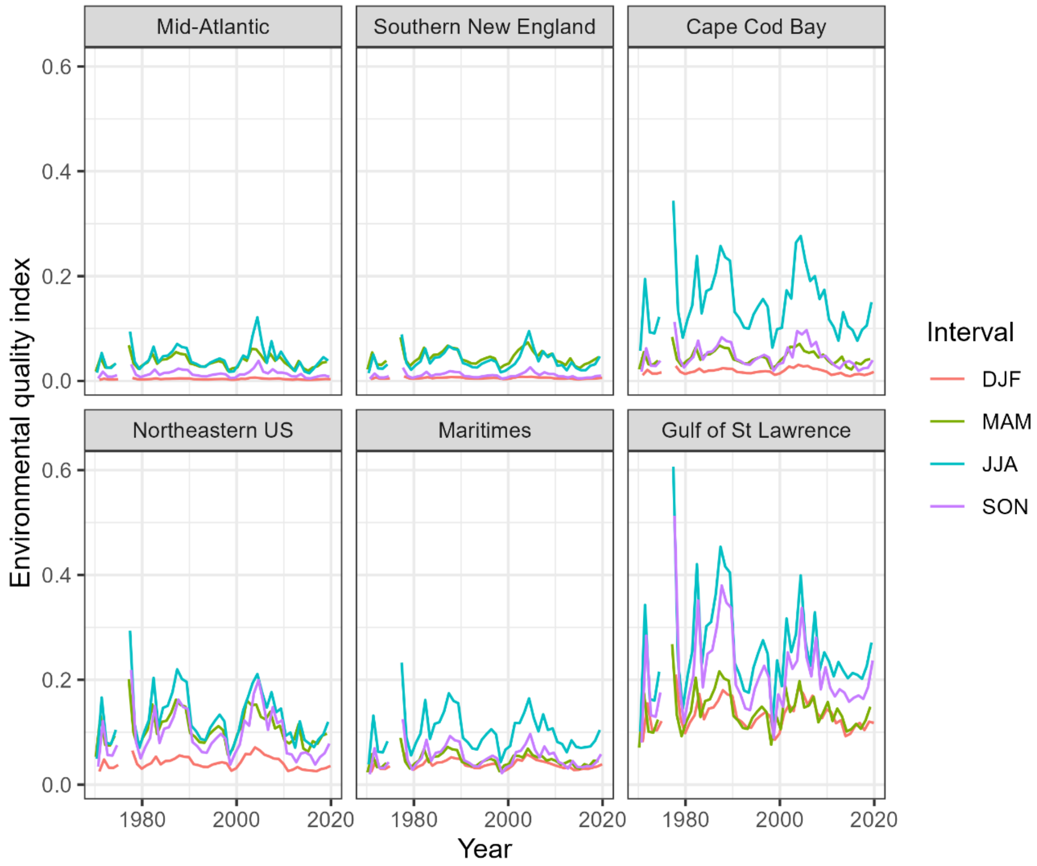

Figure S7. Temporal trend in relative prey conditions per region per three-month interval, as derived from the outputs of the transboundary model for Calanus spp. biomass and the correlation with the continuous Plankton Recorder data. Intervals are indicated using the initial letters of the corresponding months.

Section S6: Model for entanglement events

In the model presented in Pirotta et al. (2023, 2024), the best estimate of the date of occurrence of an entanglement event was calculated prior to fitting the model. For events where the gear was seen attached to the animal, this was inferred from the last sighting of the entangled individual without gear attached, the first sighting with gear, the last sighting with gear, and the first sighting after shedding the gear (see Supplementary material from Pirotta et al. 2023) for details of these calculations). In contrast, for events where only entanglement scarring was observed, entanglement date was estimated from the last sighting of the entangled individual without scarring, and the first sighting with scarring.

Here, we wanted to link the occurrence of entanglement events with the spatially explicit surface describing entanglement risk, with the goal of 1) estimating the scalar ι_1 that converts relative entanglement risk to absolute entanglement probabilities in each region and season (see main text) and 2) estimating putative, unobserved events that may have occurred after the last sighting of individuals (and could have thus contributed to their potential death). Therefore, the estimation of the date of occurrence and duration of each entanglement event was incorporated in the model as follows.

For entanglement events where the gear was observed attached to the body of the animal, we follow Pirotta et al. (2023) and let $E_{i,t}$ represent the entanglement status of individual i in time step t, where $E_{i,t} = 1$ indicates that an individual is entangled and $E_{i,t} = 0$ that it is not. We can write the probability of becoming entangled on a particular time step t'' (event $\dot{e}_{t''}$), given that the individual was not entangled on some previous time step (t') and it did not become entangled in the intervening time steps (i.e., between $t' + 1$ and $t'' - 1$), as:

$$Pr\left(\dot{e}_{t''} \middle| E_{i,t'} = 0\right) = p^e_{i,t''} \prod_{u=t'+1}^{t''-1} \left(1 - p^e_{i,u}\right) \qquad S23$$

where $p^e_{i,t}$ is the probability of becoming entangled on a given time step, which depends on the entanglement risk derived from the Woods Hole Analysis of Line Entanglement Decision Support Tool (WHALE DST; Miller et al. 2024, 2025) for each region and season and the proportion of time spent across regions by individual i in each three-month time step t (see main text).

An animal that is sighted as entangled in time step t'' must have become entangled in that time step or in one of the previous time steps, i.e.:

$$Pr\left(E_{i,t''} = 1 \middle| E_{i,t'} = 0\right) = \sum_{t=t'+1}^{t''} \left(p^e_{i,t} \prod_{u=t'+1}^{t-1} \left(1 - p^e_{i,u}\right) \right). \qquad S24$$

Note that this assumes that an individual could not become entangled, shed the gear, and become entangled again between t' and t''.

The binary occurrence of an observed entanglement event (known and equal to 1) thus emerges from a Bernoulli draw:

$$\ddot{e} \sim Bernoulli\left(Pr\left(E_{i,t''} = 1 \middle| E_{i,t'} = 0\right)\right). \qquad S25$$

The probability of becoming entangled in a specific time step t''', given that the individual was not entangled on t' but it was entangled on t'' (i.e., $t' + 1 \geq t''' \geq t''$), is derived from the probability of getting entangled in that interval (presented above); however, we also know that an event has occurred in that interval (i.e., the probabilities must be scaled to sum to 1), and we must account for the fact that the animal did not shed the gear before t'' (when the gear was observed on the animal):

$$Pr\left(\dot{e}_{t'''} \middle| E_{i,t'} = 0, E_{i,t''} = 1\right) = \frac{p^e_{i,t'''} \prod_{u=t'+1}^{t'''-1}\left(1 - p^e_{i,u}\right) \left(1 - p^d\right)^{t'' - t''' + 1}}{\sum_{t=t'+1}^{t''}\left[p^e_{i,t} \prod_{u=t'+1}^{t-1}\left(1 - p^e_{i,u}\right) \left(1 - p^d\right)^{t'' - t + 1}\right]} \qquad S26$$

where p^d is the probability of shedding the gear. The probabilities in the interval between 1 and t'' can then be used in a categorical draw to determine the starting time step of an entanglement event (i.e., the date when the gear was acquired), t'''.

The probability of an animal becoming unentangled in a given time step w' (event $\dot{u}_{w'}$), given that it was entangled on t''', depends on the probability of shedding the gear (p^d):

$$Pr\left(\dot{u}_{w'} \middle| E_{i,t'''} = 1\right) = p^d\left(1 - p^d\right)^{w' - t'''}. \qquad S27$$

Note that this implies that an individual can shed the gear in the same time step where it became entangled.

We assume that there were no hidden events. Therefore, an animal that is last sighted with gear on time step w'' and as unentangled on time step w' must have kept the gear in the interval between t''' and w'', and shed the gear between w'' and w'. In other words, we can model the binary occurrence of a gear shedding event over two periods, before and after the last sighting with gear:

$$\ddot{u}' \sim Bernoulli\left(\sum_{t=t'''}^{w''} p^d\left(1 - p^d\right)^{t - t'''}\right), \qquad S28$$

$$\ddot{u}'' \sim Bernoulli\left(\sum_{t=w''}^{w'} p^d\left(1 - p^d\right)^{t - w''}\right). \qquad S29$$

where $\ddot{u}' = 0$ and $\ddot{u}'' = 1$.

The probability of becoming unentangled in a specific time step w''', where $t''' \geq w''' \geq w'$, given that the individual was entangled on t''' but it was seen without gear on w', is:

$$Pr\left(u_{w'''} \middle| E_{i,t'''} = 1, E_{i,w'} = 0\right) = \frac{p^d\left(1 - p^d\right)^{w''' - t'''}}{\sum_{t=t'''}^{w'} p^d\left(1 - p^d\right)^{t - t'''}}. \qquad S30$$

The probabilities in the interval between t''' and w' can then be used in a categorical draw to determine the ending time step (i.e., the date when the gear was shed), w''', but with right censoring, in order to account for the fact that the gear stayed attached at least until w''.

This formulation was simplified for:

- Events where the start date of an entanglement event (i.e., the date when the gear was acquired) was known.
- Events where the start date of an entanglement event was unknown but prior to the start of an individual's time series.
- Events where only the scarring was observed (in these cases, both the entanglement and gear shedding events occurred in the interval between the last sighting without the scar, and the first sighting with the scar).
- Open-ended events, where an animal was never re-observed without the gear (in these cases, $\ddot{u}''$ was unknown).
- Events where gear acquisition and shedding occurred within one three-month time step.

Moreover, we modelled the occurrence of a single putative, unobserved event at the end of each individual's time series (in cases where the potential window for occurrence was greater than one time step). These events were modelled in a similar fashion, except that their binary occurrence $\ddot{e}$ was unknown, and the occurrence of the corresponding gear shedding was also unknown and contingent on $\ddot{e}$.

We then used the estimated time steps of gear acquisition and shedding of each event to determine an individual's entanglement state ($E_{i,t}$, where 1 indicates that the individual was entangled, and 0 that it was unentangled). Note that, according to the model formulation described above, entanglement and gear shedding could occur in the same three-month time step. However, we set entanglement state to 1 on the time step where the gear was acquired but not on the time step where the gear was shed (unless the event lasted for a single time step or less), so that we did not double count the uncertainty around the exact entanglement or gear shed dates in a three-month step at both the times of gear acquisition and shedding for an event; this can also be interpreted as $E_{i,t}$ representing an individual's entanglement state at the end of a time step. Also note that, when modelling the effect of entanglement state on health, we distinguished the first time step of an event from any subsequent steps, in order to model its immediate effect separately from its prolonged effect. The severity of an event (minor, moderate or severe) was known for all observed events and was modelled to emerge from a categorical draw with probabilities $p^{s}_{1:3}$, which were then used to impute the severity of the putative unobserved event at the end of an individual's time series.

Finally, in all time steps where no event has or could have occurred, an individual's entanglement state was set to 0 and emerged from a binary draw:

$$E_{i,t} \sim Bernoulli\left(p^{e}_{i,t}\right). \qquad S31$$

Section S7: Model for vessel strike events

There is limited information on the process linking a vessel strike to the detection of the resulting wound and the potential death of the individual. Given this uncertainty, we decided to use a simpler approach to model the occurrence of observed vessel strike events as a function of the underlying risk experienced by an individual (informed from vessel traffic data as described in the main text). Specifically, we did not estimate the time step during which an observed event occurred (between the last sighting without an injury and the first sighting with the injury); allowing an individual to remain alive for multiple time steps after a strike would have diluted the effect over a longer period, with the risk of underestimating the effect on health. Instead, we took a conservative approach and assumed that each event occurred in the time step *t* prior to when it was detected.

For an observed vessel strike event, its occurrence was therefore modelled as:

$$\ddot{v} \sim Bernoulli\left(p_{i,t}^{v}\right) \qquad S32$$

where $\ddot{v}$ is known and equal to 1, and $p_{i,t}^{v}$ is the probability of an individual getting struck given its distribution in that time step (see main text).

We modelled the occurrence of a single putative, unobserved strike event at the end of an individual's time series with probability $\sum_{t=t_i^e}^{T_i} p_{i,t}^{v}\,(1-p_{i,t}^{v})^{t-1}$, where t_i^e is the time of last sighting of an individual, and T_i is the last time step included in the time series (in most cases, corresponding to 2 years after the last sighting). For these unobserved events, $\ddot{v}$ is unknown. Note that we assume that only a single event could have occurred between the last sighting of an individual and the end of its time series.

The type of injury caused by a vessel strike (superficial, shallow, deep, or blunt) was known for all observed events and emerged from a categorical draw with probabilities $p_{1:4}^{w}$, which were then used to impute the type of injury resulting from the putative unobserved event at the end of an individual's time series.

An individual's vessel strike state ($V_{i,t}$) was then set to 1 in the time step where an event was known or estimated to have occurred. In all other time steps, where no event has or could have occurred, $V_{i,t}$ was set to 0 and emerged from a binary draw:

$$V_{i,t} \sim Bernoulli\left(p_{i,t}^{v}\right). \qquad S33$$

This component of the model aimed to link the broad spatio-temporal patterns of risk resulting from vessel traffic (described in section S4) with the occurrence of strike events, with the ultimate goal of estimating the effects of these events on NARW health and vital rates. Therefore, this effort differs from other mechanistic models that describe the fine-scale dynamics and lethality of large-whale vessel strike events (Rockwood et al. 2021, Garrison et al. 2022).

Section S8: Modelling the combined effects of multiple stressors

All prior distributions for the combined effects of stressors discussed in the following sections S8.1-S8.3 are reported in Table S5 (section S10.4).

Section S8.1: Combined effects on health

The baseline equation for the process model of health of individual *i* at time step *t* is described in section S1. The combined effects of multiple stressors on health were then tested in separate models. To assess the combined effect of mean prey conditions and the prolonged effect of carrying the gear from an entanglement event, we added the term: $\beta_{10}\ W_{1,i,t}\ W_{5,i,t}$. In contrast, for the combined effect of mean prey conditions and the immediate effect of an entanglement, we tested the inclusion of three separate terms, each for a given level of severity: $\beta_{10}\ W_{1,i,t}\ W_{2,i,t}$, $\beta_{11}\ W_{1,i,t}\ W_{3,i,t}$, and $\beta_{12}\ W_{1,i,t}\ W_{4,i,t}$. Similarly, to investigate the combined effects of mean prey conditions and vessel strikes, we added one interaction term for each type of injury: $\beta_{10}\ W_{1,i,t}\ W_{6,i,t}$, $\beta_{11}\ W_{1,i,t}\ W_{7,i,t}$, $\beta_{12}\ W_{1,i,t}\ W_{8,i,t}$, and $\beta_{13}\ W_{1,i,t}\ W_{9,i,t}$.

To investigate the cumulative effects of repeated entanglement events, we included the additional term $\beta_{10}\ W_{10,i,t}\ \max(W_{2,i,t}, W_{3,i,t}, W_{4,i,t})$, which applied whenever an entanglement event started (i.e., when an animal was estimated to have acquired the gear, and thus any term among $W_{2,i,t}$, $W_{3,i,t}$, and $W_{4,i,t}$ was equal to 1). $W_{10,i,t}$ was either the cumulative number of entanglement events an individual experienced prior to the current event, the binary occurrence of any event prior to the current one, or the binary occurrence of an event in the 2 years prior to the current one, depending on the hypothesis being tested (see Table 1 in the main text).

Section S8.2: Combined effects on length

We investigated the combined effects of stressors on growth by modifying the equation for the individual-specific asymptote of length. Following Pirotta et al. (2024), the individual-specific length asymptote A_i was modelled as a function of an individual's birth year (B_i):

$$A_i = A + \upsilon_1\ B_i + \varepsilon_i \qquad S34$$

where A is the intercept, υ_1 is the effect of birth year and ε_i is a normally distributed random effect with mean 0 and standard deviation σ_A. B_i was rescaled as $max(B_i - 1977, 0)$, so $B_i = 0$ when an individual was born prior to or in 1977 (the first year where a birth event was recorded). Therefore, A represented mean asymptotic length for animals born until 1977.

To avoid excessive imputation of missing covariates for animals of unknown birth year, the length model above was only modified for animals of known age, where we replaced the effect of B_i with putative explanatory variables. For individuals of unknown age, we kept the model including the effect of estimated birth year B_i. Note that we estimated two separate intercepts for the asymptote models for animals of known and unknown age, A_1 and A_2.

First, we investigated the effect of the mean health state over an increasing time window from the start of an individual's life (1, 2, 5, 10, and 15 y), substituting the term $\upsilon_1\, B_i$ with $\upsilon_2\, \overline{H}_i$, where $\overline{H}_i$ indicates the mean health state in the window under consideration. In the two models testing the effect of the mother's health on calf growth (Table 1), we instead included the term $\upsilon_2\, H_i^m$, where H_i^m indicates either the mother's health state at the start of the lactation year, or her mean health during the lactation year.

For the combined effects of mean prey conditions ($\overline{P}_i$) and mean entanglement status ($\overline{E}_i$, capturing the proportion of time a growing individual was entangled, calculated from the estimated entanglement state of an individual) over a temporal window of increasing duration (1, 2, 5, 10, and 15 y), we modified the model for the asymptote of a known-age individual to:

$$A_i = A_2 + \upsilon_2\, \overline{P}_i + \upsilon_3\, \overline{E}_i + \upsilon_4\, \overline{P}_i\overline{E}_i + \varepsilon_i. \qquad S35$$

Finally, we assessed the potential effect of the mother's entanglement status during the lactation year ($\overline{E}_i^m$), by including the term $\upsilon_2\, \overline{E}_i^m$.

Section S8.3: Combined effects on exposure

The changes in exposure to entanglement and vessel strike risk resulting from the shift in NARW distribution from 2010 onwards were assessed by investigating the spatio-temporal trends in entanglement and vessel strike probability (see section S11). Moreover, we assessed whether the average prey conditions in a year affected the probability of getting entangled or vessel struck. Specifically, for the effect of mean prey conditions ($W_{1,i,t}$) on entanglement risk, we modified the corresponding equation (see equation 1 in the main text) to:

$$p_{i,t}^e = \iota_1 \sum_{l=1}^{R} z_{l,i,t}\, G_{l,t}\, \tilde{e}_{l,t} + \beta_{10}\, W_{1,i,t}. \qquad S36$$

For the effect of mean prey conditions on vessel strike risk, we modified the corresponding equations (see equations 2 and 3 in the main text) to:

$$logit(p_{i,t}^v) = \iota_2 \sum_{l=1}^{R} z_{l,i,t}\, AIS_{l,t} + \tilde{\upsilon}\,(2019 - y_t) + \beta_{10}\, W_{1,i,t}. \qquad S37$$

Section S9: Model notation

Table S1. Symbols used for variables, parameters, and scalars across the three formulations of the model.

Symbol	Model version	Description
i	All	Individual whale
N	All	Total number of individuals
t	All	Three-month time step
T_i	All	Total number of time steps for an individual
$h_{i,t}$	All	Latent health status
j	All	Index for intrinsic stressors
$Z_{j,i,t}$	All	Intrinsic stressors
k	All	Index for extrinsic stressors
$W_{k,i,t}$	All	Extrinsic stressors
α_1	All	Effect of juvenile status
α_2	All	Effect of lactation status
β_1	All	Effect of prey abundance index on health in Jun-Aug interval
β_2	All	Immediate effect of minor entanglements
β_3	All	Immediate effect of moderate entanglements
β_4	All	Immediate effect of severe entanglements
β_5	All	Prolonged effect of carrying fishing gear
β_6	All	Effect of vessel strikes (superficial injury)
β_7	All	Effect of vessel strikes (shallow injury)
β_8	All	Effect of vessel strikes (deep injury)
β_9	All	Effect of vessel strikes (blunt injury)
$\vartheta_{i,t}$	All	Survival probability
$s_{i,t}$	All	Survival state (alive or dead)
σ	All	Process standard deviation of health
x	All	Ordinal variable comprising the VHA
$\eta_{i,t,x,1}$	All	Probability of observing the first category of each VHA variable
$\eta_{i,t,x,2}$	All	Probability of observing the second category of each VHA variable
$\eta_{i,t,x,3}$	All	Probability of observing the third category of each VHA variable (where applicable)
$Q_{i,t,x}$	All	Cumulative probabilities used in the ordinal logit model for three-category VHA variables
$\gamma_{1,1}$	All	First intercept of the observation model for body condition
$\gamma_{1,2}$	All	Second intercept of the observation model for body condition
$\gamma_{2,1}$	All	Intercept of the observation model for skin condition
$\gamma_{3,1}$	All	First intercept of the observation model for rake marks
$\gamma_{3,2}$	All	Second intercept of the observation model for rake marks
$\gamma_{4,1}$	All	Intercept of the observation model for cyamid presence
ω	All	Effect of health on VHA observations
$o_{i,t,x,1:O_{i,t,x}}$	All	Ordinal observations of a VHA variable

$O_{i,t,x}$	All	Number of observations for a VHA variable in a time step
ρ	All	Sighting probability over a three-month interval
$p_{i,t}$	All	Probability of sighting an individual alive in a time step
$e_{i,t}$	All	Individual sighting
t_i^e	All	Time of last sighting of an individual
y	All	Year when a female is available to give birth
$\varphi_{i,y}$	All	Calving probability for a female in a given year
$t(y)$	All	Time step prior to a reproductive attempt identifying the health and length that can affect calving probability
$m_{i,y}$	All	Asymptote of the sigmoid relationship representing the maximum calving probability for a female in a given year
M_i	All	Individual-specific intercept of asymptotic calving probability
g_i	All	Indicator of whether a female is reproductive (i.e., has already calved or may do so in the future)
δ	All	Steepness of sigmoid relationship between health and calving probability
μ	All	Value of health at which calving probability is 50% of maximum
ν	All	Mean probability of belonging to the reproductive group
λ	All	Intercept of asymptote of calving probability
χ	All	Standard deviation of individual random effect on asymptote of calving probability
ζ	All	Effect of cube length on asymptote of calving probability
$r_{i,y}$	All	Calving state (female has calved or not)
$L_{i,t}$	All	True body length
A	All	Intercept of length asymptote
A_i	All	Individual-specific length asymptote
K	All	Growth rate
C	All	Displacement along x-axis (Gompertz curve)
υ_1	All	Effect of birth year on length asymptote
ε_i	All	Individual random effect on length asymptote
σ_A	All	Standard deviation of individual random effect on length asymptote
$a_{i,t}$	All	Age of an individual
B_i	All	Birth year of an individual
$P_{i,t}$	All	Observed body length
ξ_1	All	Altitude observation error (GPS)
ξ_2	All	Altitude observation error (Laser)
ξ_3	All	Altitude observation error (Radar)
l	v1, v2	Regional polygon
R	v1, v2	Total number of regional polygons
$z_{t,i,1:R}$	v1, v2	Proportion of time spent across regional polygons
d, also expressed as a function of time step $d(t)$, or sighting $d(e)$	v1, v2	Period (prior to 2010, from 2010 onwards)
q, also expressed as a function of time step $q(t)$, or sighting $q(e)$	v1, v2	Season (Dec-Feb, Mar-May, Jun-Aug, Sep-Nov)
c', also expressed as a function of individual and time step $c'(i,t)$	v1	Age class (adult, juvenile, calf)

f, also expressed as a function of individual $f(i)$	v1	Sex (male, female)
$\pi_{d(t),c'(i,t),f(i),q(t),1:R}$	v1	Parameters of the Dirichlet distribution for individual proportional occurrence in different regions
$a'_{q,1:R}$	v1	Parameters for the population's average spatial distribution
c'', also expressed as a function of individual and time step $c''(i,t)$, or individual and sighting $c''(i,e)$	v2	Age and sex class (calves, juveniles, adult females, adult males)
$\tilde{l}_{t,i,1:R}$	v2	Expected spatial distribution of the average individual
$\tilde{p}_{d(t),q(t),c''(i,t),1:R}$	v2	Expected proportion of animals of a given age and sex class across regions
$a''_{d(t),q(t),1:R}$	v2	Parameters of the Dirichlet distributions fitted to NARW proportional abundance across regional polygons (given the season and period) derived from 200 realisations of the density surface model
$E_{i,t}$	All	Individual entanglement state
$G_{l,t}$	v1, v2	Value of the entanglement risk surface for a given region and time step
$\tilde{e}_{l,t}$	v1, v2	Scalar derived from the landings data to extrapolate risk to years prior to 2015
ι_1	v1, v2	Scalar converting entanglement risk into a probability
$p^e_{i,t}$	v1, v2	Entanglement probability in a time step
$\dot{e}_t$	v1, v2	Event of becoming entangled in a given time step
$\ddot{e}$	v1, v2	Binary occurrence of an entanglement event
$\dot{u}_t$	v1, v2	Event of shedding the gear in a given time step
$\ddot{u}$	v1, v2	Binary occurrence of gear shedding
t'	v1, v2	Last sighting without gear
t''	v1, v2	First sighting with gear
t'''	v1, v2	Beginning of entanglement event (gear acquisition)
w'	v1, v2	First sighting without gear
w''	v1, v2	Last sighting with gear
w'''	v1, v2	End of entanglement event (gear shedding)
p^d	v1, v2	Probability of shedding the gear
p^s_1	v1, v2	Proportion of minor entanglements
p^s_2	v1, v2	Proportion of moderate entanglements
p^s_3	v1, v2	Proportion of severe entanglements
$V_{i,t}$	All	Individual vessel strike state
$AIS_{l,t}$	v1, v2	Value of the vessel strike risk surface for a regional polygon and time step
ι_2	v1, v2	Scalar converting vessel strike risk into a probability
$\tilde{v}$	v1, v2	Temporal trend in strike probability
$p^v_{i,t}$	v1, v2	Vessel strike probability for an individual in a time step
$p^r_{l,t}$	v1, v2	Vessel strike probability in a region and time step
$\ddot{v}$	v1, v2	Binary occurrence of a vessel strike event
y_t	v1, v2	Corresponding year for a given time step
p^w_1	v1, v2	Proportion of vessel strikes causing a superficial injury
p^w_2	v1, v2	Proportion of vessel strikes causing a shallow injury

p_3^w	v1, v2	Proportion of vessel strikes causing a deep injury
p_4^w	v1, v2	Proportion of vessel strikes causing a blunt injury
$prey_{l,t}$	v1, v2	Index of prey conditions in a region and time step
$prey'_{i,t}$	v1, v2	Index of prey conditions in a time step weighted by an individual's distribution
$DSM_{1:R,t}$	v1, v2	NARW density across region (only used for model schematic in Fig. 2)
$\beta_{10:13}$	All	Combined effects of stressors on health (see Table S5 for details)
$\upsilon_{2:4}$	All	Combined effects of stressors on length asymptote (see Table S5 for details)
A_1, A_2	All	Separate intercepts for animals of known and unknown age when assessing the combined effects of stressors on length asymptote
$\bar{H}_i$	v3	Mean health state over a variable window at the start of an individual's life
H_i^m	All	Mother's health status at the start of the lactation year, or her mean health during the lactation year
$\bar{P}_i$	All	Mean prey conditions over a variable window at the start of an individual's life
$\bar{E}_i$	All	Mean entanglement status over a variable window at the start of an individual's life
$\bar{E}_i^m$	All	Mother's entanglement status during the lactation year

Section S10: Prior and posterior distributions of model parameters across the three model formulations

Section S10.1: Spatial model (data-driven formulation, v1)

Table S2. Prior distribution and posterior mean, standard deviation (SD), and 2.5th, 50th (median) and 97.5th quantiles of model parameters. Note that the normal distribution is parameterised here as Normal(mean, standard deviation). The last column reports the proportion overlap between the prior and posterior distributions.

Parameter	Symbol	Prior	Posterior					Overlap prior-posterior
			Mean	*SD*	*2.5%*	*50%*	*97.5%*	
First intercept of the observation model for body condition	$\gamma_{1,1}$	*Normal*(5, 5)	2.08	0.28	1.53	2.08	2.63	0.11
Second intercept of the observation model for body condition	$\gamma_{1,2}$	*Normal*(5, 5) $\gamma_{1,2} > \gamma_{1,1}$	8.44	0.23	7.99	8.43	8.89	0.09
Intercept of the observation model for skin condition	$\gamma_{2,1}$	*Normal*(5, 5)	7.25	0.22	6.82	7.25	7.69	0.09
First intercept of the observation model for rake marks	$\gamma_{3,1}$	*Normal*(5, 5)	3.25	0.23	2.78	3.25	3.71	0.1
Second intercept of the observation model for rake marks	$\gamma_{3,2}$	*Normal*(5, 5) $\gamma_{3,2} > \gamma_{3,1}$	7.55	0.22	7.12	7.55	7.99	0.1
Intercept of the observation model for cyamid presence	$\gamma_{4,1}$	*Normal*(5, 5)	4.41	0.22	3.98	4.41	4.86	0.1
Effect of health on VHA observations	ω	*Normal*(-5, 2)	-5.58	0.13	-5.84	-5.58	-5.31	0.14
Effect of juvenile status	α_1	*Normal*(0, 1)	-0.14	0.02	-0.18	-0.14	-0.10	0.05
Effect of lactation	α_2	*Normal*(0, 1)	-0.04	0.00	-0.05	-0.04	-0.03	0.02
Effect of prey abundance index on health in Jun-Aug interval	β_1	*Normal*(0, 1)	0.03	0.01	0.01	0.03	0.05	0.03
Immediate effect of minor entanglements	β_2	*Normal*(0, 1)	-0.03	0.01	-0.05	-0.03	-0.01	0.03
Immediate effect of moderate entanglements	β_3	*Normal*(0, 1)	-0.04	0.01	-0.07	-0.04	-0.02	0.04
Immediate effect of severe entanglements	β_4	*Normal*(0, 1)	-0.52	0.03	-0.59	-0.52	-0.45	0.07

Prolonged effect of carrying fishing gear	β_5	*Normal*(0, 1)	-0.02	0.01	-0.04	-0.02	0.00	0.02
Effect of vessel strikes (superficial injury)	β_6	*Normal*(0, 1)	0.06	0.08	-0.09	0.06	0.21	0.16
Effect of vessel strikes (shallow injury)	β_7	*Normal*(0, 1)	-0.22	0.09	-0.39	-0.23	-0.05	0.18
Effect of vessel strikes (deep injury)	β_8	*Normal*(0, 1)	-0.38	0.08	-0.52	-0.37	-0.23	0.15
Effect of vessel strikes (blunt injury)	β_9	*Normal*(0, 1)	-0.92	0.12	-1.15	-0.92	-0.69	0.16
Sighting probability over a three-month interval	ρ	*Uniform*(0, 1)	0.37	0.00	0.37	0.37	0.38	0.02
Intercept of length asymptote	A	*Normal*(13.82, 0.28)*	13.99	0.11	13.78	13.99	14.21	0.49
Growth rate	K	*Normal*(0.13, 0.03)*	0.18	0.02	0.15	0.18	0.22	0.25
Displacement along x-axis (Gompertz curve)	C	*Normal*(0.33, 0.02)*	0.29	0.01	0.26	0.29	0.31	0.19
Effect of birth year on length asymptote	υ_1	*Normal*(0, 1)	-0.05	0.01	-0.06	-0.05	-0.03	0.02
Standard deviation of individual random effect on length asymptote	σ_A	*Uniform*(0, 10)	0.51	0.05	0.41	0.50	0.62	0.03
Steepness of sigmoid relationship between health and calving probability	δ	*Normal*(15, 5)	19.41	3.98	12.33	19.25	27.84	0.62
Value of health at which calving probability is 50% of maximum	μ	*Normal*(1, 1)	1.37	0.03	1.29	1.37	1.43	0.07
Mean probability of belonging to the reproductive group	v	*Uniform*(0, 1)	0.93	0.02	0.89	0.93	0.97	0.12
Intercept of asymptote of calving probability	λ	*Normal*(0, 1)	-0.86	0.12	-1.10	-0.86	-0.63	0.17
Standard deviation of individual random effect on asymptote of calving probability	χ	*Uniform*(0, 10)	0.58	0.14	0.29	0.58	0.85	0.08
Effect of cube length on asymptote of calving probability	ζ	*Normal*(0, 1)	0.76	0.12	0.53	0.76	1.00	0.2
Altitude observation error (GPS)	ξ_1	*Uniform*(0, 10)	0.66	0.15	0.41	0.64	0.98	0.08
Altitude observation error (Laser)	ξ_2	*Uniform*(0, 10)	0.69	0.10	0.51	0.69	0.91	0.06
Altitude observation error (Radar)	ξ_3	*Uniform*(0, 10)	0.28	0.03	0.23	0.27	0.34	0.02

Scalar converting entanglement risk into a probability	ι_1	*Uniform*(0, 1)	0.50	0.01	0.48	0.50	0.53	0.07
Scalar converting vessel strike risk into a probability	ι_2	*Uniform*(0, 1)	0.02	0.00	0.01	0.02	0.02	0.02
Probability of shedding the gear	p^d	*Uniform*(0, 1)	0.45	0.01	0.43	0.45	0.48	0.07
Temporal trend in strike probability	$\tilde{v}$	*Normal*(0, 3)	-0.03	0.01	-0.06	-0.03	-0.01	0.01
Proportion of minor entanglements	p_1^s		0.75	0.01	0.73	0.75	0.77	0.18
Proportion of moderate entanglements	p_2^s	*Dirichlet*(8, 2, 1)	0.16	0.01	0.14	0.16	0.18	0.18
Proportion of severe entanglements	p_3^s		0.09	0.01	0.08	0.09	0.11	0.17
Proportion of vessel strikes causing a superficial injury	p_1^w		0.29	0.05	0.20	0.29	0.40	0.52
Proportion of vessel strikes causing a shallow injury	p_2^w	*Dirichlet*(3, 2, 3, 2)	0.17	0.04	0.10	0.17	0.26	0.52
Proportion of vessel strikes causing a deep injury	p_3^w		0.30	0.05	0.21	0.30	0.40	0.53
Proportion of vessel strikes causing a blunt injury	p_4^w		0.24	0.05	0.15	0.23	0.33	0.5

* priors from Stewart et al. (2021).

The parameters for the population's average spatial distribution in a three-month interval q and location l (see section S2.1) had priors $a'_{q,l} \sim Gamma(\text{shape} = 1, \text{rate} = 1)$, while the posteriors are not reported for brevity.

Section S10.2: Spatial model (model-based formulation, v2)

Table S3. Prior distribution and posterior mean, standard deviation (SD), and 2.5th, 50th (median) and 97.5th quantiles of model parameters. Note that the normal distribution is parameterised here as Normal(mean, standard deviation). The last column reports the proportion overlap between the prior and posterior distributions.

Parameter	Symbol	Prior	Posterior					Overlap prior-posterior
			Mean	*SD*	*2.5%*	*50%*	*97.5%*	
First intercept of the observation model for body condition	$\gamma_{1,1}$	*Normal*(5, 5)	2.10	0.27	1.57	2.10	2.63	0.1
Second intercept of the observation model for body condition	$\gamma_{1,2}$	*Normal*(5, 5) $\gamma_{1,2} > \gamma_{1,1}$	8.46	0.22	8.04	8.46	8.91	0.08
Intercept of the observation model for skin condition	$\gamma_{2,1}$	*Normal*(5, 5)	7.28	0.22	6.87	7.27	7.70	0.09
First intercept of the observation model for rake marks	$\gamma_{3,1}$	*Normal*(5, 5)	3.28	0.23	2.83	3.27	3.74	0.09
Second intercept of the observation model for rake marks	$\gamma_{3,2}$	*Normal*(5, 5) $\gamma_{3,2} > \gamma_{3,1}$	7.58	0.22	7.16	7.57	8.01	0.09
Intercept of the observation model for cyamid presence	$\gamma_{4,1}$	*Normal*(5, 5)	4.44	0.22	4.03	4.43	4.87	0.1
Effect of health on VHA observations	ω	*Normal*(-5, 2)	-5.59	0.13	-5.85	-5.59	-5.35	0.13
Effect of juvenile status	α_1	*Normal*(0, 1)	-0.14	0.02	-0.18	-0.14	-0.10	0.05
Effect of lactation	α_2	*Normal*(0, 1)	-0.04	0.00	-0.05	-0.04	-0.03	0.02
Effect of prey abundance index on health in Jun-Aug interval	β_1	*Normal*(0, 1)	0.03	0.01	0.01	0.03	0.05	0.02
Immediate effect of minor entanglements	β_2	*Normal*(0, 1)	-0.03	0.01	-0.05	-0.03	-0.01	0.03
Immediate effect of moderate entanglements	β_3	*Normal*(0, 1)	-0.05	0.01	-0.08	-0.05	-0.02	0.04
Immediate effect of severe entanglements	β_4	*Normal*(0, 1)	-0.54	0.03	-0.61	-0.54	-0.47	0.07
Prolonged effect of carrying fishing gear	β_5	*Normal*(0, 1)	-0.02	0.01	-0.03	-0.02	0.00	0.02
Effect of vessel strikes (superficial injury)	β_6	*Normal*(0, 1)	0.06	0.08	-0.09	0.06	0.22	0.16

Effect of vessel strikes (shallow injury)	β_7	*Normal*(0, 1)	-0.23	0.08	-0.38	-0.23	-0.05	0.18
Effect of vessel strikes (deep injury)	β_8	*Normal*(0, 1)	-0.38	0.08	-0.53	-0.37	-0.23	0.15
Effect of vessel strikes (blunt injury)	β_9	*Normal*(0, 1)	-0.88	0.12	-1.11	-0.88	-0.65	0.18
Sighting probability over a three-month interval	ρ	*Uniform*(0, 1)	0.37	0.00	0.37	0.37	0.38	0.02
Intercept of length asymptote	A	*Normal*(13.82, 0.28)*	13.99	0.11	13.78	13.99	14.21	0.49
Growth rate	K	*Normal*(0.13, 0.03)*	0.18	0.02	0.15	0.18	0.22	0.26
Displacement along x-axis (Gompertz curve)	C	*Normal*(0.33, 0.02)*	0.29	0.01	0.26	0.29	0.31	0.18
Effect of birth year on length asymptote	υ_1	*Normal*(0, 1)	-0.05	0.01	-0.06	-0.05	-0.03	0.02
Standard deviation of individual random effect on length asymptote	σ_A	*Uniform*(0, 10)	0.51	0.05	0.41	0.50	0.61	0.03
Steepness of sigmoid relationship between health and calving probability	δ	*Normal*(15, 5)	19.24	3.88	12.17	19.06	27.33	0.64
Value of health at which calving probability is 50% of maximum	μ	*Normal*(1, 1)	1.36	0.03	1.29	1.37	1.43	0.08
Mean probability of belonging to the reproductive group	ν	*Uniform*(0, 1)	0.93	0.02	0.88	0.93	0.97	0.11
Intercept of asymptote of calving probability	λ	*Normal*(0, 1)	-0.86	0.12	-1.10	-0.86	-0.64	0.18
Standard deviation of individual random effect on asymptote of calving probability	χ	*Uniform*(0, 10)	0.58	0.14	0.29	0.57	0.85	0.08
Effect of cube length on asymptote of calving probability	ζ	*Normal*(0, 1)	0.76	0.12	0.52	0.76	1.01	0.18
Altitude observation error (GPS)	ξ_1	*Uniform*(0, 10)	0.67	0.15	0.42	0.65	0.99	0.08
Altitude observation error (Laser)	ξ_2	*Uniform*(0, 10)	0.69	0.10	0.51	0.69	0.90	0.06
Altitude observation error (Radar)	ξ_3	*Uniform*(0, 10)	0.28	0.03	0.23	0.28	0.35	0.02
Scalar converting entanglement risk into a probability	ι_1	*Uniform*(0, 1)	0.71	0.02	0.68	0.71	0.75	0.1

Scalar converting vessel strike risk into a probability	ι_2	*Uniform*(0, 1)	0.02	0.00	0.01	0.02	0.02	0.02
Probability of shedding the gear	p^d	*Uniform*(0, 1)	0.45	0.01	0.43	0.45	0.47	0.07
Temporal trend in strike probability	$\tilde{v}$	*Normal*(0, 3)	-0.02	0.01	-0.05	-0.02	0.00	0.02
Proportion of minor entanglements	p_1^s	*Dirichlet*(8, 2, 1)	0.75	0.01	0.73	0.75	0.77	0.17
Proportion of moderate entanglements	p_2^s		0.16	0.01	0.14	0.16	0.18	0.17
Proportion of severe entanglements	p_3^s		0.09	0.01	0.08	0.09	0.11	0.17
Proportion of vessel strikes causing a superficial injury	p_1^w	*Dirichlet*(3, 2, 3, 2)	0.29	0.05	0.20	0.29	0.40	0.53
Proportion of vessel strikes causing a shallow injury	p_2^w		0.17	0.04	0.10	0.17	0.26	0.52
Proportion of vessel strikes causing a deep injury	p_3^w		0.30	0.05	0.21	0.30	0.40	0.52
Proportion of vessel strikes causing a blunt injury	p_4^w		0.23	0.05	0.14	0.23	0.33	0.49

* priors from Stewart et al. (2021).

The proportion of individuals in each demographic class in a given region l in a three-month interval q in period d (see section S2.2) had priors $\tilde{p}_{d,q,1:4,l} \sim Dirichlet(1,1,1,1)$, while the posteriors are not reported for brevity.

Section S10.3: Non-spatial model (Pirotta et al. 2024, v3)

Table S4. Prior distribution and posterior mean, standard deviation (SD), and 2.5th, 50th (median) and 97.5th quantiles of model parameters. Note that the normal distribution is parameterised here as Normal(mean, standard deviation). The last column reports the proportion overlap between the prior and posterior distributions.

Parameter	Symbol	Prior	Posterior					Overlap prior-posterior
			Mean	*SD*	*2.5%*	*50%*	*97.5%*	
First intercept of the observation model for body condition	$\gamma_{1,1}$	*Normal*(5, 5)	2.19	0.28	1.65	2.20	2.71	0.11
Second intercept of the observation model for body condition	$\gamma_{1,2}$	*Normal*(5, 5)<br>$\gamma_{1,2} > \gamma_{1,1}$	8.52	0.22	8.11	8.52	8.97	0.08
Intercept of the observation model for skin condition	$\gamma_{2,1}$	*Normal*(5, 5)	7.33	0.22	6.93	7.33	7.78	0.09
First intercept of the observation model for rake marks	$\gamma_{3,1}$	*Normal*(5, 5)	3.36	0.23	2.93	3.35	3.81	0.1
Second intercept of the observation model for rake marks	$\gamma_{3,2}$	*Normal*(5, 5)<br>$\gamma_{3,2} > \gamma_{3,1}$	7.63	0.22	7.23	7.63	8.08	0.09
Intercept of the observation model for cyamid presence	$\gamma_{4,1}$	*Normal*(5, 5)	4.50	0.22	4.09	4.50	4.95	0.1
Effect of health on VHA observations	ω	*Normal*(-5, 2)	-5.62	0.13	-5.87	-5.61	-5.37	0.14
Effect of juvenile status	α_1	*Normal*(0, 1)	-0.15	0.02	-0.19	-0.15	-0.11	0.05
Effect of lactation	α_2	*Normal*(0, 1)	-0.04	0.00	-0.05	-0.04	-0.03	0.02
Effect of prey abundance index on health in Jun-Aug interval	β_1	*Normal*(0, 1)	0.03	0.01	0.02	0.03	0.05	0.02
Immediate effect of minor entanglements	β_2	*Normal*(0, 1)	-0.01	0.01	-0.03	-0.01	0.01	0.03
Immediate effect of moderate entanglements	β_3	*Normal*(0, 1)	0.00	0.02	-0.04	0.00	0.04	0.06
Immediate effect of severe entanglements	β_4	*Normal*(0, 1)	-0.49	0.03	-0.55	-0.48	-0.42	0.07
Prolonged effect of carrying fishing gear	β_5	*Normal*(0, 1)	-0.02	0.01	-0.03	-0.02	0.00	0.16
Effect of vessel strikes (superficial injury)	β_6	*Normal*(0, 1)	-0.01	0.07	-0.15	0.00	0.13	0.15

Effect of vessel strikes (shallow injury)	β_7	*Normal*(0, 1)	-0.22	0.09	-0.40	-0.22	-0.04	0.19
Effect of vessel strikes (deep injury)	β_8	*Normal*(0, 1)	-0.45	0.08	-0.61	-0.45	-0.29	0.15
Effect of vessel strikes (blunt injury)	β_9	*Normal*(0, 1)	-1.06	0.14	-1.34	-1.06	-0.78	0.02
Sighting probability over a three-month interval	ρ	*Uniform*(0, 1)	0.37	0.00	0.37	0.37	0.38	0.02
Intercept of length asymptote	A	*Normal*(13.82, 0.28)*	13.99	0.11	13.78	13.99	14.22	0.51
Growth rate	K	*Normal*(0.13, 0.03)*	0.18	0.02	0.15	0.18	0.22	0.25
Displacement along x-axis (Gompertz curve)	C	*Normal*(0.33, 0.02)*	0.29	0.01	0.26	0.29	0.31	0.18
Effect of birth year on length asymptote	υ_1	*Normal*(0, 1)	-0.05	0.01	-0.06	-0.04	-0.03	0.02
Standard deviation of individual random effect on length asymptote	σ_A	*Uniform*(0, 10)	0.51	0.05	0.41	0.51	0.61	0.03
Steepness of sigmoid relationship between health and calving probability	δ	*Normal*(15, 5)	19.33	3.96	12.28	19.15	27.57	0.63
Value of health at which calving probability is 50% of maximum	μ	*Normal*(1, 1)	1.37	0.03	1.30	1.37	1.43	0.08
Mean probability of belonging to the reproductive group	ν	*Uniform*(0, 1)	0.93	0.02	0.88	0.93	0.97	0.11
Intercept of asymptote of calving probability	λ	*Normal*(0, 1)	-0.87	0.12	-1.10	-0.87	-0.63	0.18
Standard deviation of individual random effect on asymptote of calving probability	χ	*Uniform*(0, 10)	0.57	0.15	0.22	0.57	0.86	0.08
Effect of cube length on asymptote of calving probability	ζ	*Normal*(0, 1)	0.76	0.13	0.52	0.76	1.02	0.19
Altitude observation error (GPS)	ξ_1	*Uniform*(0, 10)	0.66	0.15	0.41	0.65	0.97	0.08
Altitude observation error (Laser)	ξ_2	*Uniform*(0, 10)	0.69	0.10	0.50	0.69	0.90	0.06
Altitude observation error (Radar)	ξ_3	*Uniform*(0, 10)	0.28	0.03	0.23	0.28	0.34	0.02

* priors from Stewart et al. (2021).

Section S10.4: Combined effects (across model formulations) as defined in section S8

Table S5. Prior distribution and posterior 2.5th, 50th (median) and 97.5th quantiles of model parameters. Note that the normal distribution is parameterised here as Normal(mean, standard deviation). The last column reports the proportion overlap between the prior and posterior distributions.

**Level**	**Combined effect**	**Formulation**	**Parameter**	**Prior**	**Posterior**			**Overlap prior-posterior**
					2.5%	*50%*	*97.5%*	
Health	Prey × entanglement (prolonged)	Data-driven (v1)	β_{10}	*Normal*(0, 1)	0.00	0.08	0.16	0.09
Health	Prey × entanglement (prolonged)	Model-based (v2)	β_{10}	*Normal*(0, 1)	0.01	0.08	0.15	0.08
Health	Prey × entanglement (prolonged)	Non-spatial (v3)	β_{10}	*Normal*(0, 1)	-0.02	0.01	0.04	0.04
Health	Prey × entanglement (immediate, minor)	Data-driven (v1)	β_{10}	*Normal*(0, 1)	-0.13	-0.04	0.06	0.11
Health	Prey × entanglement (immediate, moderate)	Data-driven (v1)	β_{11}	*Normal*(0, 1)	-0.01	0.16	0.33	0.18
Health	Prey × entanglement (immediate, severe)	Data-driven (v1)	β_{12}	*Normal*(0, 1)	0.08	0.39	0.71	0.28
Health	Prey × entanglement (immediate, minor)	Model-based (v2)	β_{10}	*Normal*(0, 1)	-0.16	-0.05	0.05	0.12
Health	Prey × entanglement (immediate, moderate)	Model-based (v2)	β_{11}	*Normal*(0, 1)	-0.10	0.11	0.31	0.2
Health	Prey × entanglement (immediate, severe)	Model-based (v2)	β_{12}	*Normal*(0, 1)	-0.56	-0.10	0.38	0.41
Health	Prey × entanglement (immediate, minor)	Non-spatial (v3)	β_{10}	*Normal*(0, 1)	-0.12	-0.06	-0.01	0.07
Health	Prey × entanglement (immediate, moderate)	Non-spatial (v3)	β_{11}	*Normal*(0, 1)	-0.05	0.06	0.17	0.12
Health	Prey × entanglement (immediate, severe)	Non-spatial (v3)	β_{12}	*Normal*(0, 1)	-0.16	-0.01	0.16	0.17

Health	Prey × vessel strike (superficial)	Data-driven (v1)	β_{10}	*Normal*(0, 1)	-0.78	-0.12	0.52	0.51
Health	Prey × vessel strike (shallow)	Data-driven (v1)	β_{11}	*Normal*(0, 1)	-1.15	-0.07	0.80	0.66
Health	Prey × vessel strike (deep)	Data-driven (v1)	β_{12}	*Normal*(0, 1)	-1.30	-0.68	-0.11	0.4
Health	Prey × vessel strike (blunt)	Data-driven (v1)	β_{13}	*Normal*(0, 1)	-1.05	-0.19	0.57	0.59
Health	Prey × vessel strike (superficial)	Model-based (v2)	β_{10}	*Normal*(0, 1)	-0.57	0.19	0.93	0.56
Health	Prey × vessel strike (shallow)	Model-based (v2)	β_{11}	*Normal*(0, 1)	-0.97	-0.07	0.75	0.61
Health	Prey × vessel strike (deep)	Model-based (v2)	β_{12}	*Normal*(0, 1)	-1.10	-0.50	0.11	0.44
Health	Prey × vessel strike (blunt)	Model-based (v2)	β_{13}	*Normal*(0, 1)	-0.86	0.01	0.92	0.63
Health	Prey × vessel strike (superficial)	Non-spatial (v3)	β_{10}	*Normal*(0, 1)	-0.32	0.09	0.52	0.38
Health	Prey × vessel strike (shallow)	Non-spatial (v3)	β_{11}	*Normal*(0, 1)	-0.82	-0.13	0.53	0.53
Health	Prey × vessel strike (deep)	Non-spatial (v3)	β_{12}	*Normal*(0, 1)	-1.00	-0.59	-0.16	0.32
Health	Prey × vessel strike (blunt)	Non-spatial (v3)	β_{13}	*Normal*(0, 1)	-2.57	-1.63	-0.77	0.24
Health	Multiple entanglements (cumulative)	Data-driven (v1)	β_{10}	*Normal*(0, 1)	-0.003	0.012	0.026	0.02
Health	Multiple entanglements (cumulative)	Model-based (v2)	β_{10}	*Normal*(0, 1)	-0.003	0.011	0.026	0.02
Health	Multiple entanglements (cumulative)	Non-spatial (v3)	β_{10}	*Normal*(0, 1)	0.002	0.015	0.028	0.02
Health	Multiple entanglements (occurrence)	Data-driven (v1)	β_{10}	*Normal*(0, 1)	-0.04	0.00	0.05	0.06
Health	Multiple entanglements (occurrence)	Model-based (v2)	β_{10}	*Normal*(0, 1)	-0.05	0.01	0.06	0.06
Health	Multiple entanglements (occurrence)	Non-spatial (v3)	β_{10}	*Normal*(0, 1)	-0.03	0.01	0.06	0.06
Health	Multiple entanglements (occurrence previous 2 y)	Data-driven (v1)	β_{10}	*Normal*(0, 1)	-0.07	-0.02	0.03	0.07
Health	Multiple entanglements (occurrence previous 2 y)	Model-based (v2)	β_{10}	*Normal*(0, 1)	-0.09	-0.03	0.02	0.07

Health	Multiple entanglements (occurrence previous 2 y)	Non-spatial (v3)	β_{10}	*Normal*(0, 1)	-0.09	-0.04	0.00	0.05
Length	Prolonged health (first 15 y)	Non-spatial (v3)	υ_2	*Normal*(0, 1)	-0.37	-0.10	0.18	0.26
Length	Prolonged health (first 10 y)	Non-spatial (v3)	υ_2	*Normal*(0, 1)	-0.30	-0.04	0.22	0.25
Length	Prolonged health (first 5 y)	Non-spatial (v3)	υ_2	*Normal*(0, 1)	-0.32	-0.06	0.21	0.26
Length	Prolonged health (first 2 y)	Non-spatial (v3)	υ_2	*Normal*(0, 1)	-0.37	-0.11	0.16	0.25
Length	Prolonged health (first 1 y)	Non-spatial (v3)	υ_2	*Normal*(0, 1)	-0.40	-0.14	0.13	0.25
Length	Mother's health (start of lactation)	Non-spatial (v3)	υ_2	*Normal*(0, 1)	-0.42	-0.13	0.14	0.27
Length	Mother's health (lactation year)	Non-spatial (v3)	υ_2	*Normal*(0, 1)	-0.40	-0.13	0.13	0.25
Length	Prey × entanglement (mean prey, first 15 y)	Data-driven (v1)	υ_2	*Normal*(0, 1)	-1.78	0.13	2.04	0.94
Length	Prey × entanglement (mean entanglement, first 15 y)	Data-driven (v1)	υ_3	*Normal*(0, 1)	-2.04	-0.26	1.44	0.88
Length	Prey × entanglement (interaction, first 15 y)	Data-driven (v1)	υ_4	*Normal*(0, 1)	-1.94	0.02	1.97	0.98
Length	Prey × entanglement (mean prey, first 15 y)	Model-based (v2)	υ_2	*Normal*(0, 1)	-1.62	0.23	2.12	0.91
Length	Prey × entanglement (mean entanglement, first 15 y)	Model-based (v2)	υ_3	*Normal*(0, 1)	-2.08	-0.26	1.45	0.88
Length	Prey × entanglement (interaction, first 15 y)	Model-based (v2)	υ_4	*Normal*(0, 1)	-1.94	0.04	1.89	0.97
Length	Prey × entanglement (mean prey, first 15 y)	Non-spatial (v3)	υ_2	*Normal*(0, 1)	0.92	1.77	2.57	0.2

Length	Prey × entanglement (mean entanglement, first 15 y)	Non-spatial (v3)	υ_3	*Normal*(0, 1)	-1.24	0.38	2.02	0.84
Length	Prey × entanglement (interaction, first 15 y)	Non-spatial (v3)	υ_4	*Normal*(0, 1)	-1.85	0.11	2.12	0.95
Length	Prey × entanglement (mean prey, first 10 y)	Data-driven (v1)	υ_2	*Normal*(0, 1)	-1.67	0.17	2.04	0.93
Length	Prey × entanglement (mean entanglement, first 10 y)	Data-driven (v1)	υ_3	*Normal*(0, 1)	-1.83	-0.23	1.33	0.87
Length	Prey × entanglement (interaction, first 10 y)	Data-driven (v1)	υ_4	*Normal*(0, 1)	-2.03	-0.01	1.93	0.98
Length	Prey × entanglement (mean prey, first 10 y)	Model-based (v2)	υ_2	*Normal*(0, 1)	-1.70	0.16	1.96	0.93
Length	Prey × entanglement (mean entanglement, first 10 y)	Model-based (v2)	υ_3	*Normal*(0, 1)	-1.86	-0.20	1.40	0.88
Length	Prey × entanglement (interaction, first 10 y)	Model-based (v2)	υ_4	*Normal*(0, 1)	-1.93	0.02	1.93	0.97
Length	Prey × entanglement (mean prey, first 10 y)	Non-spatial (v3)	υ_2	*Normal*(0, 1)	-0.04	0.59	1.19	0.42
Length	Prey × entanglement (mean entanglement, first 10 y)	Non-spatial (v3)	υ_3	*Normal*(0, 1)	-1.05	0.50	2.12	0.77
Length	Prey × entanglement (interaction, first 10 y)	Non-spatial (v3)	υ_4	*Normal*(0, 1)	-1.92	0.00	1.90	0.98
Length	Prey × entanglement (mean prey, first 5 y)	Data-driven (v1)	υ_2	*Normal*(0, 1)	-1.79	0.13	1.99	0.94
Length	Prey × entanglement (mean entanglement, first 5 y)	Data-driven (v1)	υ_3	*Normal*(0, 1)	-2.60	-1.13	0.35	0.52

Length	Prey × entanglement (interaction, first 5 y)	Data-driven (v1)	υ_4	*Normal*(0, 1)	-1.93	-0.04	1.91	0.97
Length	Prey × entanglement (mean prey, first 5 y)	Model-based (v2)	υ_2	*Normal*(0, 1)	-1.79	0.05	1.83	0.96
Length	Prey × entanglement (mean entanglement, first 5 y)	Model-based (v2)	υ_3	*Normal*(0, 1)	-2.47	-1.08	0.42	0.54
Length	Prey × entanglement (interaction, first 5 y)	Model-based (v2)	υ_4	*Normal*(0, 1)	-2.01	-0.04	1.90	0.97
Length	Prey × entanglement (mean prey, first 5 y)	Non-spatial (v3)	υ_2	*Normal*(0, 1)	-0.25	0.17	0.61	0.37
Length	Prey × entanglement (mean entanglement, first 5 y)	Non-spatial (v3)	υ_3	*Normal*(0, 1)	-1.60	-0.20	1.18	0.81
Length	Prey × entanglement (interaction, first 5 y)	Non-spatial (v3)	υ_4	*Normal*(0, 1)	-1.77	0.08	1.93	0.98
Length	Prey × entanglement (mean prey, first 2 y)	Data-driven (v1)	υ_2	*Normal*(0, 1)	-1.82	0.01	1.89	0.96
Length	Prey × entanglement (mean entanglement, first 2 y)	Data-driven (v1)	υ_3	*Normal*(0, 1)	-1.93	-0.80	0.32	0.57
Length	Prey × entanglement (interaction, first 2 y)	Data-driven (v1)	υ_4	*Normal*(0, 1)	-1.99	-0.03	1.93	0.98
Length	Prey × entanglement (mean prey, first 2 y)	Model-based (v2)	υ_2	*Normal*(0, 1)	-1.90	-0.07	1.72	0.95
Length	Prey × entanglement (mean entanglement, first 2 y)	Model-based (v2)	υ_3	*Normal*(0, 1)	-1.94	-0.78	0.39	0.57
Length	Prey × entanglement (interaction, first 2 y)	Model-based (v2)	υ_4	*Normal*(0, 1)	-2.02	-0.02	1.91	0.98
Length	Prey × entanglement (mean prey, first 2 y)	Non-spatial (v3)	υ_2	*Normal*(0, 1)	-0.31	0.10	0.50	0.36

Length	Prey × entanglement (mean entanglement, first 2 y)	Non-spatial (v3)	υ_3	*Normal*(0, 1)	-0.66	0.38	1.40	0.68
Length	Prey × entanglement (interaction, first 2 y)	Non-spatial (v3)	υ_4	*Normal*(0, 1)	-1.89	-0.26	1.38	0.86
Length	Prey × entanglement (mean prey, first 1 y)	Data-driven (v1)	υ_2	*Normal*(0, 1)	-1.47	0.25	1.98	0.89
Length	Prey × entanglement (mean entanglement, first 1 y)	Data-driven (v1)	υ_3	*Normal*(0, 1)	-1.85	-0.93	-0.07	0.46
Length	Prey × entanglement (interaction, first 1 y)	Data-driven (v1)	υ_4	*Normal*(0, 1)	-2.01	-0.05	1.86	0.97
Length	Prey × entanglement (mean prey, first 1 y)	Model-based (v2)	υ_2	*Normal*(0, 1)	-1.65	0.16	1.97	0.91
Length	Prey × entanglement (mean entanglement, first 1 y)	Model-based (v2)	υ_3	*Normal*(0, 1)	-1.88	-0.85	0.07	0.5
Length	Prey × entanglement (interaction, first 1 y)	Model-based (v2)	υ_4	*Normal*(0, 1)	-2.03	0.00	2.03	0.97
Length	Prey × entanglement (mean prey, first 1 y)	Non-spatial (v3)	υ_2	*Normal*(0, 1)	-0.20	0.16	0.50	0.32
Length	Prey × entanglement (mean entanglement, first 1 y)	Non-spatial (v3)	υ_3	*Normal*(0, 1)	-0.83	0.14	1.11	0.66
Length	Prey × entanglement (interaction, first 1 y)	Non-spatial (v3)	υ_4	*Normal*(0, 1)	-1.82	-0.30	1.27	0.82
Length	Mother's entanglement status	Data-driven (v1)	υ_2	*Normal*(0, 1)	-0.53	-0.22	0.09	0.28
Length	Mother's entanglement status	Model-based (v2)	υ_2	*Normal*(0, 1)	-0.54	-0.22	0.09	0.29
Length	Mother's entanglement status	Non-spatial (v3)	υ_2	*Normal*(0, 1)	-0.54	-0.22	0.09	0.3

Exposure	Prey × entanglement risk	Data-driven (v1)	β_{10}	*Normal*(0, 1)	-0.0004	- 0.0002	0.0000	0.01
Exposure	Prey × entanglement risk	Model-based (v2)	β_{10}	*Normal*(0, 1)	0.0023	0.0029	0.0035	0.01
Exposure	Prey × vessel strike risk	Data-driven (v1)	β_{10}	*Normal*(0, 1)	-0.55	-0.10	0.33	0.38
Exposure	Prey × vessel strike risk	Model-based (v2)	β_{10}	*Normal*(0, 1)	-0.75	0.02	0.75	0.57

Section S11: Predicted exposure to stressors

Section S11.1: Data-driven spatial distribution model (v1, from NARW Consortium sightings)

<u>Section S11.1.1: Entanglement probability</u>

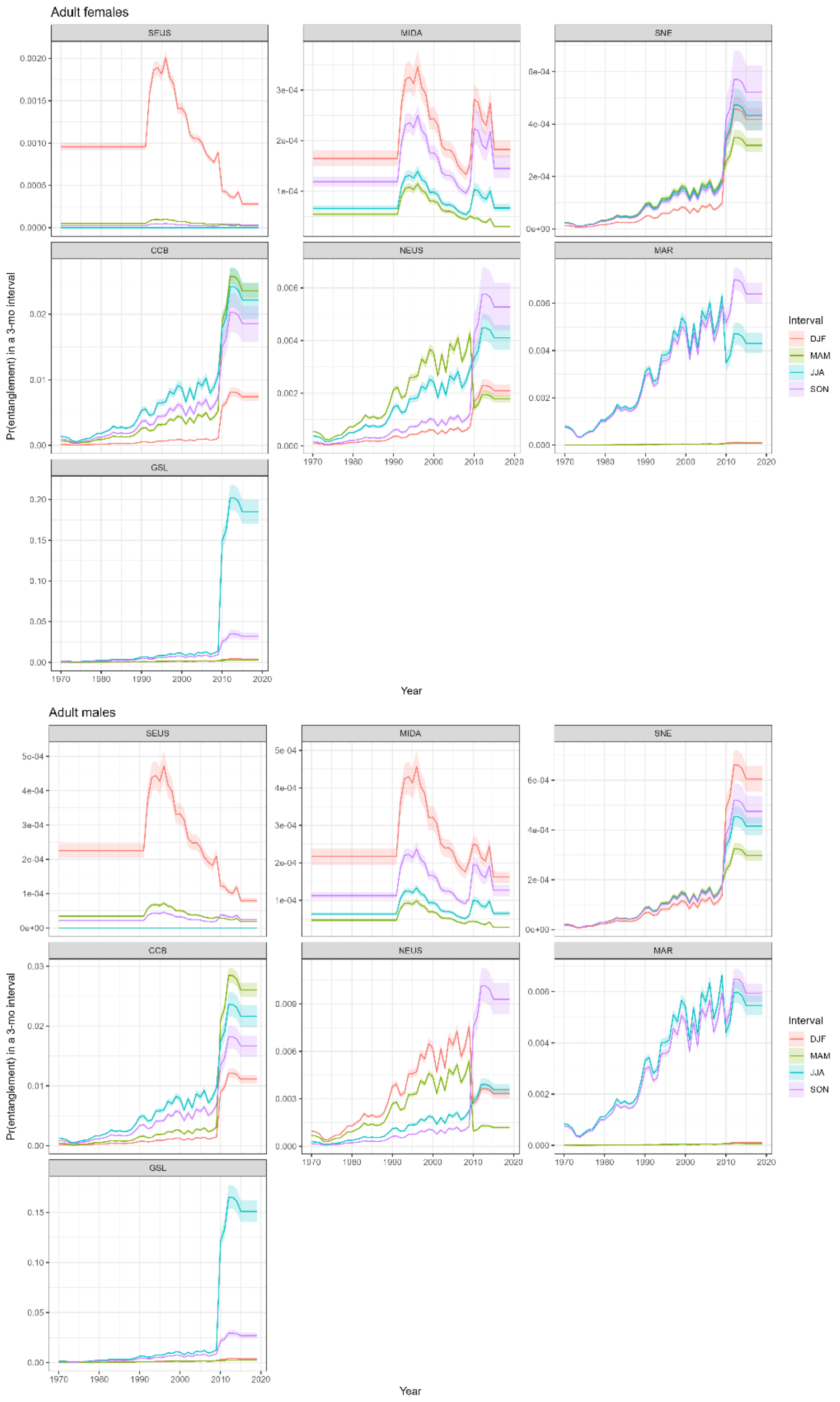

Figure S8. Posterior probability of entanglement, in each three-month interval (indicated by the initials of the corresponding months; DJF, MAM, JJA and SON) and regional polygon

(Southeastern U.S., SEUS; Mid-Atlantic, MIDA; Southern New England, SNE; Cape Cod Bay, CCB; Northeastern U.S., NEUS; Maritimes, MAR; Gulf of St Lawrence, GSL), for adult females (top) and adult males (bottom), from the model where individual spatial distribution is inferred from all NARW Consortium sightings (v1). The lines indicate the posterior medians, while the ribbons are the 95% credible intervals. Note that we use a different scale for the y-axis across panels, for visualisation (due to the higher probability estimated for GSL). Entanglement probabilities were obtained from the product of the entanglement risk (derived from the WHALE DST), the trend in landings over time (from the Atlantic Coastal Cooperative Statistics Program) and the estimated distribution of different demographic classes (π), converted into a probability via the scalar ι_1. The estimated entanglement probability in Cape Cod Bay does not fully reflect the ban on fixed gear use in the Bay between February and April after 2015, as discussed for entanglement risk in Figure S3; this is exacerbated when estimating entanglement probability, because of the high estimated occurrence of NARW in this region in the first months of the year (Fig. S1) and the uniform whale distribution assumed in the Bay (Roberts et al. 2024).

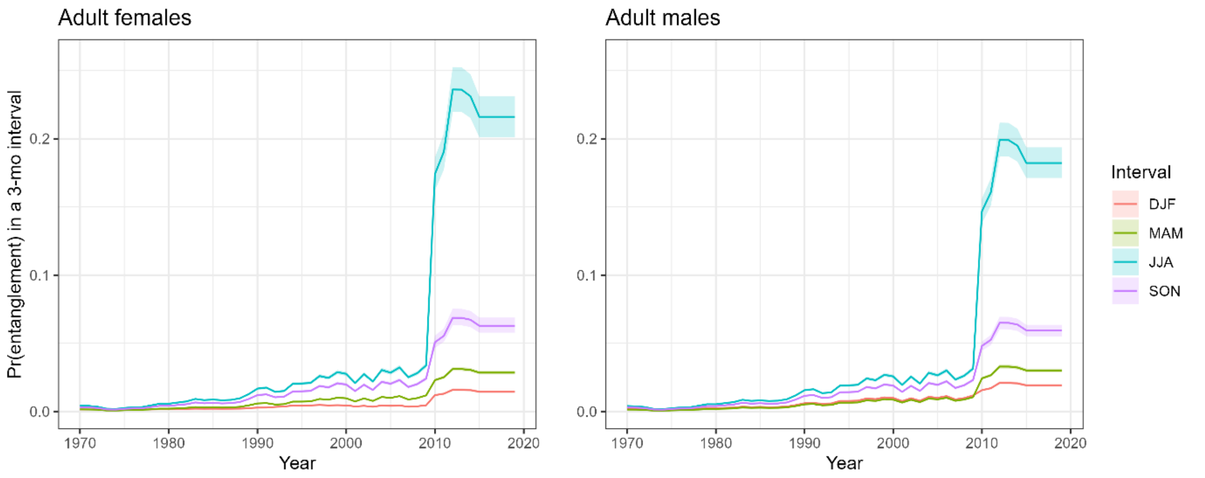

Figure S9. Average posterior probability of entanglement in each three-month interval (indicated by the initials of the corresponding months; DJF, MAM, JJA and SON) summarised across regions, for adult females (top) and adult males (bottom), from the model where individual spatial distribution is inferred from all NARW Consortium sightings (v1). The lines indicate the posterior medians, while the ribbons are the 95% credible intervals.

<u>Section S11.1.2: Vessel strike probability</u>

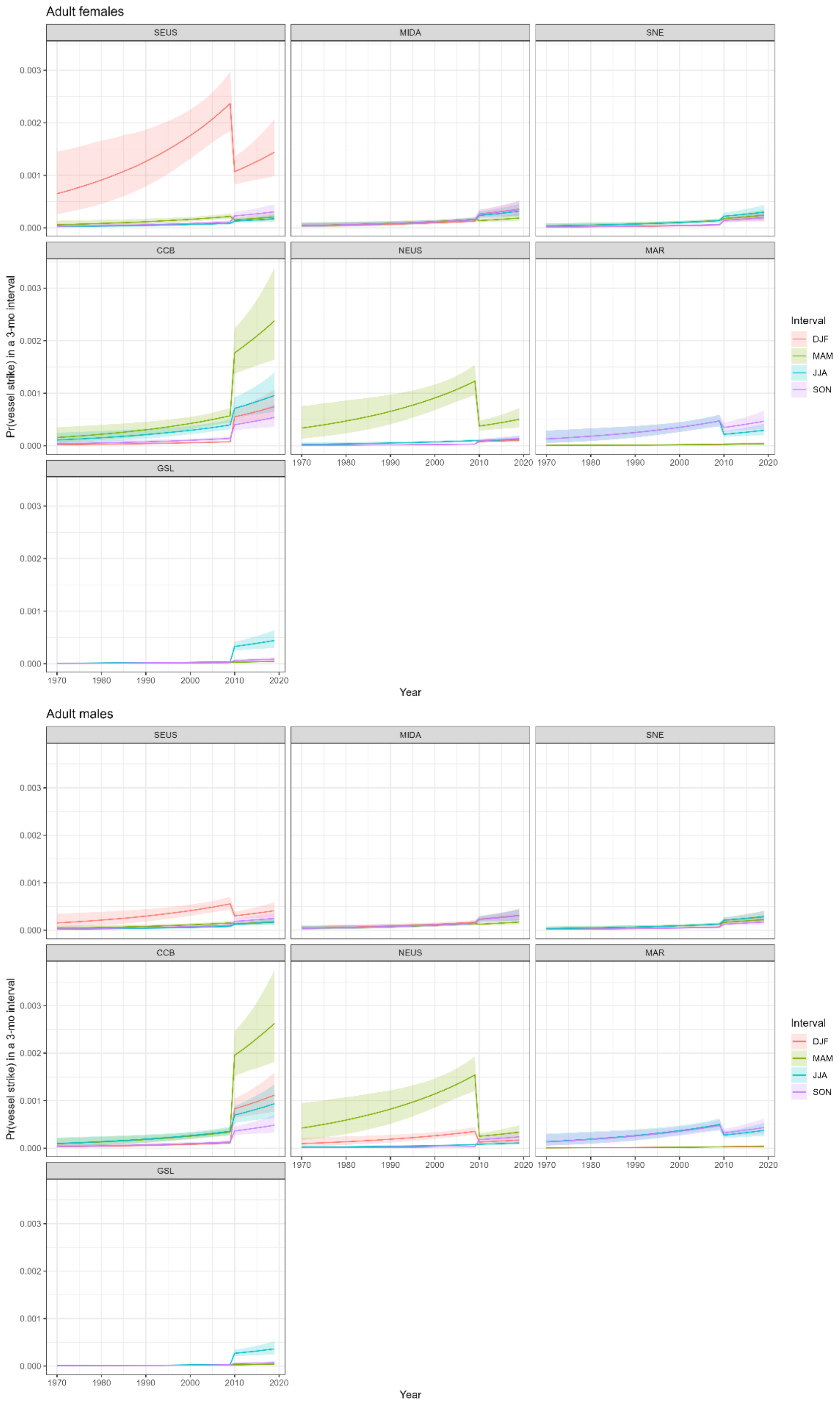

Figure S10. Posterior probability of vessel strike, in each three-month interval (indicated by the initials of the corresponding months; DJF, MAM, JJA and SON) and regional polygon

(Southeastern U.S., SEUS; Mid-Atlantic, MIDA; Southern New England, SNE; Cape Cod Bay, CCB; Northeastern U.S., NEUS; Maritimes, MAR; Gulf of St Lawrence, GSL), for adult females (top) and adult males (bottom), from the model where individual spatial distribution is inferred from all NARW Consortium sightings (v1). The lines indicate the posterior medians, while the ribbons are the 95% credible intervals. Vessel strike probabilities were obtained from the product of the vessel strike risk (derived from AIS data), the estimated trend in traffic over time and the estimated distribution of different demographic classes (π), converted into a probability via the scalar ι_2. The discontinuities in the linear trends are due to the change in NARW distribution from 2010 onwards.

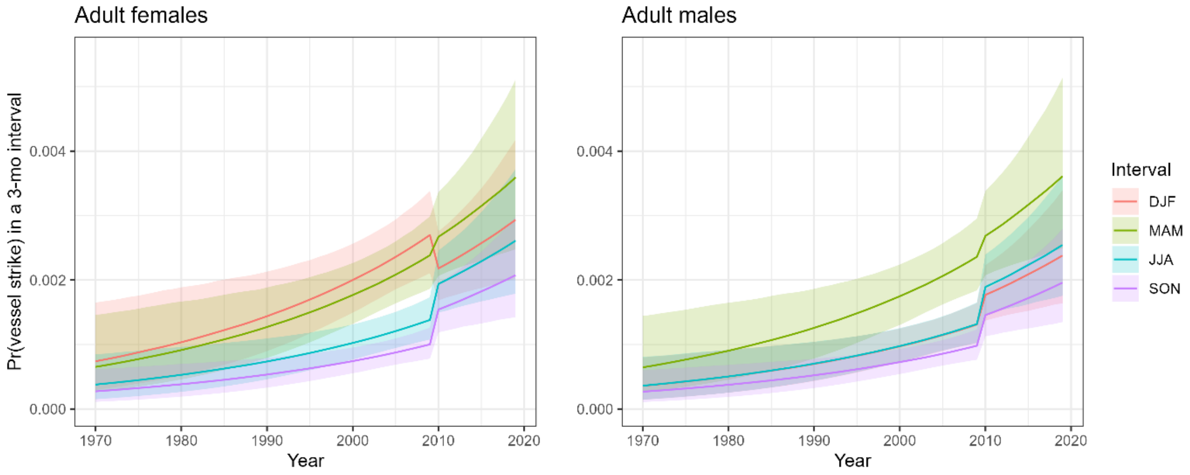

Figure S11. Average posterior probability of vessel strike in each three-month interval (indicated by the initials of the corresponding months; DJF, MAM, JJA and SON) summarised across regions, for adult females (top) and adult males (bottom), from the model where individual spatial distribution is inferred from all NARW Consortium sightings (v1). The lines indicate the posterior medians, while the ribbons are the 95% credible intervals. The discontinuities in the linear trends are due to the change in NARW distribution from 2010 onwards.

Section S11.1.3: Mean annual prey conditions

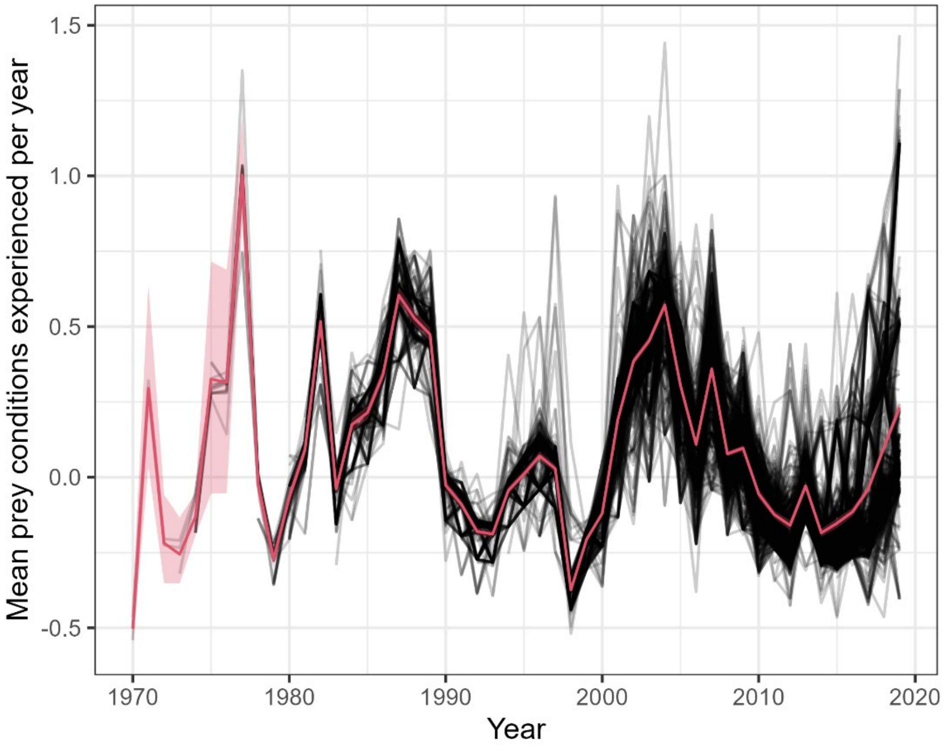

Figure S12. Posterior mean normalised annual prey conditions experienced by NARW individuals, from the model where individual spatial distribution is inferred from all NARW Consortium sightings (v1). Each black line corresponds to an individual, while the red line and ribbon represent the posterior median and 95% credible interval of the mean conditions in each year across individuals (note that the credible interval gets too narrow to be visible after ~1980). These values correspond to the standardised covariate that was used in the linear predictor of individual health.

Section S11.2: Model-based spatial distribution model (v2, from Roberts et al. 2024)

<u>Section S11.2.1: Entanglement probability</u>

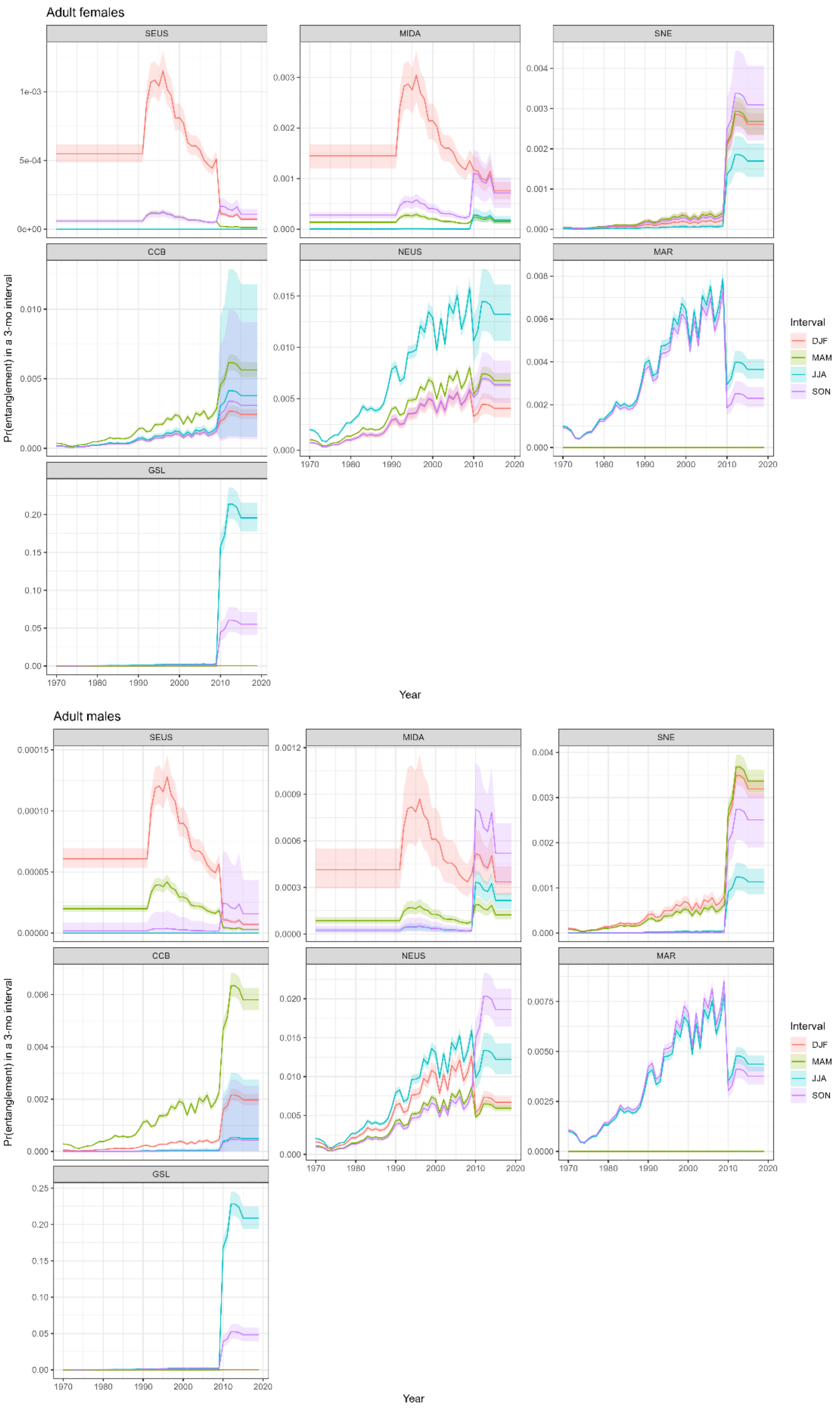

Figure S13. Posterior probability of entanglement, in each three-month interval (indicated by the initials of the corresponding months; DJF, MAM, JJA and SON) and regional polygon

(Southeastern U.S., SEUS; Mid-Atlantic, MIDA; Southern New England, SNE; Cape Cod Bay, CCB; Northeastern U.S., NEUS; Maritimes, MAR; Gulf of St Lawrence, GSL), for adult females (top) and adult males (bottom), from the model where individual spatial distribution is inferred from predictions of NARW density from Roberts et al. (2024) (v2), combined with a model for the proportion of individuals in different demographic classes. The lines indicate the posterior medians, while the ribbons are the 95% credible intervals. Note that we use a different scale for the y-axis across panels, for visualisation (due to the higher probability estimated for GSL). Entanglement probabilities were obtained from the product of the entanglement risk (derived from the WHALE DST), the trend in landings over time (from the Atlantic Coastal Cooperative Statistics Program) and the estimated distribution of different demographic classes (derived from $\tilde{l}$ *and* $\tilde{p}$*), converted into a probability via the scalar* ι_l*.*

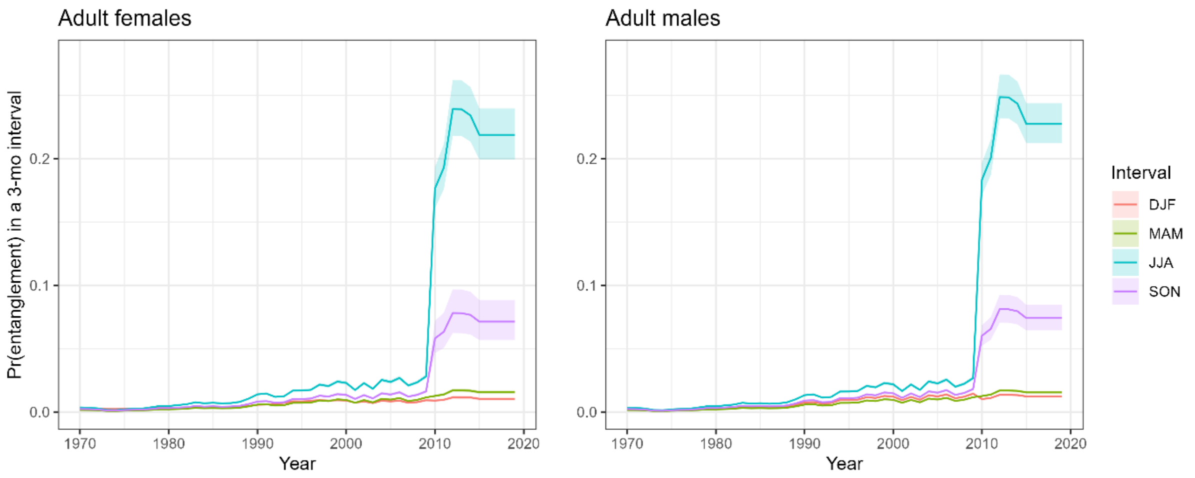

Figure S14. Average posterior probability of entanglement in each three-month interval (indicated by the initials of the corresponding months; DJF, MAM, JJA and SON) summarised across regions, for adult females (top) and adult males (bottom), from the model where individual spatial distribution is inferred from predictions of NARW density from Roberts et al. (2024) (v2), combined with a model for the proportion of individuals in different demographic classes. The lines indicate the posterior medians, while the ribbons are the 95% credible intervals.

Section S11.2.2: Vessel strike probability

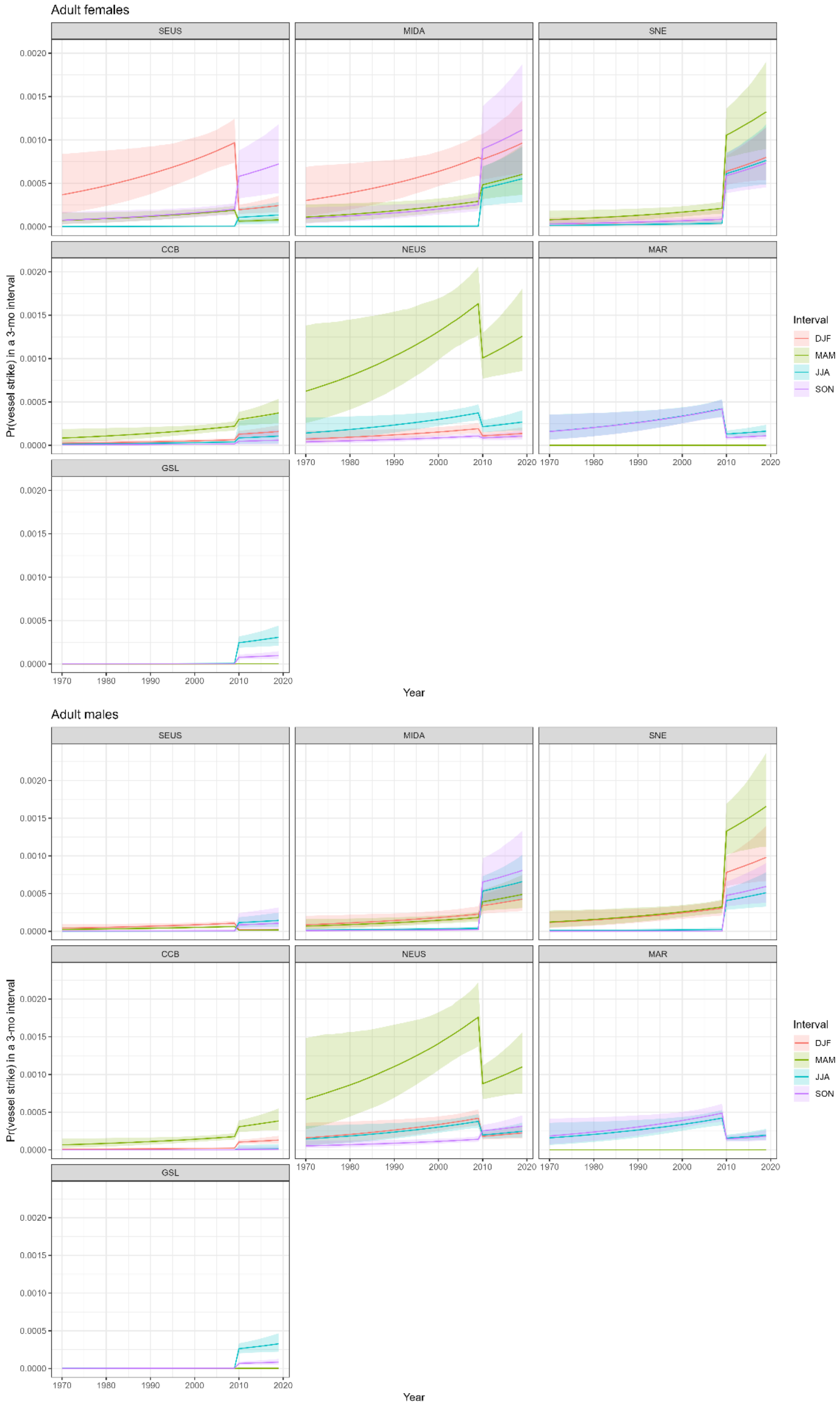

Figure S15. Posterior probability of vessel strike, in each three-month interval (indicated by the initials of the corresponding months; DJF, MAM, JJA and SON) and regional polygon

(Southeastern U.S., SEUS; Mid-Atlantic, MIDA; Southern New England, SNE; Cape Cod Bay, CCB; Northeastern U.S., NEUS; Maritimes, MAR; Gulf of St Lawrence, GSL), for adult females (top) and adult males (bottom), from the model where individual spatial distribution is inferred from predictions of NARW density from Roberts et al. (2024) (v2), combined with a model for the proportion of individuals in different demographic classes. The lines indicate the posterior medians, while the ribbons are the 95% credible intervals. Vessel strike probabilities were obtained from the product of the vessel strike risk (derived from AIS data), the estimated trend in traffic over time and the estimated distribution of different demographic classes (derived from $\tilde{l}$ and $\tilde{p}$), converted into a probability via the scalar ι_2. The discontinuities in the linear trends are due to the change in NARW distribution from 2010 onwards.

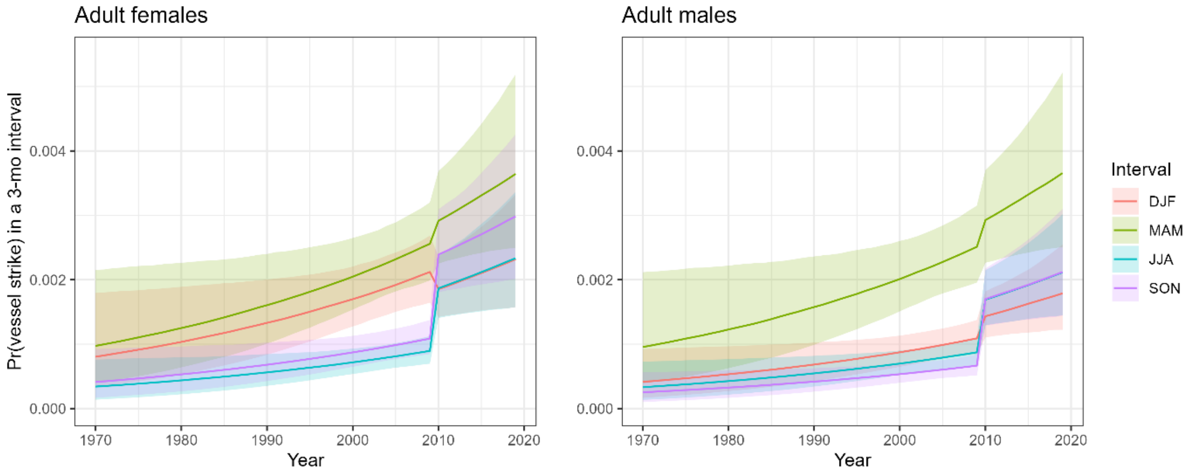

Figure S16. Average posterior probability of vessel strike in each three-month interval (indicated by the initials of the corresponding months; DJF, MAM, JJA and SON) summarised across regions, for adult females (top) and adult males (bottom), from the model where individual spatial distribution is inferred from predictions of NARW density from Roberts et al. (2024) (v2), combined with a model for the proportion of individuals in different demographic classes. The lines indicate the posterior medians, while the ribbons are the 95% credible intervals. The discontinuities in the linear trends are due to the change in NARW distribution from 2010 onwards.

Section S11.2.3: Mean annual prey conditions

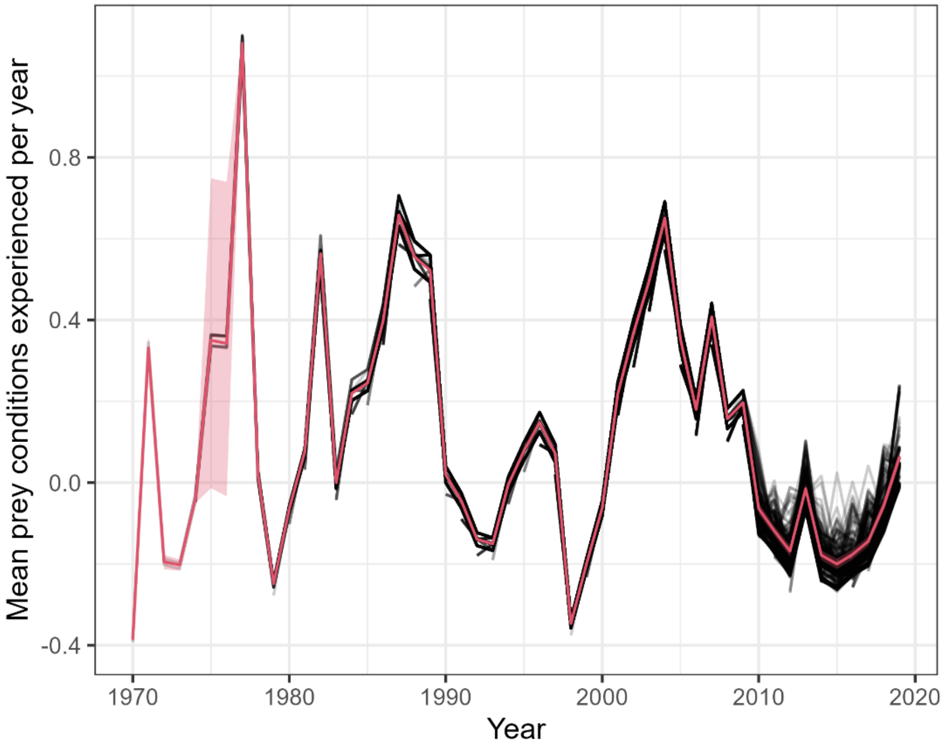

Figure S17. Posterior mean normalised annual prey conditions experienced by NARW individuals, from the model where individual spatial distribution is inferred from predictions of NARW density from Roberts et al. (2024) (v2), combined with a model for the proportion of individuals in different demographic classes. Each black line corresponds to an individual, while the red line and ribbon represent the posterior median and 95% credible intervals of the mean conditions in each year across individuals (note that the credible interval gets too narrow to be visible after ~1980). These values correspond to the standardised covariate that was used in the linear predictor of individual health.

Section S12: Estimating the trend in entanglement risk prior to 2015

The predicted entanglement risk derived from the outputs of Woods Hole Analysis of Line Entanglement Decision Support Tool (WHALE DST) is broadly representative of the risk after 2015 (Miller et al. 2025). In the model presented in the main text, we predicted entanglement risk in prior years using the trend in landings of fisheries that constitute a risk for NARW (given the region) over time. Here, we present a version of the model where the temporal trend of entanglement risk is estimated within the model. Specifically, we model the mean trend in entanglement risk, $\tilde{m}$, and a region-level random effect around it (with standard deviation σ_e), resulting in the trend in each region l, $\tilde{r}_l$. Similar to the model for vessel strike probability, entanglement probability in region l in time step t (in year y_t < 2015) was therefore modelled as:

$$logit(p^n_{l,t}) = logit(\iota_1 \, G_{l,t}) + \tilde{r}_l \, (2015 - y_t). \qquad S38$$

We limited this exploration to the formulation of the model where the spatial distribution of individuals is estimated using the sightings in the NARW Consortium database (model v1).

The results indicated that, prior to 2015, entanglement probability decreased in the Southeastern U.S., Northeastern U.S. and Canadian Maritimes polygons. In contrast, the trend was estimated to be highly uncertain in the mid-Atlantic, Southern New England and, largely, Cape Cod Bay. Finally, entanglement probability was estimated to have increased dramatically in the Gulf of St Lawrence (Fig. S18). Therefore, the estimated regional trends in entanglement probability differ from the trends in the model version presented in the main text in most northern regions except the Gulf of St Lawrence. The estimated trends represent, in theory, entanglement probability not weighted by whale attendance to a region. However, it should be noted that the model estimates risk based on realised entanglement events and predicted whale use of the different regions, and thus has limited information to estimate the risk in regions and seasons where whales did not occur; in fact, preliminary exploration of a model where the trend was allowed to vary by season as well as by region suggested that this further complexity may be warranted, but it also showed issues with convergence due to data limitations. Therefore, we believe that the mechanistically informed version of the model presented in the main text is more appropriate than the one presented here.

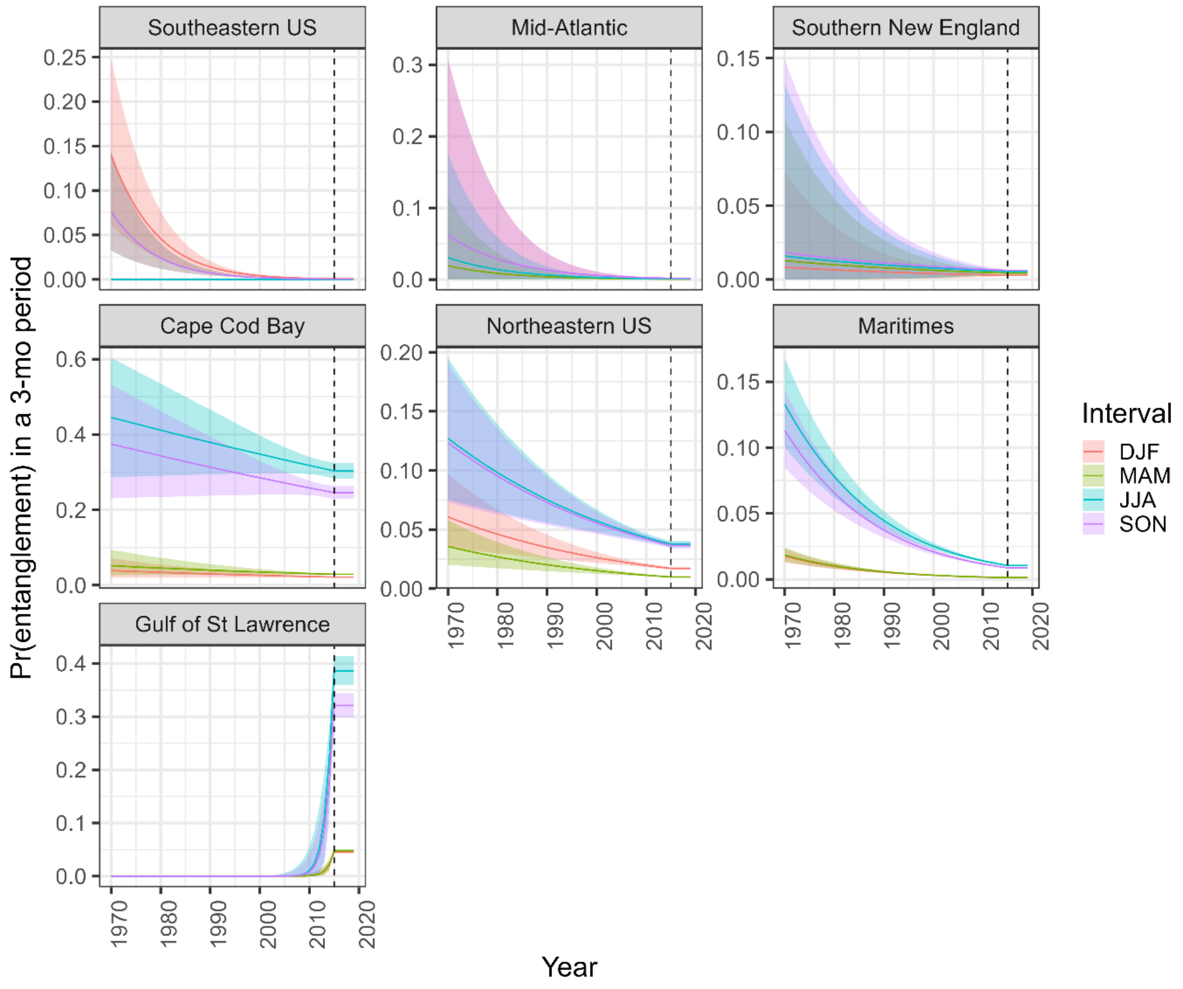

Figure S18. Posterior probability of entanglement, in each three-month interval (indicated by the initials of the corresponding months; DJF, MAM, JJA and SON) and regional polygon (Southeastern U.S., Mid-Atlantic, Southern New England, Cape Cod Bay, Northeastern U.S., Maritimes, Gulf of St Lawrence), from the model where the trend is estimated from the data rather than based on fisheries landings. The lines indicate the posterior medians, while the ribbons are the 95% credible intervals. Note that we use a different scale for the y-axis across panels, for visualisation.

Section S13: Example of a combined effect of two stressors

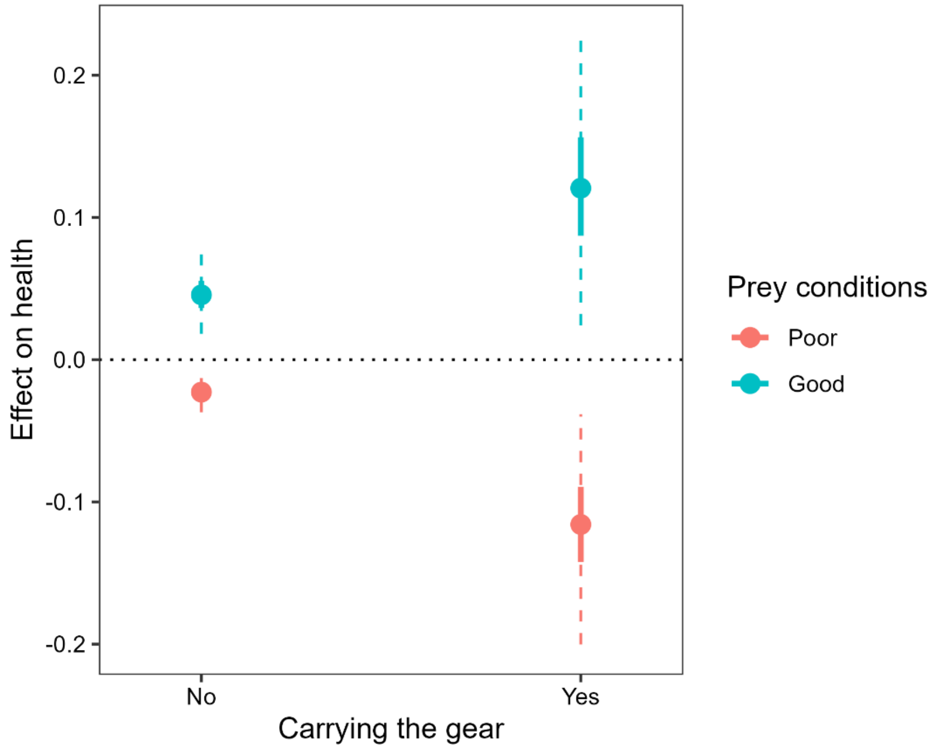

Figure S19. Example of a combined effect of prey conditions and entanglements in the data-driven formulation of the spatial model (v1). The dots indicate the median effects on health, the solid lines are the interquartile ranges, while the dashed lines are the 95% credible interval (CI). The prolonged effect of carrying the gear on health is negative when prey conditions are poor. The 95% CI of this effect overlaps with the 95% CI of the main effect of prey when prey conditions are good, although the interquartile ranges do not.

Section S14: Assessing future stressor scenarios using model results in a population viability analysis

Using the results of our model in the existing NARW population viability analysis (PVA) (Runge et al. 2023) required a series of modifications of both the Bayesian state-space model described here and the PVA. The PVA has an annual time step (July to June) and formulates survival probabilities in a given year in terms of the mortality hazard rates from severe entanglements and all vessel strikes (Runge et al. 2023), which are estimated from the data in separate submodels and then used in stochastic forward projections of the population's trajectory. In order to facilitate the integration of our results in the simulation tool, we reformulated one version of our model (with data-driven spatial structure, i.e., model v1, and including the combined effect of prey conditions and the prolonged effect of entanglements) as follows.

Section S14.1: Change to survival-health link function

We first changed the form of the function that links the health of individual i in time step t, $h_{i,t}$, to survival probability $\vartheta_{i,t}$ to match that of the PVA. Our models had used a complementary log-log link function (described in section S1) while the PVA used a log-log link (Ergon et al. 2018). The new link function has the form

$$\vartheta_{i,t} = \exp\left(-\exp\left(-h_{i,t}\right)\right). \qquad S39$$

Note that we changed the sign of health to be able to retain the intuitive interpretation of health as in the complementary log-log formulation (i.e., a higher value of health corresponds to greater survival probability). Using this formulation, exp($-h_{i,t}$) corresponds to the time-averaged mortality hazard rate over a three-month interval; it can then be shown that the exponential of a stressor coefficient α (for intrinsic stressors) or β (for extrinsic stressors) corresponds to the hazard ratio associated with that stressor (Ergon et al. 2018).

Changing the link function affects the trajectory of the latent health state over time; hence we had to update the fixed value of health process variability, σ (Pirotta et al. 2023). Specifically, we re-ran the non-spatial formulation of the model (v3), allowing for the estimation of σ. The new posterior mean for this parameter was 0.16 (as compared to 0.1 when using the complementary log-log link function). Even after fixing σ, this model formulation showed poorer mixing of the parameters of the health observation models (i.e., linking the health state to the four visual health assessment variables; see section S1), which required us to run the model for 100,000 iterations over 10 chains (thinning to 1 in 100 iterations) to obtain a satisfactory posterior effective sample size (Lunn et al. 2013).

Section S14.2: Calculating annual survival

The PVA assumes that the population census occurs in July of each year, which determines the age at which calves first appear in the model (approximately 6 months). Here, we assumed that the simulation started in June to align the PVA year with the three-month intervals in our

Bayesian model; however, because the exact birth date of calves is unknown, we still assumed that calves were 6 months old at the start of the simulation. We took the posterior health of the individuals alive in the Mar-May interval of 2019 to represent the distribution of baseline health and survival probability at the start of the simulation. We also used this distribution to inform the distribution of health and survival in the absence of stressors and when assigning a health value to newborn animals. To do so, if animals were exposed to stressors in that time step, we removed the corresponding effects from their estimated health status. The survival probability of an individual in a given year of the PVA simulation could then be obtained from the hazard ratios of the stressors to which the individual was exposed, multiplied by the baseline mortality hazard rate in the absence of stressors. It should be noted that, due to the finer time scale of our model (3 months vs. 1 year), the combined survival probability for year y is the product of the survival probability in the four three-month steps ($\vartheta^q_{y,1:4}$), each including the effects of any stressors in that time step and the value of health from the previous time step (to account for the autocorrelation in the process model for health within that year, see section S1). For example, simplifying the notation, the survival probability of an individual in year y, ϑ_y, assuming that it suffered a vessel strike in the first time step and that the effect of mean prey conditions also applied in the first time step (i.e., in subsequent time steps, health was just equal to health in the first step), is:

$$\begin{aligned}
\vartheta_y &= \vartheta^q_{y,1}\,\vartheta^q_{y,2}\,\vartheta^q_{y,3}\,\vartheta^q_{y,4} \\
&= \exp\big(-\exp(-h_{y,1})\big)\exp\big(-\exp(-h_{y,2})\big)\exp\big(-\exp(-h_{y,3})\big)\exp\big(-\exp(-h_{y,4})\big) \\
&= \exp\big(-\exp(-h_{y,1})\big)\exp\big(-\exp(-h_{y,1})\big)\exp\big(-\exp(-h_{y,1})\big)\exp\big(-\exp(-h_{y,1})\big) \\
&= \exp\big(-\exp(-h_{y,1})\big)^{4} \\
&= \exp\big(-4\exp(-h_{y,1})\big) \\
&= \exp\big(-4\exp(-(h_0+\beta_{strike}+\beta_{prey}\,prey_y))\big) \\
&= \exp\big(-4\exp(-h_0)\exp(-\beta_{strike})\exp(-\beta_{prey}\,prey_y)\big) \qquad S40
\end{aligned}$$

where $h_{y,1:4}$ are the values of health in each three-month time step of that year (in this case, all equal to $h_{y,1}$ because the stressors only applied in the first interval), h_0 is the baseline health in the absence of stressors, β_{strike} is the coefficient for the effect of a strike, and β_{prey} is the coefficient for the effect of mean prey conditions, multiplied by the value of the covariate in that year ($prey_y$). This calculation was modified to reflect different combinations of stressors simulated to occur for an individual in a year.

Section S14.3: Accounting for temporal autocorrelation in survival probability

The above implementation does not consider any autocorrelation in survival probability from year to year; that is, an individual's survival in year y is affected by the set of stressors experienced that year, but the calculation of its survival in year y+1 is then based on h_0, thus

ignoring any protracted effect of those stressors. Exploration of the autocorrelation in estimated health (and thus survival probability) in the Bayesian state-space model suggested that autocorrelation was strong and extended over multiple years (e.g., Fig. S20). As a result, the effects of stressors on individual survival will be underestimated when incorporating the results of our Bayesian model (where health is autocorrelated and the impact of a trauma on survival persists over multiple years) into the PVA tool (which ignores year-to-year autocorrelation). Ideally, we would formulate health in the PVA to follow the same random walk process as in the Bayesian state-space model; however, preliminary exploration showed that the random walk, being unconstrained, led to the simulated health trajectory diverging from realistic values.

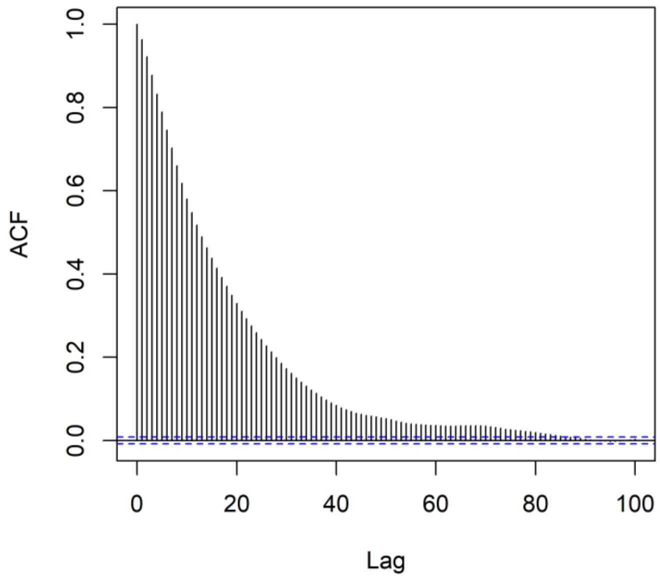

Figure S20. Autocorrelation function (ACF) plot for the posterior health of NARW individuals for increasing time lag (in three-month intervals), from the model where individual spatial distribution is inferred from all NARW Consortium sightings (v1).

To temporarily address this critical difference between the two approaches for this demonstrative exercise, we also computed the value of survival probability, ϑ'_y, that resulted from accounting for the previous survival probability of an individual, i.e., substituting h_0 with the value of health in a three-month time step obtained from the individual's survival in the previous year, $h'_0 = -\log\left(-\log\left(\sqrt[4]{\vartheta^w_{y-1}}\right)\right)$. We then took survival probability in a given year to be the weighted average between the survival probability calculated using h_0 and the survival probability that accounted for autocorrelation, i.e.

$$\vartheta^w_y = (1 - w)\,\vartheta_y + w\,\vartheta'_y. \qquad S41$$

The weighting, w, between the two values (representing the strength of the autocorrelation in survival over time) was calibrated using NARW population trend under the *status quo* stressor

scenario in Runge et al. (2023) (i.e., no reduction of entanglement and vessel strikes, and prey conditions to remain as in the decade 2010-2019). Specifically, we re-ran the PVA using one of 14 weight values equally spaced between w = 0.5 and 0.89 for the survival probability accounting for autocorrelation (while the survival probability calculated using h_0 was weighted by $1 - w$). We then fitted a Generalised Additive Model to the difference between the median population size at the end of the simulation and the median final population size for the *status quo* scenario in Runge et al. (2023) (N = 90), and used the fitted model to infer the weight value that minimised the absolute difference between the two (w = 0.685; Fig. S21). Because our implementation of the PVA also modelled individual's length and its effect on calving probability (see below), this calibration was carried out under a scenario where the mean asymptotic length was equal to the mean asymptote in 2019.

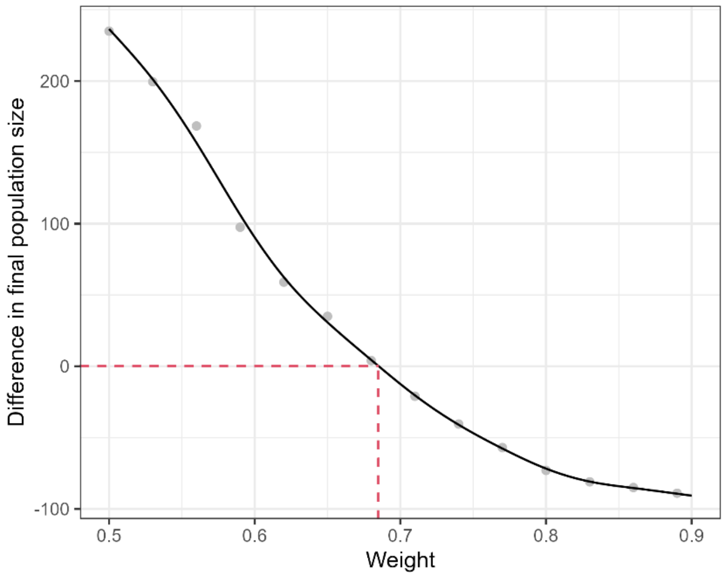

Figure S21. Results of the calibration of the weight for survival probability accounting for autocorrelation. The grey dots represent the median difference between the final population size obtained from the simulation given a weight value on the x-axis, and the final population size in the status quo scenarios in Runge et al. (2023) (N = 90). The black line is the mean relationship estimated using a Generalised Additive Model. The red segments highlight the weight value that minimised the absolute difference between the two final population sizes (w = 0.685).

Section S14.4: Exposure to stressors

We retained the approach described in Runge et al. (2023) to model the probability of experiencing entanglements and vessel strikes in each simulation year of the PVA. However, given that here we modelled survival probability at a finer temporal scale, these events had to be allocated to a specific three-month step, and, for simplicity, we assumed that they occurred in the first time step of a simulated year.

Because the authors only modelled the occurrence of severe events, we only used the hazard ratio of the immediate effect of severe entanglements from the Bayesian model. Entanglement events were assumed to last for the median duration of severe events estimated in the model, i.e., two three-month time steps; the hazard rate corresponding to the immediate effect of an event was applied in the first time step of a PVA simulation year, while the hazard rate corresponding to the prolonged effect of carrying the gear was applied in the second. For vessel strikes, we used the mean hazard ratios of vessel strikes resulting in a deep or blunt injury weighted by their proportional occurrence (derived from $p^{w}_{1:4}$), and assumed that the simulated events occurred in the first time step of a PVA simulation year. For prey conditions, the equation for health in the Bayesian model was modified to estimate the effect of the deviation of the mean prey conditions experienced by an individual in a given year from the best possible prey conditions observed across the entire study period. The latter was calculated assuming that an individual spent the entirety of each three-month interval in each year in the region with the best prey index. This reformulation ensured that the corresponding coefficient was estimated to be negative, representing the additional hazard of mortality resulting from prey conditions below the maximum.

Section S14.5: Model for fecundity

The PVA code was also modified to model females' calving probability (when aged 9 y or older) as a function of their health at the end of a simulated year via a sigmoid function, as in the Bayesian state-space model (see section S1). The individual random effect on the asymptote of the sigmoid function in the PVA was stochastically drawn and assigned to each female at birth (given the estimated standard deviation χ), together with the value of the binary variable that determined whether a female could ever reproduce or would remain nulliparous (drawn with probability v). Moreover, we included a model in the PVA for individual growth in length, using the parameters estimated in the Bayesian model, and its effect on calving probability. The asymptotic length an individual could grow to in the PVA was assigned at birth and varied under different simulated scenarios (see below); an individual was then simulated to grow each year according to its age.

Section S14.6: PVA scenarios

Using the modified PVA code, the population's trajectory over the next 100 y was simulated under a set of scenarios corresponding to all combinations of different stressor changes as follows:

- Reductions in severe entanglement probability were simulated, corresponding to 0, 50, and 100% of current levels.
- Prey conditions were simulated to remain low, as in the decade 2010-2019, or to return to the historical variation observed in 1990-2009 (note that we do not simulate changes in the spatial distribution of the prey).
- NARW asymptotic length was simulated to stabilise at the current estimated mean (~11.8 m), continue to decline at the current rate (down to a minimum of 10 m), or

revert its trend (with the same slope of the current decline, but opposite sign) up to the historical maximum (~14.0 m).

As in the original PVA by Runge et al. (2023), we propagated the uncertainty in model estimates by repeating the population projections using 1,000 samples from the input distributions for the model.

Section S14.7: Results and discussion

The results confirm the conclusion of Runge et al. (2023) that substantial reductions in severe entanglement risk are required to ensure the viability of the species (Fig. 6 in main text and Fig. S22). Beyond this result, our simulations also show that, if the current downward trend in asymptotic size were to continue in the future, the resulting calving probability may be insufficient to support a positive growth rate, on average. In contrast, an inversion of that trend would substantially improve the chances of the population to recover and survive in the future. Prey conditions do not appear to have any visible influence on the population's trajectory, despite their effect on health and the interaction with the prolonged effect of entanglements. However, it should be noted that prey availability is probably involved in the process driving the observed decline in NARW asymptotic size, even though we did not identify this role in our analyses for the reasons discussed in the main text. Therefore, the importance of prey might be captured by the influence of the trend in body length on population growth rate.

We note that the results of the simulations integrating our findings into the existing PVA framework were highly sensitive to the initial population size and structure, the baseline distribution of health (i.e., survival) values in the absence of stressors, and the strength of the autocorrelation from year to year. These sensitivities currently limit the interpretation of the simulated population's absolute size, thus preventing any use of these specific results for management purposes. However, we expect the conclusions around the relative comparisons among alternative stressor reductions to be more robust to these limitations. More generally, our primary goal was to lay out a roadmap for the incorporation of the results of the Bayesian state-space model in the existing PVA tool, and to demonstrate the outstanding obstacles. A more extensive investigation of the differences between the modelling approaches and, ideally, a coherent amalgamation of the two is needed to inform management efforts.

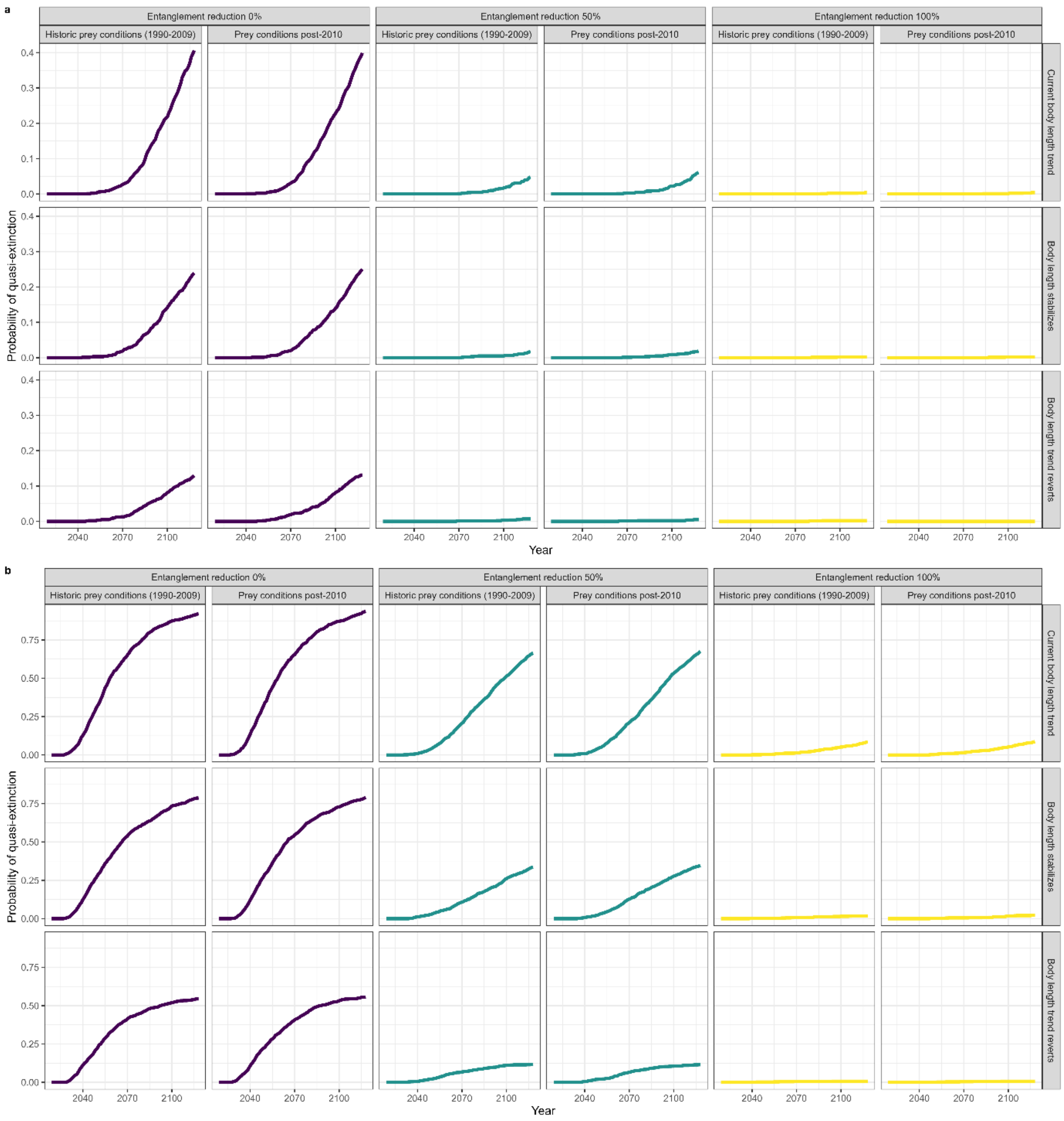

Figure S22. Probabilities of quasi-extinction for proven NARW females (individuals known to have produced calves) at thresholds of N = 10 (a) and N = 50 individuals (b), under various scenarios of severe entanglement risk reduction (0, 50 or 100%), prey conditions (following historical patterns from 1990-2009, or reduced as in the post-2010 period), and asymptotic body length (trend continuing along the current downward trajectory, stabilising, or reverting towards the historical maximum size). The probability of quasi-extinction is defined as the probability that the population has dropped below a given threshold by or at a given simulated year, which is calculated as the fraction of bootstrap replicates for which the quasi-extinction criterion is met by or at a given simulated year (Runge et al. 2023).

Section S15: References

Baumgartner, M. F., T. V. N. Cole, R. G. Campbell, G. J. Teegarden, and E. G. Durbin. 2003. Associations between North Atlantic right whales and their prey, *Calanus finmarchicus*, over diel and tidal time scales. Marine Ecology Progress Series 264:155–166.

Baumgartner, M. F., and B. R. Mate. 2003. Summertime foraging ecology of North Atlantic right whales. Marine Ecology Progress Series 264:123–135.

Baumgartner, M. F., and B. R. Mate. 2005. Summer and fall habitat of North Atlantic right whales (*Eubalaena glacialis*) inferred from satellite telemetry. Canadian Journal of Fisheries and Aquatic Sciences 62:527–543.

Baumgartner, M. F., F. W. Wenzel, N. S. J. Lysiak, and M. R. Patrician. 2017. North Atlantic right whale foraging ecology and its role in human-caused mortality. Marine Ecology Progress Series 581:165–181.

Blais, M., P. S. Galbraith, S. Plourde, M. Lizotte, S. A. Clay, and M. Starr. 2024. Chemical and biological oceanographic conditions in the estuary and Gulf of St. Lawrence during 2023. Can. Tech. Rep. Hydrogr. Ocean Sci. 385:v + 84 pp.

Carlowicz Lee, R. M., T. D. Keiling, and J. D. Warren. 2024. Seasonal abundance, lipid storage, and energy density of *Calanus finmarchicus* and other copepod preyfields along the Northwest Atlantic continental shelf. Journal of Plankton Research 46:282–294.

Davies, K. T. A., A. Ryan, and C. T. Taggart. 2012. Measured and inferred gross energy content in diapausing *Calanus* spp. in a Scotian shelf basin. Journal of Plankton Research 34:614–625.

Ergon, T., Ø. Borgan, C. R. Nater, and Y. Vindenes. 2018. The utility of mortality hazard rates in population analyses. Methods in Ecology and Evolution 9:2046–2056.

Ganley, L. C., S. Brault, and C. A. Mayo. 2019. What we see is not what there is: Estimating North Atlantic right whale *Eubalaena glacialis* local abundance. Endangered Species Research 38:101–113.

Garrison, L. P., J. Adams, E. M. Patterson, and C. . Good. 2022. Assessing the risk of vessel strike mortality in North Atlantic right whales along the U.S East Coast. NOAA Technical Memorandum NMFS-SEFSC-757.

Hudak, C. A., K. Stamieszkin, and C. A. Mayo. 2023. North Atlantic right whale *Eubalaena glacialis* prey selection in Cape Cod Bay. Endangered Species Research 51:15–29.

Lehoux, C., P. S., and V. Lesage. 2020. Significance of dominant zooplankton species to the North Atlantic Right Whale potential foraging habitats in the Gulf of St. Lawrence: a bio-energetic approach. DFO Can. Sci. Advis. Sec. Res. Doc. 2020/033. iv + 44 p.

Lunn, D., C. Jackson, N. Best, A. Thomas, and D. Spiegelhalter. 2013. The BUGS book: A practical introduction to Bayesian analysis. Chapman & Hall/CRC, Boca Raton, Florida, USA.

Mayo, C. A., and M. K. Marx. 1990. Surface foraging behaviour of the North Atlantic right whale, *Eubalaena glacialis*, and associated zooplankton characteristics. Canadian Journal of Zoology 68:2214–2220.

Meyer-Gutbrod, E. L., K. T. A. Davies, C. L. Johnson, S. Plourde, K. A. Sorochan, R. D. Kenney, C. Ramp, J. F. Gosselin, J. W. Lawson, and C. H. Greene. 2023. Redefining North Atlantic right whale habitat-use patterns under climate change. Limnology and Oceanography 68:S71–S86.

Miller, A. S., L. K. Solinger, B. Shank, A. Huamani, M. J. Asaro, and D. Sigourney. 2025. Gearing up: Methods for quantifying gear density for fixed-gear commercial fisheries in the U.S. Atlantic. Canadian Journal of Fisheries and Aquatic Sciences 82:1–15.

Miller, A. S., L. Solinger, B. Shank, A. Huamani, M. Duffing Romero, M. Asaro, C. Franco, and M. Trego. 2024. Documenting a decision support tool to assess risk of entanglement mortality to large whales from commercial fixed-gear fisheries in the Northwest Atlantic. U.S. Department of Commerce Northeast Fisheries Science Center Technical Memorandum 312.

Pendleton, D. E., A. J. Pershing, M. W. Brown, C. A. Mayo, R. D. Kenney, N. R. Record, and T. V. N. Cole. 2009. Regional-scale mean copepod concentration indicates relative abundance of North Atlantic right whales. Marine Ecology Progress Series 378:211–225.

Pershing, A. J., C. H. Greene, J. W. Jossi, L. O'Brien, J. K. T. Brodziak, and B. A. Bailey. 2005. Interdecadal variability in the Gulf of Maine zooplankton community, with potential impacts on fish recruitment. ICES Journal of Marine Science 62:1511–1523.

Pettis, H. M., R. M. Rolland, P. K. Hamilton, S. Brault, A. R. Knowlton, and S. D. Kraus. 2004. Visual health assessment of North Atlantic right whales (*Eubalaena glacialis*) using photographs. Canadian Journal of Zoology 82:8–19.

Pirotta, E., R. S. Schick, P. K. Hamilton, C. M. Harris, J. Hewitt, A. R. Knowlton, S. D. Kraus, E. Meyer-Gutbrod, M. J. Moore, H. M. Pettis, T. Photopoulou, R. M. Rolland, P. L. Tyack, and L. Thomas. 2023. Estimating the effects of stressors on the health, survival and reproduction of a critically endangered, long-lived species. Oikos 2023:e09801.

Pirotta, E., P. L. Tyack, J. W. Durban, H. Fearnbach, P. K. Hamilton, C. M. Harris, A. R. Knowlton, S. D. Kraus, C. A. Miller, M. J. Moore, H. M. Pettis, T. Photopoulou, R. M. Rolland, R. S. Schick, and L. Thomas. 2024. Decreasing body size is associated with reduced calving probability in critically endangered North Atlantic right whales. Royal Society Open Science 11:240050.

Plourde, S., P. Joly, J. A. Runge, J. Dodson, and B. Zakardjian. 2003. Life cycle of *Calanus hyperboreus* in the lower St. Lawrence Estuary and its relationship to local environmental conditions. Marine Ecology Progress Series 255:219–233.

Plourde, S., C. Lehoux, J. J. Roberts, C. L. Johnson, N. Record, P. Pepin, C. Orphanides, R. S. Schick, H. J. Walsh, and C. H. Ross. 2024. Describing the seasonal and spatial distribution of *Calanus* species and North Atlantic right whale potential foraging habitats in Canadian waters using species distribution models. DFO Canadian Science Advisory Secretariat Research Document 2024/03.

Redfern, J. V., B. C. Hodge, D. E. Pendleton, A. R. Knowlton, J. Adams, E. M. Patterson, C. P. Good, and J. J. Roberts. 2024. Estimating reductions in the risk of vessels striking whales achieved by management strategies. Biological Conservation 290:110427.

Redfern, J. V, M. F. McKenna, T. J. Moore, J. Calambokidis, M. L. Deangelis, E. A. Becker,

J. Barlow, K. A. Forney, P. C. Fiedler, and S. J. Chivers. 2013. Assessing the risk of ships striking large whales in marine spatial planning. Conservation Biology 27:292–302.

Roberts, J. J., T. M. Yack, E. Fujioka, P. N. Halpin, M. F. Baumgartner, O. Boisseau, S. Chavez-Rosales, T. V. N. Cole, M. P. Cotter, G. E. Davis, R. A. DiGiovanni, L. C. Ganley, L. P. Garrison, C. P. Good, T. A. Gowan, K. A. Jackson, R. D. Kenney, C. B. Khan, A. R. Knowlton, S. D. Kraus, G. G. Lockhart, K. S. Lomac-MacNair, C. A. Mayo, B. E. McKenna, W. A. McLellan, D. P. Nowacek, O. O'Brien, D. A. Pabst, D. L. Palka, E. M. Patterson, D. E. Pendleton, E. Quintana-Rizzo, N. R. Record, J. V. Redfern, M. E. Rickard, M. White, A. D. Whitt, and A. M. Zoidis. 2024. North Atlantic right whale density surface model for the US Atlantic evaluated with passive acoustic monitoring. Marine Ecology Progress Series 732:167–192.

Rockwood, R. C., J. D. Adams, S. Hastings, J. Morten, and J. Jahncke. 2021. Modeling whale deaths from vessel strikes to reduce the risk of fatality to endangered whales. Frontiers in Marine Science 8:649890.

Rolland, R. M., R. S. Schick, H. M. Pettis, A. R. Knowlton, P. K. Hamilton, J. S. Clark, and S. D. Kraus. 2016. Health of North Atlantic right whales, *Eubalaena glacialis*, over three decades: from individual health to demographic and population health trends. Marine Ecology Progress Series 542:265–282.

Runge, M. C., D. W. Linden, J. A. Hostetler, D. L. Borggaard, L. P. Garrison, A. R. Knowlton, V. Lesage, R. Williams, and R. M. I. Pace. 2023. A management-focused population viability analysis for North Atlantic right whales. US Department of Commerce Northeast Fisheries Science Centre Technical Memorandum 307, 93 p.

Sorochan, K. A., S. Plourde, M. F. Baumgartner, and C. L. Johnson. 2021. Availability, supply, and aggregation of prey (*Calanus spp.*) in foraging areas of the North Atlantic right whale (*Eubalaena glacialis*). ICES Journal of Marine Science 78:3498–3520.

St-Pierre, A. P., T. Koll-Egyed, V. Harvey, J. W. Lawson, C. Sauvé, A. Ollier, P. J. Goulet, M. O. Hammill, and J.-F. Gosselin. 2024. Distribution of North Atlantic right whales, *Eubalaena glacialis*, in Eastern Canada from line-transect surveys from 2017 to 2022. DFO Can. Sci. Advis. Sec. Res. Doc. 2024/059.

Stewart, J. D., J. W. Durban, A. R. Knowlton, M. S. Lynn, H. Fearnbach, J. Barbaro, W. L. Perryman, C. A. Miller, and M. J. Moore. 2021. Decreasing body lengths in North Atlantic right whales. Current Biology 31:3174-3179.e3.

Vanderlaan, A. S. M., and C. T. Taggart. 2007. Vessel collisions with whales: The probability of lethal injury based on vessel speed. Marine Mammal Science 23:144–156.

Williams, R., and P. O'Hara. 2010. Modelling ship strike risk to fin, humpback and killer whales in British Columbia, Canada. Journal of Cetacean Research and Management:1–10.

Woodman, S. M., K. A. Forney, E. A. Becker, M. L. DeAngelis, E. L. Hazen, D. M. Palacios, and J. V. Redfern. 2019. esdm: A tool for creating and exploring ensembles of predictions from species distribution and abundance models. Methods in Ecology and Evolution 10:1923–1933.
